# Genome‐wide study links cardiometabolic factors to cognition via *APOA4‐APOA5‐ZPR1‐BUD13* and other loci in rural Indians

**DOI:** 10.1002/alz.70429

**Published:** 2025-07-15

**Authors:** Shreya Chakraborty, Krithika Subramanian, Akkshaya Rajesh, Rupanwita Majumder, Khader Valli Rupanagudi, Abhishek Mensegere, TLSA study team, Thomas Gregor Issac, SANSCOG study team, Jonas Sundarakumar, Bratati Kahali

**Affiliations:** ^1^ Centre for Brain Research Indian Institute of Science Bangalore India; ^2^ Interdisciplinary Mathematical Sciences (IMI) Indian Institute of Science Bangalore India; ^3^ Manipal Academy of Higher Education (MAHE) Manipal India

**Keywords:** ancestry‐matched imputation panel, cardiometabolic, cognition, causal association, genome‐wide association study (GWAS), India

## Abstract

**INTRODUCTION:**

Cardiometabolic risks affect cognition during aging, yet genetic basis for both remain understudied in Indians.

**METHODS:**

This study constructs an ancestry‐matched Indian haplotype reference panel for genotype imputation of 5111 rural Indians. Single‐locus, gene‐based, conditional genome‐wide association analyses are performed on 20 cognitive and 10 cardiometabolic traits, with subsequent follow‐up of identified associations through multimodal functional annotation. Furthermore, causal interrelationships between cardiometabolic and cognitive phenotypes by Mendelian randomization are investigated.

**RESULTS:**

One novel memory‐associated and 17 novel cardiometabolic phenotypes‐associated (high‐density lipoprotein cholesterol [HDL‐C], low‐density lipoprotein cholesterol [LDL‐C], triglycerides [TG], total cholesterol [TC], TG:HDL, and visceral adiposity index [VAI]) genome‐wide significant loci, and multiple genes are identified. *AMIGO1* (delayed‐recall) and *ZPR1‐APOA5* (metabolic syndrome) exhibit distinct haplotype structure compared to other populations. Causal roles of cardiometabolic traits on various cognitive domains are identified via genetic instruments in *APOC3‐APOA4‐APOA5‐ZPR1‐BUD13* among others.

**DISCUSSION:**

These findings illustrate the impact of cardiometabolic factors on cognition in a rural socioeconomically disadvantaged population, advancing efforts to address health disparities.

**Highlights:**

- Our newly constructed ancestry‐matched haplotype reference panel gives better genotype imputation accuracy for the Indian population.
- One and 17 novel genome‐wide significant single‐loci were identified to be associated with cognitive and cardiometabolic traits, respectively. Several subgenome‐wide hits for all phenotypes were identified.
- Collapsing protein truncating variants (PTVs), there were two genes identified to be associated with cardiometabolic traits at a genome‐wide level of significance, correcting for multiple phenotypes tested.
- Haplotypic differences were identified compared to 1000 Genomes superpopulations for genes influencing delayed recall and metabolic syndrome.
- Adverse causal roles of cardiometabolic traits on cognition were uncovered via genetic instruments in *APOC3‐APOA4‐APOA5‐ZPR1‐BUD13*, among others, through Mendelian randomization.

## 1 BACKGROUND

Cognitive functioning, assessed through neurocognitive tests evaluating memory, attention, language, and visuospatial abilities, is a complex trait intricately linked to aging. Although mild changes in cognitive abilities are considered a normal feature of aging, epidemiological and experimental studies suggest that cardiovascular risk factors involving aberrant brain lipid metabolism, oxidative stress, neuroinflammation, vascular reactivity, type 2 diabetes, and insulin resistance could induce cognitive dysfunction and brain‐morphology changes.^1^, ^2^

Most large‐scale population‐based genome‐wide association studies (GWAS) have emphasized general cognitive functioning (g‐factor), with limited focus on specific cognition domains.^3^, ^4^, ^5^, ^6^, ^7^ Nonetheless, understanding domain‐specific contributions to general cognitive functioning is crucial. One of the reasons is that different domains may get affected at different ages and stages of cognitive impairment during aging. Second, studies have linked different biomarkers with specific cognition domains.^8^ Different heritabilities for cognitive measures pertaining to specific domains could also mean diverse underlying genetic factors for them; thus, assessing the genetic basis for domains of cognition is imperative.^9^

While most cognition‐related GWAS have focused on the European population,^3^, ^4^ some have investigated genetics of cognition in Africans,^10^ Americans,^11^ African Americans,^7^ and Hispanics.^6^ A recent study investigated the association of already reported single nucleotide polymorphisms (SNPs) from a large‐scale GWAS on Alzheimer's disease (AD) and cognition, in single‐marker and polygenic models, on 932 elderly Indians.^5^

India is the most populous nation comprising >1.4 billion people, with diverse geographical regions, languages, religions, socioeconomic backgrounds, and genetic lineages. Nationwide studies reveal differential dementia incidence and prevalence characterized by cognitive impairment across sociodemographic contexts. Therefore, studying the genetic basis of inter‐individual differences in cognition is important to understand region‐ and ancestry‐specific effects in distinct socioeconomic strata.

This study aims to decipher distinct genetic underpinnings of cognition and cardiometabolic risks in a rural, health disparate Indian population and their causal interrelationships. We constructed an India‐specific haplotype reference panel for genotype imputation, which outperforms global panels leveraging genetic ancestry matching. The cognitive and cardiometabolic risk factors mentioned above are derived from various cognitive domains, including attention, memory, language, visuospatial skills, executive functioning, as well as cognitive screening scores, cognitive composite scores, and lipid and glycemic‐based blood biochemical measures. This is one of the first‐of‐its kind work exploring genetic risks and protective factors for cognitive functioning in light of metabolic disorders in a rural population. This work is highly significant because two thirds of the Indian population lives in villages and can potentially serve as a steppingstone for investigating aspects of preserving cognition and promoting healthy aging, studies on which are currently lacking in rural population settings.

## 2 METHODS

The overall schematic of the methods is provided in Figure S1 in supporting information, the individual components of which are described below.

### 2.1 Study participants

This analytical study comprises 5111 apparently healthy aging individuals enrolled in the Center for Brain Research ‐ Srinivaspura Aging Neuro Senescence and Cognition (CBR‐SANSCOG) cohort residing in villages of Srinivaspura taluk (subdistrict), Kolar district, in the southern state of Karnataka, India.^12^ These participants have been recruited by the CBR‐SANSCOG Field Data Collection Team using an area sampling strategy, which involved charting the catchment zones covered by the respective primary health management units in Srinivaspura, organizing awareness campaigns, and contacting individuals through phone or in‐person home visits. Individuals already diagnosed with mild cognitive impairment or dementia have not been included in the study. A total of 2464 of the recruited individuals in this study are males, and 2636 are females. All participants are between 45 and 102 years of age with a mean age of 59 years, and years of education ranging from 0 to 20 years with 41.6% of them being illiterate. Data on baseline characteristics, blood biochemistry, clinical features, and neurocognitive assessment were collected from these participants. Furthermore, genotyping was performed using DNA from their blood samples. All participants provided written informed consent (Ref no: CBR/42/IEC/2023‐24).

### 2.2 Genotype quality control

The genotyped data on 5111 individuals was obtained via GeneTitan MC Fast Scan Instrument and GeneChip array using Axiom 2.0 assay. The microarray provided genotyped data on 892,508 genetic markers. Individuals who have at least 90% non‐missing genotypes were retained for further analyses. Genetic sex of these individuals was considered in all analyses. Only biallelic autosomal genetic variants without duplicates were considered. Genetic variants with genotype missingness >2%, and not in Hardy–Weinberg equilibrium (*P* value < 1 × 10^−7^) were removed. Additionally, variants having a minor allele count < 2 were excluded. Insertions and deletions (INDELs) and structural variants with allele length > 30 bases were removed. Additionally, monomorphic variants were removed (Table S1 in supporting information).

**RESEARCH IN CONTEXT**

1. **Systematic review**: Epidemiologically, cardiometabolic risks impact cognition, but genetic links between the two are understudied in the Indian population, particularly in the rural population with low socioeconomical status and higher health disparities. Such populations are majorly underrepresented in genetic studies on cognition worldwide. Also, domain‐specific studies on cognition are limited.
2. **Interpretation**: In this study on a rural Indian cohort (*n* = 5111), we create an ancestry‐matched haplotype reference panel for genotype imputation. We identify several novel and known loci and genes influencing cognitive domains, general cognitive functioning, and cardiometabolic traits. We examine their roles in gene regulation and physiological processes in the brain and periphery, providing causal evidence linking poor metabolic health to cognitive impairment. Additionally, we identify haplotypic differences in delayed‐recall and metabolic syndrome‐associated loci compared to global superpopulations.
3. **Future directions**: The aging population is increasingly burdened by metabolic disorders. Further research into physiological mechanisms will offer promising avenues for sustaining metabolic and cognitive well‐being during aging.

### 2.3 Genotype imputation

#### 2.3.1 Creation of a new haplotype reference panel with Indian data

A reference panel of haplotypes was created by merging 601 whole genome sequences of individuals enrolled in the 1000 Genomes Project (1000G) with South Asian ancestry (SAS),^13^ and 696 whole genome sequences of individuals enrolled in the Tata Longitudinal Study of Aging (CBR‐TLSA),^14^ both aligned to GRCh38 build. After obtaining informed written consent from the study participants (Ethics Ref no: CBR/42/IEC/2022‐23), DNA extracted from the whole blood of these participants was used for whole genome sequencing (WGS). Further details with respect to library preparation and the sequencing pipeline are provided in the supporting information note. The 696 WGS samples, sequenced via paired‐end short‐read sequencing (2 × 150 bp) on the Illumina NovaSeq platform, were joint‐called (single nucleotide variants [SNVs] and INDELs), following up with rigorous quality checks to assess potential batch effects. Most of the samples demonstrated > 30X depth of coverage, with the average being 42X. The duplicate rate was ≈ 6% to 8%. The mean Phred quality score was 36. A significant proportion of samples exhibited an insert size > 400 bp, demonstrating effective paired‐end mapping. More than 95% of the reads aligned with GRCh38. Benchmarking of four Genome in a Bottle (GIAB) samples was also carried out. The GIAB consortium provides a high‐confidence truth set of SNVs, small insertions, and deletions (InDels) for the samples NA12878, NA24149, NA24143, and NA24385. The cell lines for these four samples were procured, and after DNA extraction, sequenced at CBR. Thereafter, the WGS pipeline used for the variant calling achieved ≈ 99% precision and ≈ 97% recall for these four samples compared to the high‐confidence truth set, ensuring robust variant detection across the cohort‐level analysis. The average sample call rate was > 96%. The mean genotype quality (GQ) per individual was ≈ 30, with a standard deviation of ±11.5, indicating generally high confidence in the genotype calls, given the fact that GQ20 is considered a robust threshold in assessing genotype quality as a best practice. The quality‐controlled TLSA‐WGS dataset was phased using SHAPEIT5.^15^ Further details with respect to phasing are provided in the supporting information. This quality‐controlled phased panel was merged in chunks of size 5 million bases using IMPUTE2^16^ with the 1000G^13^ SAS phased haplotypes to obtain the in‐house merged reference panel. The merged panel is composed of 28,471,101 genetic variations comprising the union of both panel‐specific and overlapping variants (Table S1).

#### 2.3.2 Imputation

The genotyped variants were divided into chunks of length 5 Mb and then imputed using the Indian population–specific reference panel by IMPUTE5.^17^ Additionally, imputation was also performed using the Haplotype Reference Consortium (HRC‐r1.1)^18^ and the Trans‐Omics for Precision Medicine (TOPMed; R3)^19^ reference panels with the aid of the Michigan Imputation Server (https://imputationserver.sph.umich.edu/). Post‐imputation, a subset of variants with moderately high imputation accuracy (*R* ^2 ^> 0.7) was considered for further analysis. To rule out wrongly imputed genotypes arising due to strand flips, AT/GC SNVs with allele frequency ranging between 40% and 60% (both inclusive) were removed. Because initially there was a minor allele count (MAC) cutoff at 2 for ≈ 5000 individuals, or 10,000 alleles, a further post‐imputation filter to exclude sites with a minor allele frequency < 0.0002 was applied. With the set of variants so obtained (16,942,745; Table S1), mean imputation accuracy (*R* ^2^) against allele frequency bins of the in‐house Indian reference panel was compared to the two above‐mentioned standardized Euro‐centric reference panels.

### 2.4 Phenotypes

This study analyzed 30 phenotypes in two categories: cognitive and cardiometabolic.

#### 2.4.1 Cognitive phenotypes

Twenty cognitive phenotypes were tested: the Hindi Mini Mental State Examination (HMSE), Computerized Assessment of Adult Information Processing (COGNITO^20^; 17 tests assessing four domains of cognition, namely attention, memory, language, and visuospatial), Trail‐Making Test (TMT‐B‐A), and G factor assessing general intelligence.

The COGNITO neuropsychological battery, developed by the National Institute of Health and Medical Research (Inserm), University of Montpellier, France, was cross‐culturally adapted (with permission) and used to suit the rural SANSCOG cohort. Tests were administered in two Indian languages—Kannada and Telugu—based on the language preference of the participant and typical scores were obtained.^20^

With respect to the COGNITO and other phenotypes, mean reaction time and TMT‐B‐A were calculated in units of time (seconds). For the other COGNITO phenotypes proportion of correct answers (unit‐free) in the cognitively discriminatory task was selected as the phenotype of interest. Fifteen component tests assessed by COGNITO, had data reported for at least 2000 individuals. These tests span attention (1/mean reaction time, auditory attention, visual attention, dual attention), memory (delayed recall, immediate recall, name recognition, name–face associations [name & face]), language (reading, comprehension, phonetic, semantic, vocabulary), executive functioning (naming associations/semantic associations), and visuospatial (span, geometric figures, Stroop) domains. Then, a factor analysis was conducted with two factors. The first factor thus obtained was the “g‐factor”: a composite score assessing general cognitive functioning. All these constituent scores were positively correlated with each other.

#### 2.4.2 Cardiometabolic phenotypes

Ten cardiometabolic phenotypes were tested: high‐density lipoprotein cholesterol (HDL‐C), low‐density lipoprotein cholesterol (LDL‐C), triglycerides (TG; log‐scaled), total cholesterol (TC), TG:HDL, fasting blood glucose (FBS) and glycated hemoglobin (HbA1c), metabolic syndrome case–control status, visceral fat, and visceral adiposity index (VAI).

Phenotypes like HDL‐C, LDL‐C, total cholesterol, fasting blood glucose, and triglycerides were measured in mg/dL from blood samples. Traits like HbA1c and visceral fat, also assessed from blood samples and anthropometric measures, were reported as percentages. This study applied natural logarithmic transformation to triglycerides (mg/DL), as in studies reported by the Global Lipids Genetics Consortium (https://csg.sph.umich.edu/willer/public/glgc‐lipids2021/). Sex‐specific VAI was calculated; VAI for males was calculated as:

- VAI = (waist circumference [cm]/[39.68 + 1.88 × body mass index (BMI)]) × (triglycerides [mg/dL]/1.03) × (1.31/HDL cholesterol [mg/dL]).

While VAI for females was calculated as:

- VAI = (waist circumference [cm]/[36.58 + 1.89 × BMI]) × (triglycerides [mg/dL]/0.81) × (1.52/HDL cholesterol [mg/dL]).

National Cholesterol Education Program Adult Treatment Panel III (NCEP‐ATP III) criteria were used to assign metabolic syndrome cases. Individuals having three or more of the below five criteria were assigned as metabolic syndrome cases.

- Fasting glucose ≥ 100 mg/dL.
- Systolic blood pressure (BP) ≥ 130 mmHg and/or diastolic BP ≥ 85 mmHg.
- TG ≥ 150 mg/dL.
- HDL‐C <40 mg/dL for men and <50 mg/dL for women.
- Waist circumference of ≥ 102 cm in men or ≥ 88 cm in women.

Further detailed information about the phenotypes is provided in the supporting information note.

### 2.5 Heritability

Narrow‐sense heritability of the continuous cardiometabolic phenotypes of interest was estimated after accounting for age, age squared, sex, top 10 principal components, using the GREML model^21^ implemented in GCTA software.^22^ For metabolic syndrome phenotype, the raw phenotype was used for heritability analysis without covariate adjustment. For the cognition‐related phenotypes, an additional control for education status apart from the above‐mentioned covariates was implemented. Based on the number of years of schooling, educational status was categorized as low (< 1 year), medium (1–8 years), and high (> 8 years). For the heritability calculation, the genome was divided into segments of 200 kb length (with 100 kb overlap between two adjacent segments) and the linkage disequilibrium (LD) scores were calculated. Then, the genetic variants were stratified into four quartiles based on their LD scores. These quartiles of variants were then used to construct multiple genetic relationship matrices (GRMs) for improved estimation accuracy by correcting for the LD bias in heritability. The heritability estimates and the standard errors are provided in Table S2 in supporting information.

### 2.6 Single variant associations

Genome‐wide association analysis by fitting a linear mixed model using the GRAMMAR‐GAMMA^23^ method was performed, taking cryptic relationships into account. The estimated genetic relatedness or kinship matrix from genotypes of 16,942,745 imputed markers was obtained. Then, rvtests^24^ was used for this analysis, in which the dependent variables were rank‐based inverse normalized residuals obtained by regressing each quantitative phenotype on age, age squared, sex, and the first 10 principal components. For cognitive phenotypes, an additional control for education status categorized as low (< 1 year), medium (1–8 years), and high (> 8 years) was implemented. For the metabolic syndrome phenotype (see phenotype section for details of case–control status in supporting information note), generalized linear mixed modeling^25^ was performed on markers with minor allele frequency (MAF) > 0.001.

In the results, independent association signals with imputation accuracy > 0.7 and standard error < 2 are reported in Table 1 as “high confidence hits” at genome‐wide level of significance (*P* < 5 × 10^−8^), while highlighting variants that also passed the phenotype‐level Bonferroni‐corrected threshold of 1.67 × 10^−9^ (5 × 10^−8^/30; Table 1). Subgenome‐wide (*P* < 5 × 10^−6^) hits are reported in Tables S3–S4 in supporting information. Independence of genetic loci was evaluated by clumping high‐confidence subgenome‐wide top lead variants with variants in the vicinity (±250kb) associated with respective phenotypes with *P* < 5 × 10^−5^ and in linkage disequilibrium (LD *R* ^2 ^> 0.7) with the lead variant. The clumping was done in a manner such that variants already clumped with lead variants with lower *P* values were not further considered lead variants, even when they satisfied the *P* value threshold criteria for being a high‐confidence hit.

**TABLE 1:** Genome‐wide significant hits with *P* < 5 × 10^−8^.

COGNITO Memory: Name Recognition; *N* = 4104										
ID	AF	Beta	SE	*p*	rsid	*R* ^2^	Consequence	Gene	CADD‐v1.7	Novel
14:85766662:C:T	0.338	0.13	1.49	2.57E‐08	rs12588896	0.967	Intergenic	*FLRT2‐LINC02328*	1.002	Yes

Lipid: HDL‐C; *N* = 3982										
ID	AF	Beta	SE	*p*	Rsid	*R* ^2^	Consequence	Gene	CADD‐v1.7	Novel
16:56953103:C:T	0.272	0.241	1.59	1.29E‐21	rs12446515*	0.95	Intergenic	*HERPUD1‐CETP*	2.108	No
16:56971389:C:T	0.469	0.189	1.42	4.74E‐17	rs1532625*	0.933	Splice&Intron	*CETP*	0.757	No
16:56961324:C:A	0.600	0.180	1.43	1.39E‐15	rs1800775*	1	Upstream	*CETP*	0.409	No
16:56951643:A:G	0.732	0.204	1.62	1.88E‐15	rs12448528*	0.903	Intergenic	*HERPUD1‐CETP*	1.462	No
16:56967362:AC:A	0.243	0.202	1.64	9.76E‐15	rs200751500*	0.908	Intron	*CETP*	–	No
16:56973534:T:G	0.240	−0.193	1.66	1.84E‐13	rs11076176*	0.904	Intron	*CETP*	2.296	No
16:56951602:G:A	0.297	0.178	1.55	3.40E‐13	rs72786786*	0.852	Intergenic	*HERPUD1‐CETP*	3.971	No
16:56957712:G:A	0.248	−0.183	1.65	2.89E‐12	rs28888131*	1	Upstream	*CETP*	1.122	Yes
16:56968820:T:G	0.779	0.187	1.70	3.91E‐12	rs9939224*	1	Intron	*CETP*	1.041	No
16:56968751:C:G	0.681	0.159	1.52	3.68E‐11	rs9926440*	0.963	Intron	*CETP*	1.643	Yes
16:56951227:A:G	0.600	0.144	1.45	3.70E‐10	rs9989419*	1	Intergenic	*HERPUD1‐CETP*	0.826	No
16:56967026:G:A	0.149	−0.197	1.99	4.16E‐10	rs118146573*	1	Intron	*CETP*	2.317	No
15:58431280:T:C	0.284	0.154	1.56	5.63E‐10	rs1077834*	1	Upstream	*LIPC*	0.225	No
16:56964660:G:C	0.332	−0.142	1.51	2.94E‐09	rs9929488	0.947	Intron	*CETP*	0.111	Yes
8:19966981:T:C	0.282	0.148	1.58	3.52E‐09	rs13702	1	3_prime_UTR	*LPL*	0.557	No
16:56955918:G:A	0.496	−0.132	1.42	4.57E‐09	rs12923459	0.99	Intergenic	*HERPUD1‐CETP*	3.673	Yes
16:56951244:A:G	0.683	0.134	1.52	2.94E‐08	rs193695	0.912	Intergenic	*HERPUD1‐CETP*	0.11	No
8:20054859:G:A	0.188	0.157	1.80	3.82E‐08	rs115849089	0.995	Intergenic	*LPL‐SLC18A1*	0.705	No

Lipid: LDL‐C; *N* = 3943										
ID	AF	Beta	SE	*p*	rsid	*R* ^2^	Consequence	Gene	CADD‐v1.7	Novel
1:109275684:G:T	0.708	0.204	1.56	1.64E‐16	rs629301	1	3_prime_UTR	*CELSR2*	0.227	No

Lipid: log TG; *N* = 3982										
ID	AF	Beta	SE	*p*	rsid	*R* ^2^	Consequence	Gene	CADD‐v1.7	Novel
11:116778201:G:C	0.781	−0.297	1.70	3.73E‐28	rs964184*	1	3_prime_UTR	*ZPR1*	0.481	No
11:116792991:G:A	0.808	−0.278	1.78	5.48E‐23	rs662799*	1	Upstream	*ZPR1*	0.931	No
11:116753987:G:T	0.696	−0.229	1.53	3.88E‐21	rs180326*	0.999	Intron	*BUD13*	0.016	No
11:116752497:TA:T	0.700	−0.220	1.54	1.47E‐19	rs66505542*	0.994	Intron	*BUD13*	−	Yes
11:116715567:T:C	0.767	−0.209	1.66	2.30E‐15	rs7350481*	1	Intergenic	*LINC02702–BUD13*	0.933	No
11:116800289:A:G	0.743	−0.196	1.60	9.54E‐15	rs4938313*	0.996	Intergenic	*APOA5‐APOA4*	0.928	No
11:116801297:T:C	0.747	−0.196	1.60	1.11E‐14	rs11216140*	0.985	Intergenic	*APOA5‐APOA4*	1.148	No
11:116799960:A:C	0.744	−0.194	1.60	1.62E‐14	rs6589567*	1	Intergenic	*APOA5‐APOA4*	1.11	No
11:116800760:T:C	0.744	−0.194	1.60	1.62E‐14	rs6589569*	0.994	Intergenic	*APOA5‐APOA4*	1.372	No
11:116793324:G:A	0.474	−0.146	1.43	1.19E‐10	rs10750097*	0.966	Upstream	*APOA5*	4.866	Yes
11:116836622:A:G	0.618	−0.146	1.47	3.22E‐10	rs7116797*	0.963	Downstream	*APOC3*	8.426	No
11:116738598:G:GCA	0.243	0.158	1.65	1.62E‐09	rs57228226*	0.79	Intergenic	*LINC02702‐BUD13*	0.608	Yes
11:116774559:C:T	0.399	−0.137	1.45	2.03E‐09	rs7118999	0.982	3_prime_UTR	*ZPR1*	0.767	Yes
11:116815326:C:CT	0.578	−0.135	1.44	3.34E‐09	rs71037424	0.992	Intergenic	*APOA5‐APOA4*	0.413	Yes
11:116813312:T:C	0.619	−0.134	1.46	6.89E‐09	rs7396835	1	Intergenic	*APOA5‐APOA4*	1.36	No
11:116821618:C:T	0.619	−0.134	1.47	7.77E‐09	rs5104	0.99	Missense#	*APOA4*	0.023	No
11:116825064:A:G	0.620	−0.133	1.47	9.12E‐09	rs7111242	0.988	Upstream	*APOA4*	2.332	No
11:116725458:C:T	0.260	−0.147	1.62	1.07E‐08	rs180365	0.987	Intergenic	*LINC02702‐BUD13*	3.522	No
11:116809702:T:C	0.623	−0.133	1.47	1.27E‐08	rs6589571	0.985	Intergenic	*APOA5‐APOA4*	0.298	No
11:116771421:C:T	0.362	−0.132	1.46	1.30E‐08	rs664059	0.995	Downstream	*ZPR1*	0.406	Yes
11:116815050:C:G	0.623	−0.133	1.47	1.33E‐08	rs6589573	0.997	Intergenic	*APOA5‐APOA4*	0.147	No
11:116810847:G:A	0.620	−0.131	1.46	1.47E‐08	rs6589572	0.992	Intergenic	*APOA5‐APOA4*	0.926	No
11:116811440:G:A	0.620	−0.131	1.46	1.49E‐08	rs7927820	0.994	Intergenic	*APOA5‐APOA4*	1.671	No
11:116813448:T:C	0.620	−0.131	1.46	1.49E‐08	rs7396851	0.999	Intergenic	*APOA5‐APOA4*	0.195	No
11:116830819:T:C	0.538	−0.127	1.42	1.93E‐08	rs4520	0.924	Synonymous	*APOC3*	2.275	Yes

Lipid: TC; *N* = 3982										
ID	AF	Beta	SE	*p*	rsid	*R* ^2^	Consequence	Gene	CADD‐v1.7	Novel
1:109278685:G:T	0.707	0.157	1.55	1.78E‐10	rs583104	0.998	Downstream	*CELSR2*	4.52	No
19:19551411:C:T	0.159	−0.178	1.93	5.96E‐09	rs17216525	1	Downstream	*CILP2*	1.356	No

Insulin Resistance marker: TG:HDL; *N* = 3982										
ID	AF	Beta	SE	*p*	rsid	*R* ^2^	Consequence	Gene	CADD‐v17	Novel
11:116778201:G:C	0.781	−0.292	1.70	2.06E‐27	rs964184*	1	3_prime_UTR	*ZPR1*	0.481	No
11:116792991:G:A	0.808	−0.288	1.78	1.65E‐24	rs662799*	1	Upstream	*ZPR1*	0.931	No
11:116753987:G:T	0.696	−0.221	1.53	1.05E‐19	rs180326*	0.999	Intron	*BUD13*	0.016	No
11:116752497:TA:T	0.700	−0.211	1.53	3.86E‐18	rs66505542*	0.994	Intron	*BUD13*	−	Yes
11:116715567:T:C	0.767	−0.199	1.66	4.29E‐14	rs7350481*	1	Intergenic	*LINC02702‐BUD13*	0.933	No
11:116801297:T:C	0.747	−0.188	1.60	1.20E‐13	rs11216140*	0.985	Intergenic	*APOA5‐APOA4*	1.148	No
11:116800289:A:G	0.743	−0.187	1.59	1.36E‐13	rs4938313*	0.996	Intergenic	*APOA5‐APOA4*	0.928	No
11:116799960:A:C	0.744	−0.185	1.60	2.53E‐13	rs6589567*	1	Intergenic	*APOA5‐APOA4*	1.11	No
11:116800760:T:C	0.744	−0.185	1.60	2.53E‐13	rs6589569*	0.994	Intergenic	*APOA5‐APOA4*	1.372	No
8:20002028:G:A	0.284	−0.153	1.58	9.25E‐10	rs2119690*	0.999	Intergenic	*LPL‐SLC18A1*	2.963	No
11:116793324:G:A	0.474	−0.138	1.43	1.17E‐09	rs10750097*	0.966	Upstream	*APOA5*	4.866	Yes
8:20054859:G:A	0.188	−0.168	1.80	4.46E‐09	rs115849089	0.995	Intergenic	*LPL‐SLC18A1*	0.705	No
11:116738598:G:GCA	0.243	0.153	1.65	5.63E‐09	rs57228226	0.79	Intergenic	*LINC02702‐BUD13*	0.608	Yes
11:116836968:A:G	0.656	−0.130	1.50	4.44E‐08	rs2070665	0.942	Downstream	*APOC3*	0.285	Yes

Lipid: VAI; *N* = 3886										
ID	AF	Beta	SE	*p*	rsid	*R* ^2^	Consequence	Gene	CADD‐v1.7	Novel
11:116778201:G:C	0.780	−0.287	1.70	7.67E‐26	rs964184*	1	3_prime_UTR	*ZPR1*	0.481	No
11:116792991:G:A	0.807	−0.286	1.78	1.24E‐23	rs662799*	1	Upstream	*ZPR1*	0.931	No
11:116753987:G:T	0.695	−0.217	1.53	9.67E‐19	rs180326*	0.999	Intron	*BUD13*	0.016	No
11:116752497:TA:T	0.700	−0.212	1.54	8.82E‐18	rs66505542*	0.994	Intron	*BUD13*	−	Yes
11:116801297:T:C	0.746	−0.197	1.60	1.74E‐14	rs11216140*	0.985	Intergenic	*APOA5‐APOA4*	1.148	No
11:116800289:A:G	0.743	−0.195	1.59	2.93E‐14	rs4938313*	0.996	Intergenic	*APOA5‐APOA4*	0.928	No
11:116799960:A:C	0.743	−0.193	1.60	4.83E‐14	rs6589567*	1	Intergenic	*APOA5‐APOA4*	1.11	No
11:116800760:T:C	0.743	−0.193	1.60	4.83E‐14	rs6589569*	0.994	Intergenic	*APOA5‐APOA4*	1.372	No
11:116715567:T:C	0.767	−0.194	1.67	3.69E‐13	rs7350481*	1	Intergenic	*LINC02702‐BUD13*	0.933	No
8:20002028:G:A	0.284	−0.145	1.58	1.09E‐08	rs2119690	0.999	Intergenic	*LPL‐SLC18A1*	*2.963*	No
11:116738598:G:GCA	0.243	0.151	1.66	1.27E‐08	rs57228226	0.79	Intergenic	*LINC02702‐BUD13*	0.608	Yes
11:116836968:A:G	0.655	−0.136	1.50	1.57E‐08	rs2070665	0.942	Downstream	*APOC3*	0.285	No

Metabolic Syndrome; *N* = 3792										
ID	AF	Beta	SE	*p*	rsid	*R* ^2^	Consequence	Gene	CADD‐v1.7	Novel
11:116778201:G:C	0.782	−0.337	0.06	2.24E‐08	rs964184	1	3_prime_UTR	*ZPR1*	0.481	No

*Notes*: *P* < 1.67 × 10^−9^: Phenotype and genotype‐level Bonferroni‐corrected threshold. Variants with *P* value less than this stringent threshold (indicated by * in rsid column) have been considered instruments for our Mendelian randomization analysis.

Abbreviations: AF, allele frequency; CADD, Combined Annotation Dependent Deletion;  HDL‐C, high‐density lipoprotein cholesterol; LDL‐C, low‐density lipoprotein cholesterol; SE, standard error; TC, total cholesterol; TG, triglycerides; VAI, visceral adiposity index.

Novelty of variants was assessed using LDTrait (https://ldlink.nih.gov/?tab=ldtrait). Variants were considered novel if no variant in high LD (*r* ^2^ > 0.8) within ±250 kbp had been previously associated with the trait of interest in the European Bioinformatics Institute GWAS catalog.

### 2.7 Functional annotations

SnpEff 5.0^26^ and VEP^27^ were used to identify putative consequences of variants present in our dataset. SnpEff reports multiple annotations sorted based on higher putative impact, extent of deleteriousness, canonical transcripts before non‐canonical ones, and genomic co‐ordinates; in this study, the first annotation was considered for downstream analysis. The effect/sequence‐ontology terms reported by SnpEff were used to generate masks for collapsing genetic variants to perform subsequent gene‐based tests.

### 2.8 Expression quantitative trait loci colocalization

At first, we investigated whether the GWAS hits were expression quantitative trait loci (eQTLs) in relevant tissues by querying in Genotype‐Tissue Expression (GTEx)^28^ database. Next, a neighborhood of ±250kb was considered around the independent hits (Tables S3–S4) and cis‐eGenes were fetched using GENCODE (release 45 GRCh38.p14) and to identify their respective eQTLs from GTEx in brain, liver, adipose subcutaneous, and adipose visceral omentum. The normalized effect size was obtained, and the standard errors were calculated to perform colocalization analysis using COLOC.^29^ The hits were considered colocalized if the posterior probability (PP) of a shared causal variant was > 0.8 (Table S5 in supporting information).

### 2.9 Hi‐C–based chromatin interaction analysis

To check if association signals reside in regions of chromatin–chromatin interactions and thereby regulate gene expression, Hi‐C and Capture‐C datasets on loops from GEO^30^ (Gene Expression Omnibus) were obtained from three brain tissues (posterior cingulate gyrus, caudate nucleus, and dorsolateral prefrontal cortex) of European ancestry individuals (79–80 years) and Hep‐G2 cell lines (GEO dataset IDs, sample IDs, and relevant additional details are in supporting information section “Hi‐C datasets used for investigating spatial overlap of chromatin loops with association signals”). A “clumped‐set‐for‐plausible‐interactions” between association signals and Hi‐C loops was created by clumping subgenome‐wide association signals (*P* value < 5 × 10^−6^) and their respective correlated markers (within ±250kb with LD *R* ^2 ^> 0.7), which were associated with the trait of interest with *P* value < 5 × 10^−5^. Variants in the clumped set that fall in the bait region were identified, and genes with putative transcription start site (TSS) located in the corresponding interacting region were noted. Similarly, variants that reside in the interacting region were identified, and genes with TSS in the bait region were also noted. By virtue of this exercise, the aim was to establish if subgenome‐wide independent hits identified from the study within the corresponding “clumped‐set‐for‐plausible‐interactions” physically interact with the genes in the chromatin loop—forming a potential functional link between the genetic variant and gene expression regulation, thus annotating variants for vital functional consequences (Tables S6–S8 in supporting information).

### 2.10 Conditional association

Conditional genome‐wide association analyses, conditioning on the identified high‐confidence genome‐wide association signals (listed in Table 1), were performed to identify secondary independent signals associated with the phenotypes under consideration using GCTA‐COJO^31^ with default parameters.

### 2.11 Gene‐based associations

SKATO‐ACAT gene‐based tests were performed using REGENIE^32^ by collapsing genetic variants based on two categories: (1) residing in coding regions and (2) protein‐truncating variants (PTVs). Here, the aim was to test coding variants and PTVs which are putatively rare variants to analyze their combined effect on the trait of interest. REGENIE was used for this analysis because it incorporates omnibus tests like SKATO‐ACAT that are more powerful for rare variants, and by design, the program takes genetic relatedness into account. The SNPEff annotations used to collapse variants and create masks are given in Table S9 in supporting information. Similar to single variant association, here again for the 29 quantitative phenotypes, inverse normalized residuals were used, controlling for the confounders mentioned before. Coding variants mapped to 17,145 genes and protein truncating variants mapped to 16,581 genes, resulting in multiple phenotype corrected genome‐wide significant thresholds at ≈ 7.01 (–log_10_[0.05/(17145*30)]) and ≈ 7.00 (–log_10_[0.05/(16581*30)]), respectively (Table S10 in supporting information). In the results, subgenome‐wide significant (*P* < 10^−5^) genes are reported as “subgenome‐wide genic hits” (Tables S11–S12 in supporting information).

### 2.12 Gene‐set enrichment analysis

With the gene‐based signals at subgenome‐wide level of significance (*P* < 10^−5^) listed in Tables S11–S12, gene‐set enrichment analysis was performed against three pathway databases, Kyoto Encyclopedia of Genes and Genomes (KEGG; 2021), Reactome (2022), and Synaptic Gene Ontologies (SynGO; 2024) using Enrichr^33^ and FUMA GWAS GENE2FUNC^34^ (using protein coding genes as background gene set, maximum adjusted *P* value being 0.05 with a minimum three overlapping genes with gene sets; Table S13 in supporting information).

### 2.13 Mendelian randomization

Before proceeding to causal analysis, genetic correlation was estimated using the transformed phenotypes (as described in section 2.5) by the bivariate GREML^35^ method implemented in GCTA software. The significance of genetic correlation was tested using a likelihood ratio test.

Considering the high confidence genome‐wide hits that also crossed the phenotype‐level Bonferroni‐corrected threshold (1.67 × 10^−9^) for each of the cardiometabolic traits (HDL‐C, logTG, VAI, TG:HDL) as instruments, one‐sample Mendelian randomization (MR) was performed to assess causal connection between cardiometabolic and cognition‐related phenotypes. MR is a powerful technique in which genetically associated variants are leveraged as instrumental variables associated with trait denoting exposure to establish its causal connection to the response variable (outcome trait), under specific assumptions known as the INSIDE assumptions. The INSIDE assumptions state that the instrumental variable (genetic variant): (1) must be associated with the risk factor, (2) must not be influenced by any factors that confound the relationship between the risk factor and the outcome, and (3) must not have a direct effect on the outcome once the risk factor and confounders are controlled for.^36^ In this study, the cardiometabolic traits were the “exposures” whose causal influences on cognitive functioning were being assessed. For this analysis, inverse variance weighted regression with a multiplicative random effects model was used, which is asymptotically equivalent to two‐stage least squares technique when instruments are uncorrelated. This method combines Wald ratios (the ratio of the beta coefficient for the association between the genetic variant and the outcome to the beta coefficient for the association between the genetic variant and the exposure), with each ratio weighted by the inverse of the variance of the association between the variant and the outcome, while accounting for heterogeneity. The calculated $Fstatistic=\left(\frac{N-k-1}{K}\right)\;x\left(\frac{R^{2}}{1-R^{2}}\right)$ ^37^ tests the strength of the instrumental variables considered, where *k* is the number of instruments and *R* ^2^ is the proportion of variance in exposure explained by the instruments, and *N* is the sample size of the exposure GWAS. For this analysis, cognitive phenotypes with a sample size > 3000 and exposures with > 2 instruments were considered. Consequently, 14 outcomes were tested against four exposures and vice versa (to account for reverse causality). Hence, we apply the Bonferroni‐corrected threshold of *P* ≈ 0.00045 (i.e., 0.05/112). Adverse causal associations for which the strength of the instrumental variables was high, that is, *F* > 10 (Table 2), are highlighted in section 3. Additionally, Cochran's Q test was used to evaluate potential heterogeneity of the MR estimates (Table S14 in supporting information). To assess for horizontal pleiotropy, MR Egger regression was performed to see if the intercept deviates from zero or not (Table S15 in supporting information). The Steiger's directionality test^38^ was also performed to confirm the causal direction between metabolic traits and cognitive functioning (Table S16 in supporting information).

**TABLE 2:** Mendelian randomization results: Inverse variance weighted (multiplicative random effects) causal effects.

Outcome	Exposure	*N*_SNP_	*β*_(causal)_	SE	*P*	SNP‐*R* ^2^ exposure	SNP‐Exposure *N*	*F*	Direction of effect (if significant)
Auditory attention	HDL‐C	13	−0.012	0.033	7.30E‐01	0.178	3982	66.05	−
Auditory attention	logTG	12	0.099	0.024	3.36E‐05	0.201	3982	83.15	Protective
Auditory attention	TG:HDL	11	0.103	0.024	2.02E‐05	0.185	3982	81.94	Protective
Auditory attention	VAI	9	0.125	0.013	2.37E‐23	0.168	3886	87.05	Protective
Comprehension	HDL‐C	13	−0.049	0.034	1.51E‐01	0.178	3982	66.05	−
Comprehension	logTG	12	−0.041	0.031	1.90E‐01	0.201	3982	83.15	−
Comprehension	TG:HDL	11	−0.026	0.039	5.11E‐01	0.185	3982	81.94	−
Comprehension	VAI	9	−0.058	0.032	6.63E‐02	0.168	3886	87.05	−
Delayed recall	HDL‐C	13	0.006	0.032	8.61E‐01	0.178	3982	66.05	−
Delayed recall	logTG	12	0.116	0.019	2.89E‐09	0.201	3982	83.15	Protective
Delayed recall	TG:HDL	11	0.131	0.018	1.18E‐12	0.185	3982	81.94	Protective
Delayed recall	VAI	9	0.137	0.016	4.67E‐17	0.168	3886	87.05	Protective
Geometric figures	HDL‐C	13	0.126	0.032	7.47E‐05	0.178	3982	66.05	Adverse
Geometric figures	logTG	12	0.17	0.025	5.80E‐12	0.201	3982	83.15	Protective
Geometric figures	TG:HDL	11	0.193	0.016	2.55E‐34	0.185	3982	81.94	Protective
Geometric figures	VAI	9	0.201	0.014	5.11E‐44	0.168	3886	87.05	Protective
Immediate recall	HDL‐C	13	−0.013	0.026	6.06E‐01	0.178	3982	66.05	−
Immediate recall	logTG	12	0.18	0.02	5.23E‐20	0.201	3982	83.15	Protective
Immediate recall	TG:HDL	11	0.188	0.02	5.64E‐21	0.185	3982	81.94	Protective
Immediate recall	VAI	9	0.203	0.015	1.18E‐43	0.168	3886	87.05	Protective
MRT	HDL‐C	13	−0.04	0.04	3.11E‐01	0.178	3982	66.05	−
MRT	logTG	12	0.075	0.013	9.76E‐09	0.201	3982	83.15	Adverse
MRT	TG:HDL	11	0.093	0.015	3.35E‐10	0.185	3982	81.94	Adverse
MRT	VAI	9	0.084	0.01	4.28E‐17	0.168	3886	87.05	Adverse
Name recognition	HDL‐C	13	0.003	0.032	9.27E‐01	0.178	3982	66.05	−
Name recognition	logTG	12	0.167	0.016	5.02E‐25	0.201	3982	83.15	Protective
Name recognition	TG:HDL	11	0.164	0.016	1.30E‐23	0.185	3982	81.94	Protective
Name recognition	VAI	9	0.165	0.017	1.78E‐22	0.168	3886	87.05	Protective
Name–face associations (face)	HDL‐C	13	0.127	0.035	3.02E‐04	0.178	3982	66.05	Adverse
Name–face associations (face)	logTG	12	−0.084	0.016	1.86E‐07	0.201	3982	83.15	Adverse
Name–face associations (face)	TG:HDL	11	−0.078	0.019	5.60E‐05	0.185	3982	81.94	Adverse
Name–face associations (face)	VAI	9	−0.079	0.019	4.99E‐05	0.168	3886	87.05	Adverse
Phonetic	HDL‐C	13	−0.072	0.029	1.30E‐02	0.178	3982	66.05	−
Phonetic	logTG	12	0.055	0.015	3.09E‐04	0.201	3982	83.15	Protective
Phonetic	TG:HDL	11	0.055	0.019	3.31E‐03	0.185	3982	81.94	Protective
Phonetic	VAI	9	0.051	0.01	1.59E‐07	0.168	3886	87.05	Protective
Reading	HDL‐C	13	0.06	0.031	5.28E‐02	0.178	3982	66.05	−
Reading	logTG	12	0.163	0.019	1.74E‐17	0.201	3982	83.15	Protective
Reading	TG:HDL	11	0.175	0.009	8.77E‐81	0.185	3982	81.94	Protective
Reading	VAI	9	0.172	0.011	1.44E‐53	0.168	3886	87.05	Protective
Semantic	HDL‐C	13	0.008	0.02	6.85E‐01	0.178	3982	66.05	−
Semantic	logTG	12	0.13	0.012	1.45E‐27	0.201	3982	83.15	Protective
Semantic	TG:HDL	11	0.133	0.014	5.09E‐23	0.185	3982	81.94	Protective
Semantic	VAI	9	0.134	0.015	7.10E‐19	0.168	3886	87.05	Protective
Naming association (semantic)	HDL‐C	13	−0.121	0.034	3.23E‐04	0.178	3982	66.05	Protective
Naming association (semantic)	logTG	12	0.17	0.014	5.68E‐36	0.201	3982	83.15	Protective
Naming association (semantic)	TG:HDL	11	0.179	0.014	5.05E‐38	0.185	3982	81.94	Protective
Naming association (semantic)	VAI	9	0.185	0.012	5.47E‐50	0.168	3886	87.05	Protective
Span	HDL‐C	13	0	0.018	9.78E‐01	0.178	3982	66.05	−
Span	logTG	12	0.07	0.028	1.31E‐02	0.201	3982	83.15	−
Span	TG:HDL	11	0.073	0.031	1.76E‐02	0.185	3982	81.94	−
Span	VAI	9	0.058	0.03	5.06E‐02	0.168	3886	87.05	−
Visual	HDL‐C	13	0.004	0.028	8.77E‐01	0.178	3982	66.05	−
Visual	logTG	12	0.049	0.019	8.96E‐03	0.201	3982	83.15	Protective
Visual	TG:HDL	11	0.056	0.018	1.91E‐03	0.185	3982	81.94	Protective
Visual	VAI	9	0.056	0.015	2.83E‐04	0.168	3886	87.05	Protective

*Note*: –, Causal associations not significant post Bonferroni correction.

Abbreviations: HDL‐C, high‐density lipoprotein cholesterol; LDL‐C, low‐density lipoprotein cholesterol; MRT, mean reaction time; SE, standard error; TC, total cholesterol; TG, triglycerides; VAI, visceral adiposity index.

MR analysis considering constituent variants of genes deemed to be genome‐wide significant from the gene‐based tests as instruments was also conducted.

### 2.14 Haplotype‐based analysis of novel variants

This analysis was based on both known and novel genetic variants that contribute a cumulative burden to yield genome‐wide significance in gene‐based association (cholestereryl ester transfer protein [*CETP*], apolipoprotein E [*APOE*], apolipoprotein A [*APOA*], zinc finger protein 1 [*ZPR1*], and transmembrane 6 super family 2 [*TM6SF2*]) with cardiometabolic phenotypes of interest, when collapsing coding/PTV variants. Because these genes have been implicated in cardiometabolic phenotypes, the goal was to test whether haplotypes formed by these variants (pruned set with pairwise LD not exceeding 0.2) were associated with the metabolic syndrome phenotype. Haploview^39^ was used to conduct this analysis. The case–control haplotype frequencies were compared, and the case–control chi‐square statistic was used to test for haplotype association. Next, the haplotype structure and frequency were compared to three 1000G superpopulations, namely European (most studied), African (most diverse), and South Asian (related genetic ancestry) to assess if the haplotype structures in the Indian population is distinct from the three superpopulations or not (Table 3).

**TABLE 3:** Haplotype association results.

Phenotype: Delayed recall (< 30% correct answers—cases; ≥ 30% correct answers—controls)						
Blocks	Haplotype	Haplotype frequency	Case, control ratio counts	Case, control frequencies	Chi square	*p* value
**Block 1 (*AMIGO1*)** rs1009437 (G/A), rs3738772 (C/A)^a^, rs3738773 (G/A)^a^ rs3738774 (G/C)	GCGG	0.425	1388.0: 1770.0, 2045.0: 2825.0	0.440, 0.420	3.007	0.0829
	GCGC	0.279	890.5: 2267.5, 1330.3: 3539.7	0.282, 0.273	0.749	0.3869
	AAAG	0.216	625.5: 2532.5, 1110.3: 3759.7	0.198, 0.228	10.104	0.0015

Phenotype: Metabolic Syndrome (NCEP ATP III criteria) case–control status						
Block	Haplotype	Freq.	Case, control ratio counts	Case, control frequencies	Chi square	*P* value
**Block 1** **(*CETP*)** rs118146573 (G/A), rs1055333734 (C/G), rs9939224 (T/G), 16:56971389 (C/T)	GGGT	0.468	1002.0: 1204.0, 2556.0: 2802.0	0.454, 0.477	3.269	0.0706
	GGGC	0.217	476.0: 1730.0, 1124.0: 4234.0	0.216, 0.210	0.337	0.5617
	ACTC	0.148	333.0: 1873.0, 783.0: 4575.0	0.151, 0.146	0.288	0.5914
	GCGC	0.097	228.0: 1978.0, 519.0: 4839.0	0.103, 0.097	0.739	0.3898
	GCTC	0.071	167.0: 2039.0, 376.0: 4982.0	0.076, 0.070	0.716	0.3973
**Block 2**: **(*ZPR1*, *APOA5*)** rs964184 (C/G), rs10750097^a^ (G/A)	CA	0.463	980.9: 1233.1, 2578.9: 2791.1	0.443, 0.480	8.713	0.0032
	CG	0.319	649.1: 1564.9, 1711.1: 3658.9	0.293, 0.319	4.738	0.0295
	GG	0.214	571.9: 1642.1, 1057.9: 4312.1	0.258, 0.197	34.917	3.44E‐09^b^

Abbreviation: NCEP ATP III, National Cholesterol Education Program Adult Treatment Panel III.

^a^ Novel.

^b^ Permutation *P* value drops to ≈ 0.

Additionally, some of the gene‐based hits‐ *AMIGO1*, associated with delayed recall, overlapped with a previous study.^40^ Haplotype block association of independent variants in 3′UTR *AMIGO1* (using the same pruning criteria as above), which provides cumulative burden, was also performed with delayed recall scores. Because Haploview works with only case–control phenotypes, the continuous phenotype was categorized into two categories—low if the recall rate (proportion of correct answers) was < 0.3 and normal if ≥ 0.3 (Table 3).

## 3 RESULTS

### 3.1 Ancestry‐matched Indian haplotype reference panel showed improved imputation performance

**FIGURE 1:**
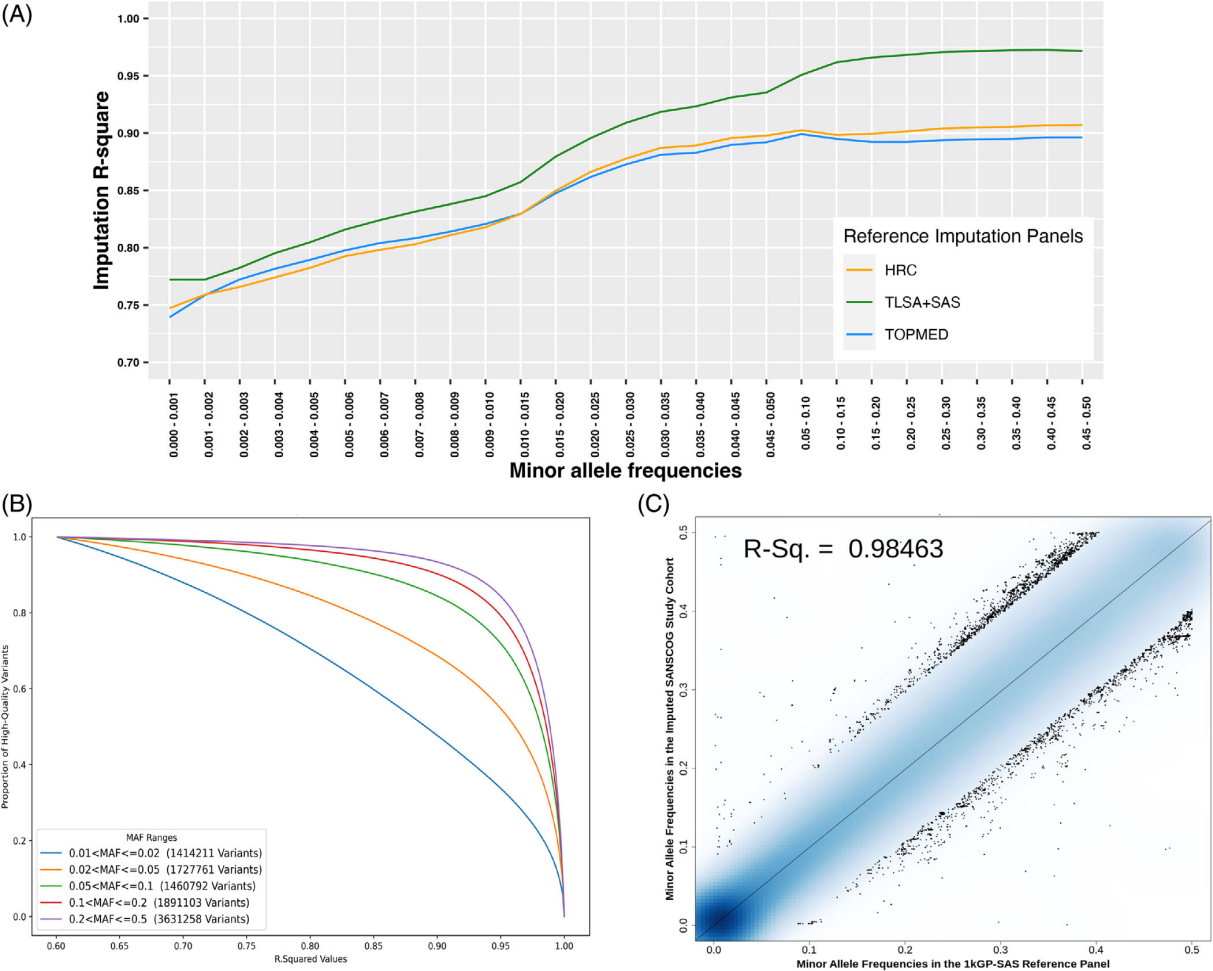
Imputation panel performance of our ancestry‐matched panel. A, Imputation accuracy (*R* ^2^) comparison across MAF bins, or in other words, rare (MAF < 1%), low frequency (MAF between 1% and 5%), and common variants (MAF > 5%). B, Coverage plots showing proportion of high‐quality variants against their mean imputation accuracy values across gradients of low and common variants (depicted in different colors). C, MAF concordance calculated with ≈ 14.81 million overlapping variants between the imputed variants and the 1000G SAS dataset. 1000G, 1000 Genomes project; HRC, Haplotype Reference Consortium; MAF, minor allele frequency; SAS, South Asian ancestry; TLSA, Tata Longitudinal Study of Aging; TOPMed, Trans‐Omics for Precision Medicine.

The merged haplotypes were derived from whole genome sequence–derived genotypes of high confidence markers from two cohorts: (1) 21,865,627 genetic markers from 601 individuals enrolled in the 1000G project with SAS and (2) 20,979,495 genetic markers from 696 individuals enrolled in the TLSA cohort (details in supporting information). This resulted in an in‐house imputation reference panel with 28,439,944 high‐confidence genetic markers, after implementing quality checks (details in supporting information). After applying post‐imputation checks, 16,942,745 high‐quality variants with an imputation accuracy > 70% were considered for association with phenotypes. The in‐house haplotype reference panel consistently outperformed the HRC and the TOPMed imputation panels across rare, low‐frequency and common variants (Figure 1A). At least 80% of the high‐quality imputed variants (across low and common variants) showed high imputation accuracy (> 75%; Figure 1B). Ninety‐eight percent concordance in the MAF distributions of ≈ 14.81 million overlapping loci was observed between our imputed target genotypes and observed genotypes in 1000G SAS (Figure 1C). Using imputed genotypes on ≈ 16 million genetic markers from the improved panel, genotype–phenotype associations were tested.

### 3.2 GWAS identified 1 and 73 independent high‐confidence genome‐wide significant loci for memory and 7 cardiometabolic phenotypes, respectively

Table 1 lists high‐confidence genome‐wide significant loci associated with one cognition and seven cardiometabolic phenotypes. rs12588896 (*β* = 0.130, standard error [SE] = 1.490, *P* = 2.57E‐08, imputation‐*R* ^2 ^= 0.967), a novel variant at genome‐wide significance, was associated with a cognitive test assessing recognition memory. This variant lies in an intergenic region between *FLRT2* and *LINC02328*. Although this variant was genome‐wide significant by traditional standards, this variant failed to attain the Bonferroni threshold for multiple phenotypes after genome‐wide significance. Additionally, several novel high‐confidence independent genetic variants at a subgenome‐wide level of significance (*P* < 5 × 10^−6^) were uncovered across all cognition phenotypes, none previously associated with cognition (Table S3). All these hits are intronic or intergenic. Additional novel interesting hits falling just short of standard genome‐wide significance were also observed. For instance, rs2111799 (intergenic *DAOA‐AS1‐LINC00343*) with HMSE, rs12534627 (*MAGI2‐AS3‐GNAI1*) with name recognition, rs68009296 (intergenic *SLC15A1‐DOCK9*) with visuospatial span, and so forth (Table S3).

This study found 73 independent (17 novel) genome‐wide significant (*P* < 5 × 10^−8^) association signals across seven cardiometabolic traits, namely HDL‐C, LDL‐C, TG, TC, TG:HDL, VAI, and metabolic syndrome (Table 1). Significant overlap exists among these signals (Figure S2 in supporting information). The top HDL‐associated locus was *HERPUD1*‐*CETP*. Ten genome‐wide significant loci overlapped between triglycerides, TG:HDL, and VAI, with the top being rs964184 in the 3′UTR of *ZPR1*. This variant was also associated with metabolic syndrome at genome‐wide significance, but failed to attain the phenotype‐corrected Bonferroni threshold. Several high‐confidence subgenome‐wide significant hits were found to be associated with 10 cardiometabolic phenotypes tested (Table S4), a few of which are missense, previously associated with cardiometabolic traits. For example, missense *APOA4* rs5104 (S[Ser] > N[Asn]) ‐ associated with TG:HDL (Table S4) and triglycerides (Table S4); the missense rs5882(V[Val] > I[Ile]) in *CETP* associated with HDL‐C (Table S4), and *GCKR* missense and splice variant rs1260326 (L[Leu] > P [Pro]) associated with triglycerides (Table S4). Total cholesterol associated rs583104 (Table 1) is itself an eQTL controlling expression of *PSRC1* in the frontal cortex BA9 region, and also colocalized with other eQTLs (PP > 99.99%) of *PSRC1* controlling its expression in putamen basal ganglia and cortex (Table S5). Additionally, novel loci in these genes were also found to be associated with the cardiometabolic traits of interest in this study. Apart from novel loci, many signals in previously known loci were also observed, for instance *CETP* in HDL‐C; *ZPR1*, *BUD13, APOA4, APOA5*, and *GCKR* in triglycerides and related traits; *CELSR2* in LDL‐C and total cholesterol.

**FIGURE 2:**
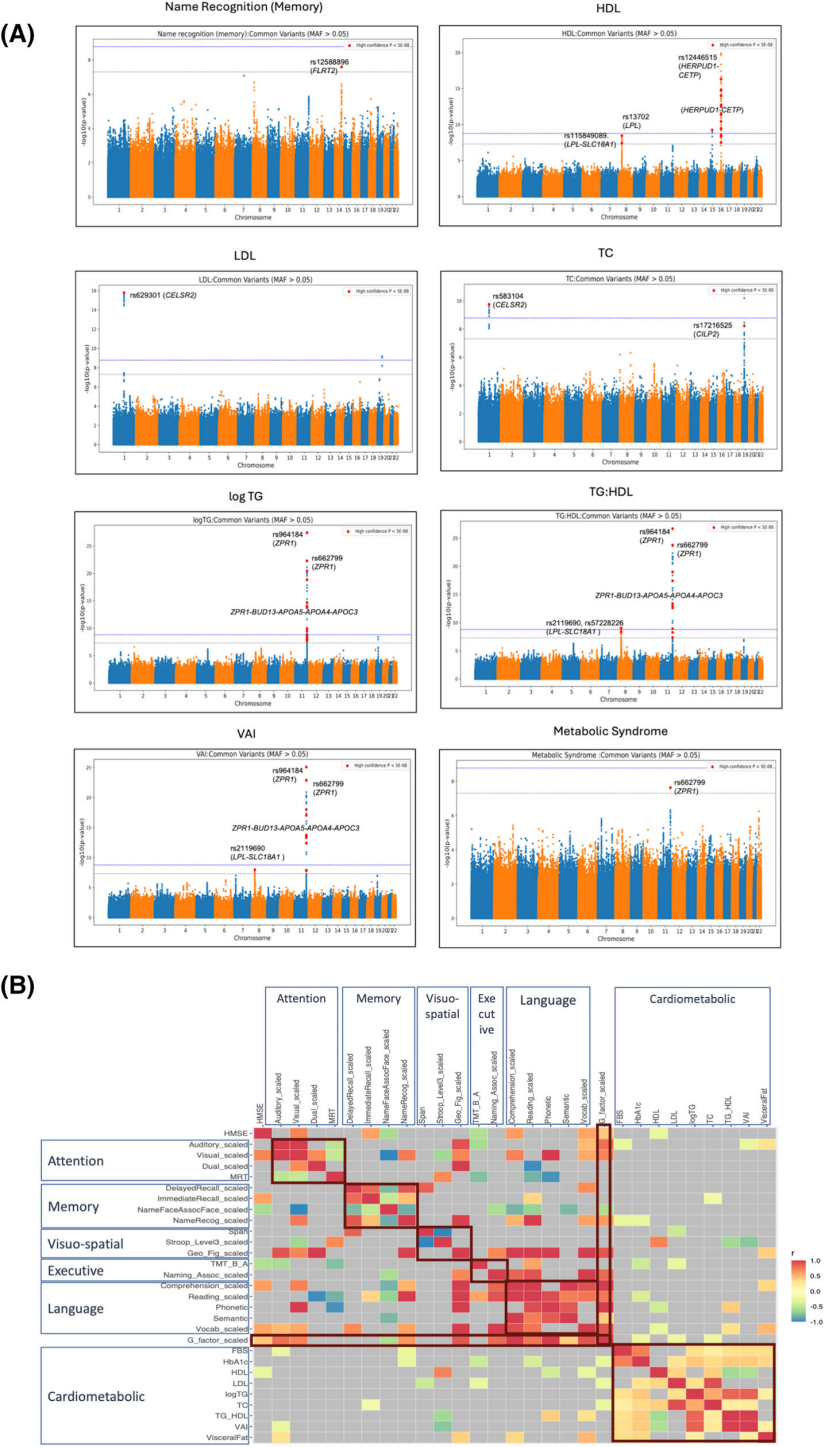
Manhattan plots for genome‐wide independent signals, signals with *P* < 5 × 10^−8^, and genetic correlations. A, Manhattan plots for recognition memory, HDL‐C, LDL‐C, total cholesterol, triglycerides, TG:HDL, VAI and metabolic syndrome. Genome‐wide (*P* < 5 × 10^−8^) high confidence independent hits with minor allele frequency > 0.05, as reported in Table 1 are highlighted in red and some of the interesting hits are annotated with rsid and gene name. B, Genetic correlations between 29/30 cardiometabolic and cognitive phenotypes assessed. Significant positive correlations within cognition domains, but low or negative correlation between cardiometabolic and cognition related phenotypes can be observed. Gray boxes indicate that the correlation is non‐significant.  HDL‐C, high‐density lipoprotein cholesterol; LDL‐C, low‐density lipoprotein cholesterol; TC, total cholesterol; TG, triglycerides; VAI, visceral adiposity index.

The genomic inflation factor for all 30 associations ranged between 0.97 and 0.99, proving that false positive associations were in check. Consequently, none of the QQ plots (Figure S3 in supporting information) were inflated. Manhattan plots for the traits with high confidence for common variants (MAF > 0.05) and genome‐wide hits are given in Figure 2A. The corresponding plots for rare and low‐frequency variants for phenotypes shown in Figure 2A are given in Figure S4 in supporting information. Manhattan plots with interesting and replicating signals for some of the cognitive traits are shown in Figure S5 in supporting information.

Genetic variants in genomic regions associated with cognition and AD were also identified, which showed notable signals in the Manhattan plot. These points have been highlighted in Figure S5. A strong signal is visible in the *ELAVL1‐CCL25‐ANGPTL4* region associated with recognition memory. Though these signals are not high confidence as per the definition in this study, they play important neuronal roles. Neuronal *ELAV* genes play a role in memory formation and neurodegenerative pathologies such as AD.^41^ *ANGPTL4* is associated with white matter hyperintensities and vascular cognitive impairment.^42^ Such strong biological signals could be estimated with greater robustness in future studies with larger sample size having higher statistical power.

Further, a signal in *DDX1* was seen to be associated with delayed recall. *DDX1* has been identified as an ADRD (Alzheimer's Disease and Related Dementia) risk‐associated gene.^43^ Another such signal was observed in the *CNTN5* gene associated with executive function. This gene encodes a contactin and contactins are neural cell adhesion proteins that play a role in developing the central nervous system and also in neuronal injury and damage and have been implicated in AD etiology.^44^ Similarly, a consistent signal associated with visuospatial abilities (geometric figures) was observed in the *GFRAL* gene (Figure S5). *GFRAL* is a receptor of *GDF15* which is implicated in several neurodegenerative diseases and with poor cognitive performance.^45^ Although not reaching the desired level of significance imposed by the small sample size, these regions pique our interest for their logical links to cognition and AD and could be high‐confidence hits in a future larger study.

Additionally, no phenotype showed evidence of secondary association signals from the conditional analysis.

### 3.3 Coding and protein truncating genetic variants implicated in cognitive and cardiometabolic traits through gene‐based associations

For the cognitive phenotypes, no gene‐based associations reached genome‐wide significance after Bonferroni correction (cutoff mentioned in the Methods section). However, 12 genes harboring enrichment of coding variants showed subgenome‐wide significant associations (*P* < 10⁻^5^), including *H2BC14* (auditory attention), *TNNI3* (visual attention), *FAM47E‐STBD1* (semantic association), *AMIGO1* and *CPZ* (delayed recall), *ZNF500* (Stroop test), *MAF* (G‐factor), *CPT2*, *MELTF*, *SLC26A4*, *PNP*, and *APP* (TMT‐B‐A test; Table S11). All these associations were driven by the aggregation of rare variants, except for two genes: *AMIGO1*, driven by the aggregation of common variants, and *FAM47E‐STBD1*, by the aggregation of low‐frequency variants. However, with respect to the cardiometabolic traits, two genome‐wide significant coding set‐based associations were identified after Bonferroni correction: *TM6SF2* with TC and VAI, and *BACE1* with VAI (Table S10). Seventeen subgenome‐wide coding set–based genes, including *TM6SF2, BACE1* and *APOE, SMPD1, GCKR*, *BAZ1B, APOA5*, *BUD13, ZPR1*, *UBR5*, *CETP, LPL*, among others, were associated with TC, triglycerides, HDL‐C, TG:HDL, and VAI (Table S12). All these genes are extremely well‐known genes implicated in lipid transport, metabolism, glucose metabolism, and related to amyloid pathology. Analysis restricted to PTVs revealed subgenome‐wide significant associations of *MVB12B* and *SURF2* (collapsing low‐frequency variants) with mean reaction time, *KCTD20* and *NEDD4* (collapsing rare variants) with phonetics, and *CHPT1* (collapsing rare variants) with vocabulary (Table S11). Considering cardiometabolic traits, two Bonferroni‐corrected genome‐wide significant PTV‐based associations were identified: *TM6SF2* (collapsing common variants) with triglycerides, TC and VAI, and *CFTR* (collapsing rare variants) with TC (Table S10). Apart from these, PTV‐based subgenome‐wide significant associations of ‐ *GLP1R* with visceral fat; *TM6SF2* and *LPL* with TG:HDL and HDL; *NXNL2* with TG:HDL and VAI; *CETP* with HDL; *CFTR* with HDL and TC; *HOXA5*, *MRE11*, *SDC4* with TC; *GCKR*, *BAZ1B*, *APOA5*, *BUD13*, *ZPR1* with triglycerides (Table S12) were also identified.

One of our previous works also posits a role of *AMIGO1* in episodic memory.^40^

Several of the lipid‐associated genic hits like *CETP*, *GCKR*, and *GLP1R* are known to function in lipid metabolism, glucose metabolism, type 2 diabetes, and insulin resistance. Some, like *TM6SF2*, previously known to function in type 2 diabetes and non‐alcoholic fatty liver disease (NAFLD),^46^ have been implicated in insulin resistance (TG:HDL) in this study, providing further evidence regarding insulin sensitivity during diabetes in the Indian population. Cardiometabolic phenotype‐associated genes, like *SCRN1*, is a candidate biomarker for AD, reflecting tau pathology; *APOE*, is a major apoprotein of the chylomicron, functioning in the catabolism of triglyceride rich lipoproteins and lipoprotein mediated lipid transport, and is the well‐known genetic risk factor for AD; and *FZD9*, contributes to neuromuscular junction assembly by inhibiting the clustering of acetylcholine receptors via the beta‐catenin canonical signaling pathway. Coupled with functional and other evidence, these also hint at the influence of cardiometabolic traits on brain functioning by virtue of genes functioning in the brain as well as peripheral organs, thereby influencing pathways linked to lipid homeostasis and neuronal function, impairment of which can lead to tau‐related neurodegeneration. Understanding the intricate interplay among metabolism, insulin resistance, and cognition is thus essential for developing interventions to manage these conditions and enhance overall well‐being during aging.

### 3.4 Spatial overlap of association signals with chromatin loops identified from Hi‐C datasets in brain tissues and HepG2 cell lines

This study found seven cognition‐associated variants (all novel) with evidence of chromatin–chromatin interaction in brain tissues with TSS of several genes (Table S6), some of which have neuronal functions. Mean reaction time associated novel rs3920616 (intergenic *CACHD1‐RAVER2*) showed evidence of interaction with TSS of *RAVER2*. Aberrant activity of *RAVER2* causes spliceopathy and demyelination in neurodegenerative diseases like autosomal dominant leukodystrophy (ADLD).^47^ Phonetics‐associated rs3740688 (intronic *SPI1*) showed evidence of interaction with TSS of *MADD, PACSIN3*, and *NR1H3/LXRA*. rs3740688 has previously been associated with AD. *MADD* interacts with tumor necrosis factor alpha in activating the mitogen‐activated protein kinase pathway and has been associated with neurodevelopmental disorders. *PACSIN3*, a neuronal protein, functions in actin regulation in vesicle formation in neurons. Similarly, *NR1H3/LXRA* is expressed in the liver and also in the brain, where it functions as a regulator of lipid homeostasis and inflammation.

In addition, 27 high confidence cardiometabolic trait (13 novel) associated variants showed chromatin–chromatin interaction evidence with TSS of several genes, in brain tissues (Table S7). For instance, *TCFL2* associated novel rs4506565 and rs10787471 showed evidence of interaction with TSS of the same gene, which plays a key role in regulating glucose tolerance and is a known risk for type 2 diabetes. Also, rs180365 (*LINC02702‐BUD13*), rs964184 (*ZPR1*), rs662799 (*ZPR1*), rs10750097 (novel *APOA5*), rs6589574 (*APOA1*), rs562179 (novel *SIK3*), associated with lipid‐related traits and/or metabolic syndrome, showed evidence of interaction with TSS of *APOA1. APOA1* functions in cholesterol transport both in the brain and periphery.

Top HDL‐associated loci rs12446515 in intergenic *HERPUD1‐CETP* variants and several others in the same locus, for example rs9989419, rs193695, rs72786786, rs12448528, and rs12923459, might interact with TSS of *SLC12A3. SLC12A3* acts in neuronal membrane excitability and neurotransmission. Also, HDL‐associated rs12482560 (intergenic *LINC00310‐KCNE2*) could interact with *MRPS6*. *MRPS6* modulates glucose‐stimulated insulin secretion and *KCNE2* encodes a subunit of potassium voltage‐gated channels.

In addition to interactions with neuronal genes, some cardiometabolic hits exhibited looping evidence with TSS of genes important for cardiometabolic health like *APOA1, APOA4, APO5, APOC2, APOC3*, *GCKR*, *ZPR1*, and *EEPD1* in HepG2 cell lines (Table S8). This suggests that they may play regulatory roles in metabolic processes. Moreover, interaction of the neuronally functional genes with lipid/glycemic variants at the chromatin level to influence transcription plausibly underscores the significance of cardiometabolic‐related genetic variants in maintaining both cardiometabolic and cognitive health.

### 3.5 Gene set enrichment analysis expounded relevant cognitive and metabolic pathways

Gene set enrichment for overall cognitive functioning showed gene enrichment of *APP* and *NEDD4* along synaptic pathways, especially in pre‐ and postsynaptic processes (Table S13). Most of the cognition‐associated genes show high expression in the brain (Figure S6 in supporting information). Genes associated with executive functioning like *APP* show enrichment in presynaptic processes and assembly, whereas language‐associated *NEDD4* functions in catabolic postsynaptic processes (Table S13).

The lipid‐associated genes (subgenome‐wide from gene‐based tests on HDL‐C, LDL‐C, triglycerides, and TC) are enriched in cholesterol metabolism, glycerolipid metabolism, chylomicron and plasma lipoprotein remodeling, and so forth (Table S13). Several of them show high expression in the brain, adipose tissue, and the liver (Figures S7–S9 in supporting information). Genes associated with lipids, insulin resistance, and visceral adiposity showed enrichment not only in metabolic pathways, but also in pre‐ and postsynaptic processes, regulation of calcium levels, synaptic transmission, amyloid fiber formation, and AD, among others (Table S13), reflecting the complex interplay between cardiometabolic health and biological features underlying cognitive function in the general population.

### 3.6 MR elucidated causal links of cardiometabolic risk factors for cognition

Before testing for causal interrelationships between cardiometabolic risks and cognition phenotypes, genetic correlation analysis was performed. Genetic correlation indicates whether traits share some genetic influence and causality suggests one trait directly influences the development of another trait through a shared genetic mechanism. Thus, correlation does not necessarily imply causation. Nonetheless, positive genetic correlations were observed for traits within each cognition domain and within cardiometabolic traits, and a few negative correlations between cardiometabolic and cognitive traits (Figure 2B).

The Bonferroni‐corrected genome‐wide significant (*P* < 1.67 × 10^−9^) variants (highlighted with “*” in Table 1) associated with the four cardiometabolic traits, namely HDL‐C, triglycerides, TG:HDL, and VAI were used as instruments to infer causal connections between these cardiometabolic tests and cognitive tests. Significant adverse causal roles of the four cardiovascular risk factors were uncovered upon many of the cognitive measures (Table 2). In all these causality assessments reported below, strengths of genetic instruments were substantially high (*F* statistic > 10). Sensitivity analyses including strength of the instruments and Steiger test results showed that instruments were associated with exposure and not outcome. There was no horizontal pleiotropy as the intercept remained non‐significant. Additionally, no significant result was obtained upon considering constituent variants in significant genes from gene‐based tests as instrumental variables.

**FIGURE 3:**
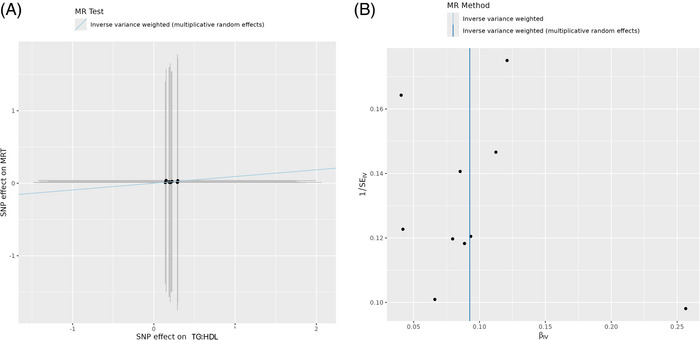
Adverse causal effect of high TG:HDL indicative of high insulin resistance on mean reaction time (attention domain). A, SNP exposure–SNP outcome association. Slope is indicative of positive causal effect where increase in TG:HDL increases mean reaction time, or in other words, adversely affects attention domain. B, Funnel plot showing no heterogeneity of effect estimates between the instrumental variables. HDL, high‐density lipoprotein; MR, Mendelian randomization; SNP, single nucleotide polymorphism; TG, triglyceride.

The study found adverse causal effects of low HDL‐C on memory (name–face associations; *β* = 0.127, SE = 0.035, *P* = 3.04E‐04, *F* = 66.05; Figure S10 in supporting information), visuospatial abilities (geometric figure task; *β* = 0.126, SE = 0.032, *P* = 7.47E‐05, *F* = 66.05; Figure S11 in supporting information); adverse effects of high triglycerides (*β* = –0.084, SE = 0.016, *P* = 1.86E‐07, *F* = 83.15; Figure S12 in supporting information), TG:HDL (*β* = –0.078, SE = 0.019, *P* = 5.60E‐05, *F* = 81.94; Figure S13 in supporting information), and VAI (*β* = –0.079, SE = 0.019, *P* = 4.99E‐05, *F* = 87.05; Figure S14 in supporting information) on name–face associations assessing associative memory. Additionally, adverse effects of high triglycerides (*β* = 0.075, SE = 0.013, *P* = 9.76E‐09, *F* = 83.15; Figure S15 in supporting information), high VAI (*β* = 0.084, SE = 0.010, *P* = 4.28E‐17, *F* = 87.05; Figure S16 in supporting information), and high TG:HDL (*β* = 0.093, SE = 0.015, *P* = 3.35E‐10, *F* = 81.94) on reaction time (Figure 3) were uncovered.

Figure 3A shows the SNP‐exposure and SNP–outcome associations for causal association of TG:HDL on mean reaction time. The funnel plot (Figure 3B) depicts no heterogeneity of effect estimates between the instrumental variables. The analysis for reverse causality did not yield significant results. No association implied confounding from heterogeneity (Table S14) or horizontal pleiotropy (Table S15). The Steiger directionality test for all causal associations depicted true causal direction of cardiometabolic traits on the cognition (Table S16). Apart from the above‐stated causal associations, protective causal associations of metabolic traits on cognitive functioning (Table 2) were also observed. These findings are encouraging and warrant further functional investigation and highlight the importance of metabolic well‐being for cognitive health.

### 3.7 Distinct haplotype structure in the Indian population associated with memory and metabolic syndrome

**FIGURE 4:**
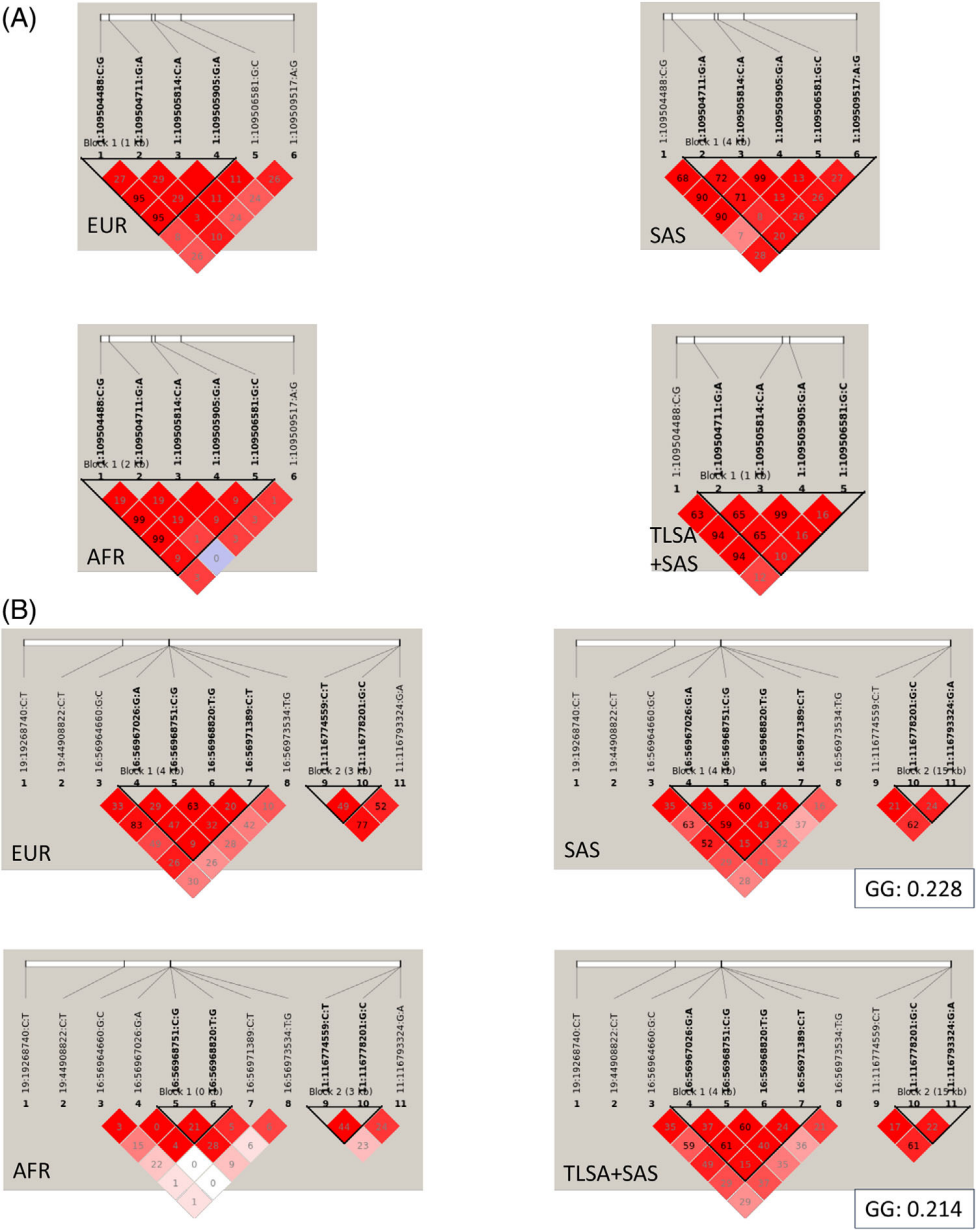
Distinct haplotype associations. A, Haplotype blocks constituting variants in *AMIGO1*. B, Haplotype blocks constituting variants in *TM6SF2*, *APOE, CETP*, *ZPR1*, and *APOA5*. The black outlined triangle depicts a haplotype block. The constituent variants in each block are also provided. The red triangles are shaded with lighter shades indicating lower LD and vice versa. The numbers in each triangle represents the pairwise LD between each of the variants. LD, linkage disequilibrium.

One of the previous studies identified missense rs146766120 in *AMIGO1* associated with episodic memory. In this study, *AMIGO1* was identified to be associated with delayed recall at a subgenome level of significance, upon collapsing coding variants (Table S11). These haplotypes with constituent burden‐accumulating independent variants in *AMIGO1*, were tested for association with delayed recall. Because haplotype association is usually performed in a case–control set‐up, 1581 individuals giving < 30% correct answers in the delayed recall test were designated as “cases” and 2436 individuals giving ≥ 30% correct answers in the delayed recall test were designated as “controls.” A haplotype AAAG formed by rs1009437 (G/A), rs3738772 (C/A), rs3738773 (G/A), and rs3738774 (G/C) was identified to be significantly associated with delayed recall functioning (Table 3). Moreover, this haplotype block differs in structure (constituent variants) from 1000G African, European, and SAS superpopulations (Figure 4A).

Because metabolic syndrome is a binary variable, haplotype association analysis was also conducted for this phenotype. Haplotypes constitute independent variants associated with any cardiometabolic traits under consideration at genome‐wide level of significance, in *TM6SF2*, *APOE*, *CETP*, *ZPR1*, and *APOA5*. These genes were prioritized because these were genome‐wide significant in gene‐based tests (collapsing PTVs) and were implicated in cardiometabolic traits. The haplotype blocks thus formed comprised variants in non‐coding genome. A significant association of GG haplotype, formed by the top TG:HDL‐associated rs964184 (G/C, 3′UTR *ZPR1*) and novel rs10750097 (G/A, upstream *APOA5*; Table 3) was uncovered. Here again, this haplotype block differs in structure from that in 1000G African and European superpopulations but matches 1000G SAS. However, it has ≈ 7% lower frequency in this study (0.214) compared to 1000G SAS (0.228), suggesting differential genetic susceptibilities for the diverse Indian population to metabolic syndrome, since the ethnic groups differ between 1000G SAS and this study (Figure 4B).

## 4 DISCUSSION

This study examined the genetic architecture of human cognition and cardiometabolic traits in a rural, aging, socioeconomically disadvantaged, less educated, health disparate cohort in India. The aim was to uncover causal relationships between these phenotypes to explain the epidemiological connection between cognitive impairment and cardiometabolic risks.

This study constructed an ancestry‐matched imputation panel with ∼1300 Indian whole genome sequences, identifying several novel associations with cognitive and cardiometabolic phenotypes. Accurate genotype imputation, crucial for GWASs, requires a large ancestry‐matched reference panel. CBR‐SANSCOG and CBR‐TLSA participants have closely matched genetic ancestry. To our knowledge, this is the first India‐specific imputation panel enriched with ancestrally south Indian haplotypes, offering more accurate imputation than other panels. Subsequently genotype–phenotype associations with imputed genotypes uncovered population‐specific effects of genetic variants on these traits.

This study identified several high‐confidence variants associated with cognitive functioning, previously unidentified in large‐scale genome‐wide studies on cognition/intelligence. The genome‐wide significant novel intergenic *FLRT2*‐*LINC02328* (rs12588896) was associated with recognition memory. *FLRT2* influences cell adhesion, cortical neuron migration, and axon guidance through interactions with G‐protein coupled receptors (GPCRs) and *UNC*‐5 receptors. This study also highlighted subgenome‐wide significant variants with high deleteriousness mapped to genes with neuronal functions. The *UNC5C* variant (rs6822806, CADD 18.33), is highly deleterious, associated with naming association (memory), and has been previously associated with major depressive disorder.^48^ Several *UNC5C* variants have also been associated with brain structural phenotypes and AD pathogenesis.^49^ HMSE associated *DAOA* and visuospatial span associated *DOCK9* have been linked to bipolar disorder and schizophrenia.^50^, ^51^ Similarly, *NLRP3* inflammosomes have been implicated in cognitive impairment,^52^ whereas *PDGFC* signaling has been previously known to ameliorate neuroinflammation by blood–brain barrier disruption in mouse models.^53^ All these genes have neurodevelopmental or neurodegenerative functions.

Gene‐based tests identified *H2BC14*, *ZNF500*, *MELTF*, and *MVB12B* as associated with attention, visuospatial abilities, and executive functioning at subgenome‐wide significance. *H2BC14* encodes a histone protein; dysregulation of histone cluster genes can alter genome structure and gene transcription. Additional subgenome‐wide significant (*P* < 10^−5^) cognition‐associated genes included *AMIGO1*, *APP, KCTD20*, and *NEDD4*. *APP* is key to amyloid beta (Aβ) formation and AD pathogenesis. *AMIGO1*, a neuronal cell‐adhesion protein affecting neurite growth, fasciculation, and myelination is also associated with schizophrenia. To the best of our knowledge, this is the first Indian study to report *APP, AMIGO1* gene‐based associations at the population level. This study also highlighted a novel haplotype AAAG in *AMIGO1* associated with delayed recall in the Indian population. This haplotype comprised rs1009437, rs3738772, rs3738773, and rs3738774; rs1009437 and rs3738774 are novel, whereas rs3738772 and rs3738773 have reported associations with intelligence and education.^54^ The *KCTD* family regulates GPCRs and is linked to neuropsychiatric and neurogenerative disorders.^55^ *NEDD4*, a ubiquitin ligase, is critical for neuronal development and oxidative stress regulation via insulin growth factor in neurons.^56^ Overall, these findings emphasize the role of GPCRs and cell adhesion molecules in cognition (memory and attention), probably via neural development, synaptic formation, synaptic plasticity, and neurotransmission.

This study identified two genome‐wide significant genes associated with cardiometabolic phenotypes—*TM6SF2* and *CFTR*—passing the phenotype‐level Bonferroni correction. *TM6SF2* is the well‐known type 2 diabetes and NAFLD risk gene. NAFLD has been linked to poor cognitive function and reduced brain volume, sometimes independent of cardiometabolic risk, emphasizing genetic predisposition‐based risk stratification of individuals.^57^ Though not classically linked to cognition, emerging evidence implicates them in cognitive impairment through underlying roles in NAFLD and cystic fibrosis. This study also highlighted several novel and well‐established high‐confidence genetic variants and genes associated with cardiometabolic traits. For example, *CETP* and *APOE* facilitate cholesterol metabolism, and function in shared pathways of late‐onset AD and cognitive decline during aging.^58^ *BACE1*, implicated in AD pathogenesis, through APP cleavage and Aβ formation, was also associated with triglycerides, TG:HDL, and VAI, suggesting lipid levels may influence its activity and contribute to mechanisms of cognitive decline.

This study uncovered known missense variants like rs5104 (Ser → Asn) in *APOA4*, rs5882 (Val → Ile) in *CETP*, and rs1260326 (Leu → Pro) in *GCKR* associated with cardiometabolic phenotypes. *GCKR* encodes a hepatocyte‐specific inhibitor of the glucose‐metabolizing enzyme regulating glucose uptake, storage, and release. The study highlighted *ZPR1*, with its most significant variant, rs964184, associated with triglycerides, TG:HDL, and VAI. Located near the *APOA5*–*APOA4*–*APOC3*–*APOA1* locus, this UTR variant encodes zinc finger protein 1—involved in neuronal cell death via genomic stability disruption. A high‐fat diet can cause *ZPR1* downregulation‐mediated hippocampal neuron loss.^59^ Several relevant transcription factors (TFs) bind to the promoter region of *ZPR1*, including *PPARG* (functioning in insulin sensitivity and obesity) and *HNF4A* (triggering genes involved in glucose, fatty acid, and cholesterol metabolism).^60^ This study provided the first evidence of PTVs in *GLP1R*, a GPCR functioning in insulin regulation, associated with visceral adiposity in the Indian population. Future well‐powered studies can explore their role in insulin resistance and cognitive decline.^60^ Interactions between rs964184(*ZPR1*) and functional polymorphisms in *APOA5* may influence lipid metabolism. The *APOA1–APOC3–APOA4–APOA5–ZPR1–BUD13–SIK3* cluster has been implicated in lipid metabolism and neuronal processes, potentially affecting cognition and neurodegenerative diseases.^61^, ^62^, ^63^

This work also hinted at orthogonal support for the regulatory roles of non‐coding variants linking cardiometabolic traits and cognitive function through shared genetic and molecular pathways. The variant rs583104, associated with total cholesterol and located downstream of *CELSR2*, is an eQTL for *PSRC1* expression in the frontal cortex (BA9), and showed strong colocalization (PP > 99.99%) with *PSRC1* eQTLs in the putamen, cortex, and adipose tissue. The *CELSR2–PSRC1–SORT1* locus, known for cardiovascular roles, has also been implicated in cognition, with large‐scale GWAS mapping cognition‐associated variants to gene sets including these genes (https://genetics.opentargets.org/study‐comparison/GCST005142). Colocalization of cardiometabolic trait variants with brain and adipose eQTLs, along with epidemiological evidence, highlights metabolic pathways influencing cognition and suggests possible causal links. Some cognition‐associated variants overlap with chromatin loops, suggesting interactions with neuronal genes, while cardiometabolic hits may interact with genes involved in both metabolic and neuronal processes. These findings suggest spatio‐transcriptional roles of these variants in cognition, reflecting a complex interplay between brain regulation and systemic sugar–lipid metabolism. This underscores how multi‐gene interactions across tissues shape complex traits through networks involved in metabolic homeostasis.

This study presented evidence of cardiometabolic traits influencing cognition through genetic instruments in genes like *APOC3–APOA4–APOA5–ZPR1–BUD13*, among others. These genes function in metabolism, inflammation, or lipid regulation, potentially mediating the relationship between exposures and cognitive functioning, though the exact mechanism is unclear. However, causal estimates should be interpreted considering underlying assumptions, as residual confounding and unaccounted pleiotropy, difficult to detect in real human populations, may affect the results. Adverse effects of TG:HDL, triglycerides, and VAI were observed on mean reaction time and name–face associations, related to attention and memory domains, with the hippocampus playing a key role in their interplay.^64^ Additionally, the negative impact of low HDL‐C on the visuospatial domain was also uncovered. This work thus highlights the complex causal liaison between systemic lipid, glucose metabolism, insulin resistance, and brain function, affecting cognition, with contributions from known and novel loci in the Indian population. Multiple lines of evidence—including single‐variant tests, gene‐based tests, spatial overlap with chromatin loops, eQTL colocalization, MR, and haplotype‐based analysis—suggests the *ZPR1–APOA5* loci as a key candidate with notable effects on cardiometabolic traits and cognitive phenotypes. Collectively, these findings emphasize this gene locus as a key candidate for tracking, monitoring, or risk‐stratifying and metabolic profiling individuals vulnerable to cognitive impairment.

To our knowledge, this study is the first to identify a novel GG haplotype (*ZPR1* rs964184 and novel *APOA5* rs10750097) associated with metabolic syndrome in the Indian population, distinct from other populations. These variants were associated with the insulin resistance biomarker‐ TG:HDL, triglycerides, VAI, and HDL‐C.

This study demonstrated the importance of capturing genetic susceptibility in underrepresented populations. However, the small sample size limited power to detect many genome‐wide signals, with some of the top hits not surpassing phenotype‐level multiple testing correction thresholds. As a first of its kind study in rural Indian populations, findings and effect estimates remain to be seen in larger populations with similar genetic and environmental backgrounds with harmonized phenotypes. Despite detailed quality checks and functional genomics support, residual confounding may still exist, which might impact downstream MR results. Thus, MR estimates should be interpreted cautiously as the assumption that genetic variants affect outcomes only through exposure may not always hold, which can be said with more certainty in larger future studies. The results can be generalizable as known cardiometabolic loci are replicated—supporting broader relevance. However, caution is necessary when extrapolating findings to other populations. Larger studies and meta‐analyses are needed to refine effect estimates and inform public health strategies with population‐specific data.

Taken together, these results pinpoint several new loci linked to cognition, and reveal the causal role of cardiometabolic risks for cognition, addressing gaps in studies on the understudied Indian population, many of whom live in villages. These findings are encouraging, and reiterate emerging relationship between cardiometabolic health and cognition, entailing further research into the causal mechanisms involved. Understanding these genetic influences can uncover mechanisms behind cognitive dysfunction, especially in populations disproportionately affected by dyslipidemia and insulin resistance. This knowledge can guide public health strategies targeting diabetes, hypertension, and hypercholesterolemia, ultimately improving cognitive outcomes in health‐disparate populations.

## CONFLICT OF INTEREST STATEMENT

The authors declare no competing interests. Author disclosures are available in the supporting information.

## CONSENT STATEMENT

All human subjects involved in this study provided informed consent prior to participation.

## Supporting information



Supporting information

Supporting information

Supporting information

Supporting information
