# Supplementary material for: Genome‐wide study links cardiometabolic factors to cognition via APOA4‐APOA5‐ZPR1‐BUD13 and other loci in rural Indians
Source: Alzheimers Dement. 2025 Jul 15;21(7):e70429. doi: 10.1002/alz.70429 (PMC12263347; doi:10.1002/alz.70429)
Supplement: Supplementary file 2 — Supporting information [file ALZ-21-e70429-s002.docx]

**Supplemental Information:**

**Phenotypes analysed:**

1. Cognition-related (20 phenotypes tested):
   - Hindi Mental Status Examination (1): This provides a screening tool primarily targeted at illiterate demented patients for assessing if an individual is cognitively healthy or not.

- COGNITO - Attention (4):
  - - Auditory attention, visual attention, dual attention, mean reaction time
- COGNITO -Memory (4)
  - - Delayed recall, Immediate recall, name recognition, name face associations (face & name)
- COGNITO -Language (5)
  - - Comprehension, reading, vocabulary, phonetic, semantic
- COGNITO -Visuospatial abilities (3)
  - - Visuospatial span, geometric figures, Stroop test- description
- Executive functioning (2)
  - - Naming associations/semantic associations (part of COGNITO), and TMT_B-A(Trail making test(TMT) B – TMT(A))

Phenotypes such as TMTB-A and mean reaction time provide measurements in the unit of time. Rest of the cognitive phenotypes are assessed by the proportion of correct answers in the test battery results. A full description of the COGNITO measures can be found in Kahali et al., 2023^1^ and Ritchie et. al 2014^2^

- G-factor (1): 15 component tests assessed by COGNITO which have data-reported for atleast 2000 individuals are considered for this phenotype generation. These tests span across attention (1/mean reaction time, auditory attention, visual attention), memory (delayed recall, immediate recall, name recognition, name -face associations (name &face), language (reading, comprehension, phonetic, semantic), executive functioning (naming associations/semantic associations) and visuospatial (span, geometric figures) domains. A factor analysis with two factors is conducted on these 15 phenotypes and the first factor is considered as the “g-factor” ^3^: a composite score assessing general cognitive functioning. All these constituent scores are positively correlated with each other.

Attention is evaluated through both hearing and visual tasks. The auditory task asks participants to differentiate between different sounds, while the visual task requires them to pick out a specific image from a group of distractors. There's also a combined task, where participants need to do both the auditory and visual tasks at the same time. Language skills are assessed using a variety of exercises, including reading comprehension, recognizing sounds (phonemes), naming objects, making associations between words, fluency in speaking, and testing vocabulary. These tasks are carefully designed to cover different aspects of language, such as how we understand and use sounds (phonology), word structure (morphology), sentence structure (syntax), and meaning (semantics).

For memory assessment, participants are asked to recall a list of names both immediately and after a delay. There's also a task where they need to remember names and faces.Visuo-spatial processing is tested through tasks like building something (construction task), solving patterns or puzzles (matrix reasoning task), and matching objects based on their meaning or function. These tasks help evaluate how well individuals process and understand visual and spatial information. The COGNITO-mean reaction time and TMT-B-A are assessed in units of time (seconds). For the other COGNITO phenotypes proportion of correct answers (unit-free) in out of the times a cognitively discriminatory task is administered, is selected as the phenotype of interest.

Cardiometabolic (10 phenotypes tested):

- - HDL-C (mg/dL)
  - LDL-C (mg/dL)
  - Triglycerides (mg/dL)
  - Total cholesterol (mg/dL)
  - Fasting blood sugar (mg/dL)
  - HbA1c (%)
  - Visceral fat (%)
  - Visceral adiposity index
  - TG-HDL ratio
  - Metabolic syndrome: NCEP-ATP III^4^ criteria has been used to assign metabolic syndrome cases. Individuals having 3 or more of the below 5 criteria have been assigned as metabolic syndrome cases.
- Fasting glucose ≥ 100 mg/dl
- Systolic BP ≥130 mmHg and/or diastolic BP ≥ 85 mmHg
- Triglycerides ≥ 150 mg/dl
- HDL cholesterol <40 mg/dl for men and < 50 mg/dl for women
- Waist circumference of ≥102 cm in men or ≥88 cm in women

**Whole genome sequencing analysis pipeline:**

Whole-genome sequencing data of 696 "CBR-TLSA” individuals is assessed, upon approval from the Institutional Human Ethics Committees of the Indian Institute of Science and Centre for Brain Research (Ref. no. CBR/42/IEC/2022-23). The Field Data Collector (FDC) team organize awareness campaigns and recruit participants for these studies through phone calls or in-person home visits. All participants provide written informed consent. Then, genomic DNA extraction is conducted from peripheral blood samples and Illumina NovaSeq 6000 libraries are prepared from these study individuals to perform whole genome sequencing on the Illumina NovaSeq 6000 platform (Illumina Inc., San Diego, CA, USA) using the NovaSeq 6000 S4 reagent kit (Illumina, Cat. No. 20028312). The raw sequencer data in CBCL format is considered for whole genome sequencing analysis. WGS analysis is carrid out using the following steps: Conversion od raw image base call files to an unaligned bam (.ubam) file using GATK ExtractIlluminaBarcodes and GATK IlluminaBasecallsToSam modules ^5^. This process, called demultiplexing, generates lane-wise unaligned BAM files. All 4-lane unaligned BAM files for each sample are then merged into one unaligned BAM file for subsequent steps by looping on the number of samples. An unaligned bam file is sorted by query name using sambamba ^6^ and a sequence alignment mapping (SAM) file is created to restore the original quality score and other defined properties using GATK RevertedSAM. The 5' start position of a specified adapter sequence and the 3' end position of specified read adapters are marked using the GATK MarkilluminaAdapter module. This SAM file is then converted into paired-end (forward and reverse) FASTQ format using the GATK SamToFastq module for parsing input for alignment. Next, each sample of paired-end FASTQ files is aligned against the current GRCh38 human reference build to generate SAM format using BWA MEM ^7^. Further, SAM files are split into 22 autosomes and X, Y, and M chromosome SAM files in zipped (.gz) format using an in-house Perl script. This step requires more memory because it has to iterate over the number of samples and the number of chromosomes. After converting chromosome-wise SAM files into binary alignment files (.bam), these files are sorted based on coordinates using SAMtools ^8^ and duplicate reads are marked. Downstream GATK modules will ignore these duplicates marked as read by default by internally applying a read filter. The base qualities are recalibrated more accurately using the GATK-BaseRecalibrator module, which improves the accuracy of the variant calls. Those recalibrated base qualities from the recalibration table are applied to the aligned data using GATK-ApplyBQSR. The goal of this procedure is to correct for systematic bias that affects the assignment of base quality scores by the sequencer. Thus, individual-level variants are generated by calling germline genetic variants using the GATK-Haplotypecaller module. The GATK-GenomicsDBImport module is then processed for storing up to hundreds of thousands of unique genome variant data of multiple individuals. Finally, joint genotyping variant calling format (.vcf) file of multi-sample is performed using GATK-GenotypeGVCFs. Additionally, GATK-VariantAnnotator is utilized to annotate the call set. Afterward, quality assessments are conducted on the raw variants using the variant recalibration model with GATK-VariantRecalibrator. This process helps identify the "true sites" within the cohort by comparing them against a dataset that includes HapMap3 sites, dbSNPs, InDELs, Omni 2.5M SNP, and 1000 Genome variant sites known to be polymorphic. Finally, the population level VCF file of 696 individuals is generated using GATK-ApplyVQSR for all chromosomes including Chr 1 to 22, X, Y and M.


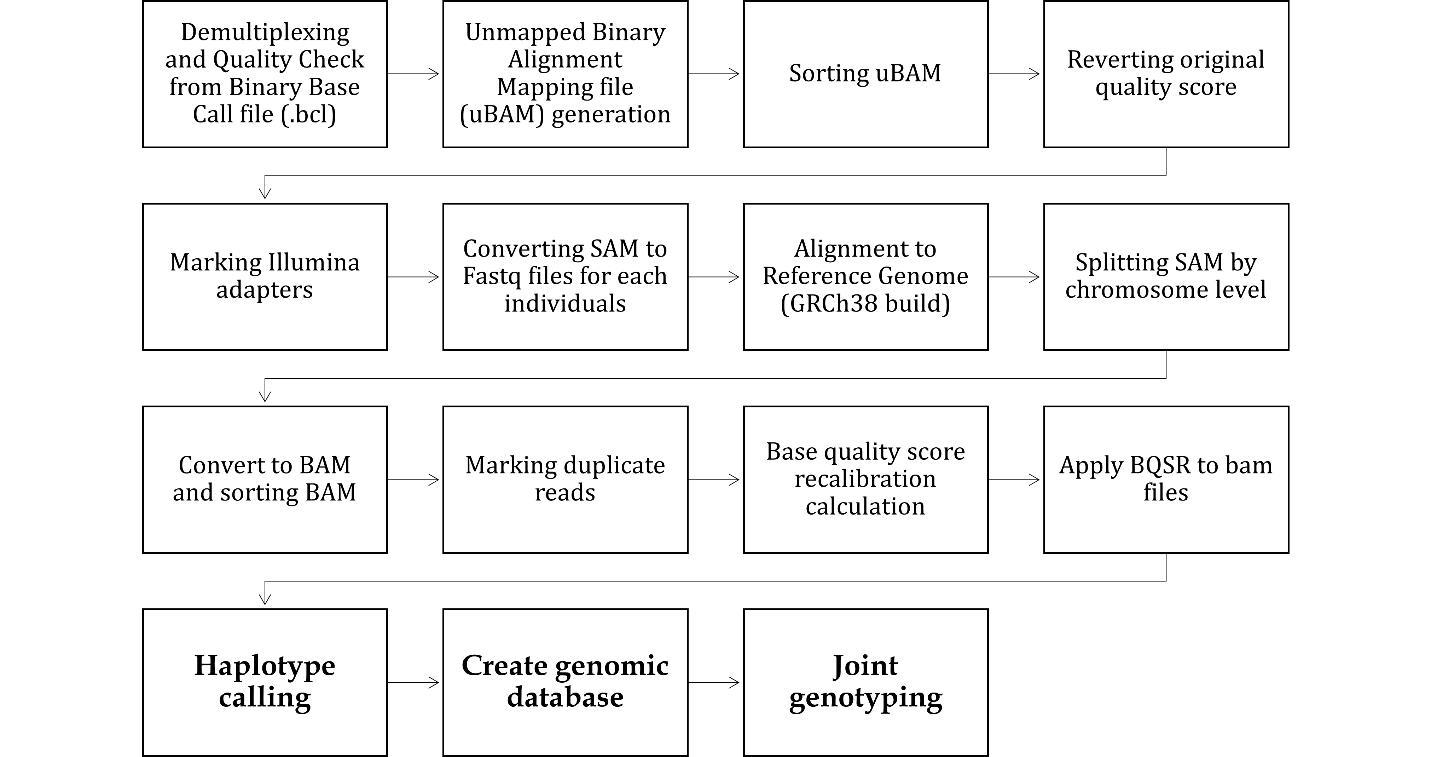


**Supplementary note figure 1: Workflow for variant calling in Human Whole Genome Sequencing (WGS)**

**Legend:** This figure presents the step-by-step workflow for analyzing human whole genome sequencing (WGS) data and calling genetic variants SNV and INDEL in 696 individual samples. Starting from sequencing raw .bcl files to population-level joint genotyped VCF file.

**Batch Effect Mitigation:**
To minimize batch effects, all 696 WGS samples have been sequenced on the NovaSeq 6000 platform using a consistent protocol for DNA isolation, library preparation and sequencing. Each sequencing run included either 24 samples per lane or 48 samples across two lanes, ensuring uniform experimental conditions across the dataset.

**Benchmarking with GIAB samples:**

We also perform benchmarking of four Genome in a Bottle (GIAB) samples. The Genome in a Bottle (GIAB) consortium provides a high-confidence truth set of single nucleotide variants (SNVs), small insertions, and deletions (InDels) for the samples NA12878, NA24149, NA24143, and NA24385. The WGS pipeline used to create the panel, achieves ~ 99% precision and ~97% recall, ensuring robust variant detection across the cohort-level analysis.

**Individual-level QC**

Before performing joint genotyping on the 696 TLSA individuals, we conduct comprehensive quality control assessments to ensure the reliability of the variant data. WWe observe that the average sequencing depth across variants per individual is approximately 23, with a standard deviation of ±12. The mean genotype quality (GQ) per individual is around 30, with a standard deviation of ±11.5, indicating a generally high confidence in the genotype calls. Additionally, we identify an average of approximately 7.5 million genetic variants per individual, with minimal deviation in variant counts between individuals, reflecting consistent and comprehensive genome-wide variant detection.

**Joint Genotyping Approach:**
We performed joint genotyping of all 696 samples using the GATK-genotypeGVCFs module, a widely accepted approach for joint calling of genetic variants at single nucleotide and small insertions and deletions level. This method ensures that all samples undergo variant detection in a unified manner, mitigating technical variations that could arise from processing samples separately.

**Quality Control Measures:**
In addition to following uniform experimental and analytical conditions to ensure consistency and reliability of resultant variant call set, we also conducted rigorous quality control (QC) checks at each stage of the WGS pipeline to assess potential batch effects. The distributions of key sequencing metrics across all samples showed minimal variability (some amount of variability is expected, but we also see that the X-axis ranges are minimal, thus this amount of variability is acceptable in real experimental conditions) (Figure 1 below same as Supplementary figure 2). This confirms the absence of significant batch effects:

- **Coverage distribution (Figure 1A):** The average sequencing depth was **42X**, with the majority of samples exceeding 30X coverage.
- **Phred quality score distribution (Figure 1B):** The mean Phred score across all samples was **36**, indicating high base-calling accuracy.
- **Read mapping percentage (Figure 1C):** Over **95% of reads** successfully mapped to the **GRCh38 reference genome**, ensuring consistency in alignment quality.
- **Paired-end read insert size distribution (Figure 1D):** Despite a read length of **2 × 150 bp**, a significant proportion of samples exhibited an insert size exceeding **400 bp**, demonstrating effective paired-end mapping. **8**
- **Duplicate rate:** The duplicate rate was around **6-%*.***

**Supplementary note figure 2:** Distribution of sequencing metrics in the jointly-called whole genome sequencing call set, demonstrating batch effects in control (see minimal x-axis ranges). The metrics are: A) Coverage distribution; B) Average Phred Quality score; C)Mapping percentage; D) Insert size distribution

These metrices collectively confirm the robustness of our sequencing and analysis pipeline, demonstrating that batch effects were effectively minimized.

**Imputation Process:**

The process of imputation is divided into two phases: i) phasing – to infer underlying haplotypes of the individuals ii) imputation- to integrate the inferred haplotypes with a reference panel of haplotypes and impute missing or unobserved genotypes in each sample.

Genotypes of 601 individuals with SAS ancestry are extracted from the high coverage illumina integrated phased panel which included single nucleotide variants (SNVs), insertions and deletions (INDELs), and structural variations (SV- (large deletions (DEL), insertions (INS), duplications (DUP), and inversions (INV)) variant calls across 3,202 1kGP samples. (Detailed methodology in <http://ftp.1000genomes.ebi.ac.uk/vol1/ftp/data_collections/1000G_2504_high_coverage/working/20220422_3202_phased_SNV_INDEL_SV/README_1kGP_phased_panel_110722.pdf>). Apart from the quality checks performed by 1000 genomes, non-variant sites for these 601 individuals (AC=0) are filtered out. Further, multiallelic sites are removed along with indels and structural variations with |Ref allele length -ALT allele length | >= 30 bases. Duplicate sites and monomorphic sites are also removed. As part of quality checks on Tata Longitudinal Study for Aging (CBR-TLSA cohort) WGS data, samples with sample missingness > 3% are removed. Here again non-variant sites, singletons and monomorphic sites are removed. Similarly biallelic variants are retained and sites for which allele length with>= 30 bases are removed. Sites with minor allele count <2, HWE p-value <10 ^-10^ are also removed. The sites that remain has QUAL>=30, GQ>=20.

Haplotype phasing is performed with SHAPEIT5^9^ in two stages. First,variants with MAF >0.1% are phase and these phase genotypes are used as a scaffold to obtain final phased haplotypes for TLSA panel.

The two panels are then merged – 1000 genomes SAS quality controlled WGS -based vcf for 601 individuals and quality controlled WGS-based vcf for 696 TLSA participants, using bcftools merge to create our own merged haplotype reference panel.

Prior to imputation, an indexed co-ordinate file defining start and end points of length 500000 bases that is to be considered for imputation is generated using imp5Chunker_1.1.5^10^ . Imputation is then performed for these defined regions using IMPUTE5^10^ to obtain imputed chunks which are then concatenated and sorted by chromosome and position to obtain the final imputed genotypes.

**Hi-C datasets used for investigating spatial overlap of chromatin loops with association signals:**

The following HiC datasets from GEO (Gene Expression Omnibus) have been used for the spatial overlap analysis for cognitive hits:

- GSE237345_ENCFF467KEZ_loops_GRCh38.bedpe
- GSE237712_ENCFF730RTY_loops_GRCh38.bedpe
- GSE238042_ENCFF216IAK_loops_GRCh38.bedpe

A brief description about the respective tissues and samples for which the Hi-C experiment was conducted is given below. Both the donors were recruited from Rush’s AD cohort.

| **GSE ID** | **DATASET** | **SPECIES** | **ENCODE DONOR ID** | **SEX OF DONOR** | **AGE OF DONOR** | **DONOR’S ANCESTRY** | **NO. OF SAMPLES** | **REGION OF INTEREST** | **FUNCTION OF REGION** |
| --- | --- | --- | --- | --- | --- | --- | --- | --- | --- |
| GSE238042 | ENCODE | Homo Sapiens | ENCDO707TUE | Female | 89 | European | 2; GSM7658001, GSM7658002 | Brain : Posterior cingulate gyrus | Top-down control of visual attention, eye movement, frontoparietal control network (including DMN) |
| GSE237712 | ENCODE | Homo Sapiens | ENCDO707TUE | Female | 89 | European | 2; GSM7645485, GSM7645486 | Brain : Caudate nucleus | Movement execution, executive function, learning, memory, reward , motivaton, emotion |
| GSE237345 | ENCODE | Homo Sapiens | ENCDO623FPG | Male | 78 | European | 2; GSM7610354, GSM7610343 | Brain : Dorso-lateral prefrontal cortex | Task -switching, task-set recognition, planning and working memory |

For the lipid traits, the following GEO ID have been used:

GEO ID - GSM5014501 (File: GSM5014501_HepG2_washU.loops.hg38.txt)

Cell line – Hep G2

Build -(GRCh37) [ Python module pyliftover used to lift the coordinates to GRCh38 ]

Species- Homo Sapiens

The schema of the analysis is given below:


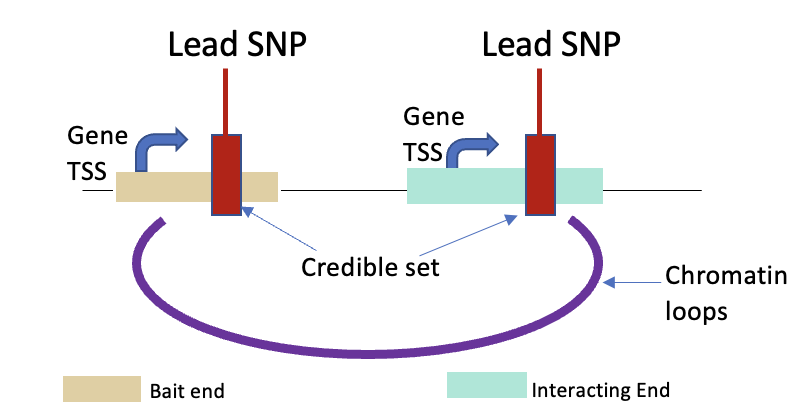


Here the genomic coordinates of “clumped-set-for-plausible-interactions” variants tagged by the lead SNPs is spatially superimposed on the apriori known biological interactions, i.e loop coordinates, to explore possibilities of interactions with genes located at the opposite end by regulating their respective gene expression.

**Justification for using rvtest instead of REGENIE for the single variant association**

rvtests is better suited for rare variants, we use, linear mixed model- methods taking relatedness into consideration and not REGENIE for our single variant association. We have calculated the genetic relatedness matrix based on all samples in rvtests. We are using a minor allele count (MAC) cutoff of 2 and subsequently, a post-imputation minor allele frequency (MAF) cutoff at 0.02%, which is quite rare. REGENIE is a machine-learning based technique, better suited to common variants and the documentation states that REGENIE can get conservative in the event of relatedness and is not usually recommended for smaller cohorts (<https://rgcgithub.github.io/regenie/faq/>). In such a situation, akin to ours, exact mixed-model approaches fare better, and that is the reason why we have used RVTESTs instead of REGENIE,

Rare-variant association tests for combining effects from varaints at gene level can be broadly divided into two categories-burden tests and variance-component tests.

If $y_{i}=\mu_{i}+{}^{\boldsymbol{'}}\boldsymbol{G}_{\boldsymbol{i}}+{}_{i}$ is our model where $\boldsymbol{\beta}$ correspond to the effects of constituent variants in the gene-set for ith individual, then we are interested in testing H_0_: $\boldsymbol{\beta=0}$, i.e say *β_1_ = β_2_ = … = β_q_ = 0* ; q variants collapsed in a mask and tested as a set.

Burden tests aggregate genetic variants into a single score, assuming that they influence the phenotype in the same direction, which means

$\beta_{\boldsymbol{j}}={w_{\boldsymbol{j}}\beta}_{\boldsymbol{c}}$ and the test statistic is $Q_{B}=\left[ \sum_{i=1}^{n} \left( y_{i}-\hat{}_{i} \right)\left( \sum_{j=1}^{p} w_{\boldsymbol{j}}g_{\mathrm{ij}} \right) \right]^{2}$

Variance-component tests or kernel-based tests relax this assumption of a common direction of effect and instead presumes that effect sizes follow a distribution with mean zero and finite variance, that is

β_j_ ~(0,w_j_τ), τ variance component, *w_j_*  specified weight for variant j.

Then the revised hypothesis becomes H_0_*:* τ **= 0**  and the test statistic is

***Q_Κ_=*** ${(\mathbf{y-}\hat{}\mathbf{)}}^{'}\mathbf{K}(\mathbf{y-}\hat{})$ ; K= $\sum_{j=1}^{q} w_{j}G_{ij}G_{i'j}$ ;

The weight is often assigned as $w_{j}$***=*** Beta($\mathrm{MAF}_{j} ;a_{1}$=1 $a_{2}$=25) to put more weights on rarer variants. Kernel-based tests, such as SKAT, works better when some rare variants are non-causal or exhibit opposing effects.

Given that the true genetic architecture of a trait is often unknown, omnibus tests like SKAT-O (Optimal SKAT) adaptively combines the strengths of both burden and kernel tests . The test statistic in that case becomes

$Q={\rho Q}_{B}+\left( 1-\rho\right)Q_{K}$; 0$\leq$ρ $\leq1$1

Thus omnibus tests ensure robustness and higher power for rare-variant association testing, regardless of whether the underlying genetic model fits the assumptions of either tests.

Here we are testing coding variants and protein truncating variants (PTVs) which are mostly rare variants to analyse their combined effect on the trait of interest. The key reason for this choice is this: RVTEST provides burden or kernel-based test for related samples like fam-SKAT, but do not provide omnibus tests like SKATO, SKATO-ACAT for related samples. Instead REGENIE provides such tests while taking relatedness into account. SKATO-ACAT is an even more powerful method which maximizes power across SKATO models using Cauchy combination and the test statistic becomes

T=$\sum_{Q=\{Burden,Kernel,ACATV\}} \omega_{Q}tan\{\left( 0.5-p_{Q} \right)\pi\}$ where p is the p-value.

Thus, in an effort to optimize power and accuracy, we use RVTEST for our single variant association and REGENIE for our gene-based tests.

We have included a clarification in the main paper for enhanced readability in section 2.11 lines 292-297.

**ETHICS STATEMENT:**

Ethical clearance for the CBR-SANSCOG and CBR-TLSA studies were obtained from the Institutional Human Ethics Committee at the Centre for Brain Research (Ref. no. CBR/42/IEC/2023-24 and Ref. no. CBR/42/IEC/2022-23) respectively.

**AUTHOR CONTRIBUTIONS**

B.K. conceived and designed the study. SANSCOG team, J.S, collected phenotypic data. A.M, T.G.I and TLSA team collected blood samples for WGS. K.V.R performed the WGS. K.S and B.K performed TLSA-WGS analysis. A.R, S.C created merged reference panel and performed imputation, R.M and S.C performed multimodal annotation. S.C and B.K performed all other analysis. S.C. and B.K. wrote the manuscript and prepared the figures and tables. All authors have read and approved the final manuscript.

**DATA AND CODE AVAILABILITY**

The data reported in this paper will be shared by the lead contact upon request. Custom codes for analysis are available publicly on Github: <https://github.com/BratatiKahaliLab/Genetic-architecture-of-cardiometabolic-traits-and-cognition-in-the-Indian-population.git>. Any additional information required to reanalyze the data reported in this paper is available from the lead contact upon request.

**Reference:**

1. Bratati Kahali *et al.* COGNITO (Computerized assessment of adult information processing): Normative scores for rural Indian population from SANSCOG study. *Alzheimers Dement* (2022).

2. de Roquefeuil Guilhem, R. K. COGNITO: Computerized Assessment of Information Processing. *J Psychol Psychother* **04**, (2014).

3. Jensen, A. R. The *g* Factor: Psychometrics and Biology. in 37–57 (2000). doi:10.1002/0470870850.ch3.

4. Sundarakumar, J. S., Stezin, A., Menesgere, A. L., Ravindranath, V. & SANSCOG and TLSA Collaborators. Rural-urban and gender differences in metabolic syndrome in the aging population from southern India: Two parallel, prospective cohort studies. *EClinicalMedicine* **47**, 101395 (2022).

5. McKenna, A. *et al.* The genome analysis toolkit: A MapReduce framework for analyzing next-generation DNA sequencing data. *Genome Res* **20**, (2010).

6. Tarasov, A., Vilella, A. J., Cuppen, E., Nijman, I. J. & Prins, P. Sambamba: Fast processing of NGS alignment formats. *Bioinformatics* **31**, (2015).

7. Li, H. & Durbin, R. Fast and accurate short read alignment with Burrows-Wheeler transform. *Bioinformatics* **25**, (2009).

8. Li, H. *et al.* The Sequence Alignment/Map format and SAMtools. *Bioinformatics* **25**, (2009).

9. Hofmeister, R. J., Ribeiro, D. M., Rubinacci, S. & Delaneau, O. Accurate rare variant phasing of whole-genome and whole-exome sequencing data in the UK Biobank. *Nat Genet* **55**, 1243–1249 (2023).

10. Rubinacci, S., Delaneau, O. & Marchini, J. Genotype imputation using the Positional Burrows Wheeler Transform. *PLoS Genet* **16**, e1009049 (2020).

Figure S1- Our workflow:

Genotyping individuals with array data, imputation with a new ancestry-matched imputation panel, association analysis, multimodal functional annotation, following up with mendelian randomization to assess causal links between cardiometabolic and cognition and finally comparing haplotype structure with variants associated with these traits across other global-ancestry cohorts.

Figure S2-Overlap between genome-wide significant lipid and insulin-resistance hits

**
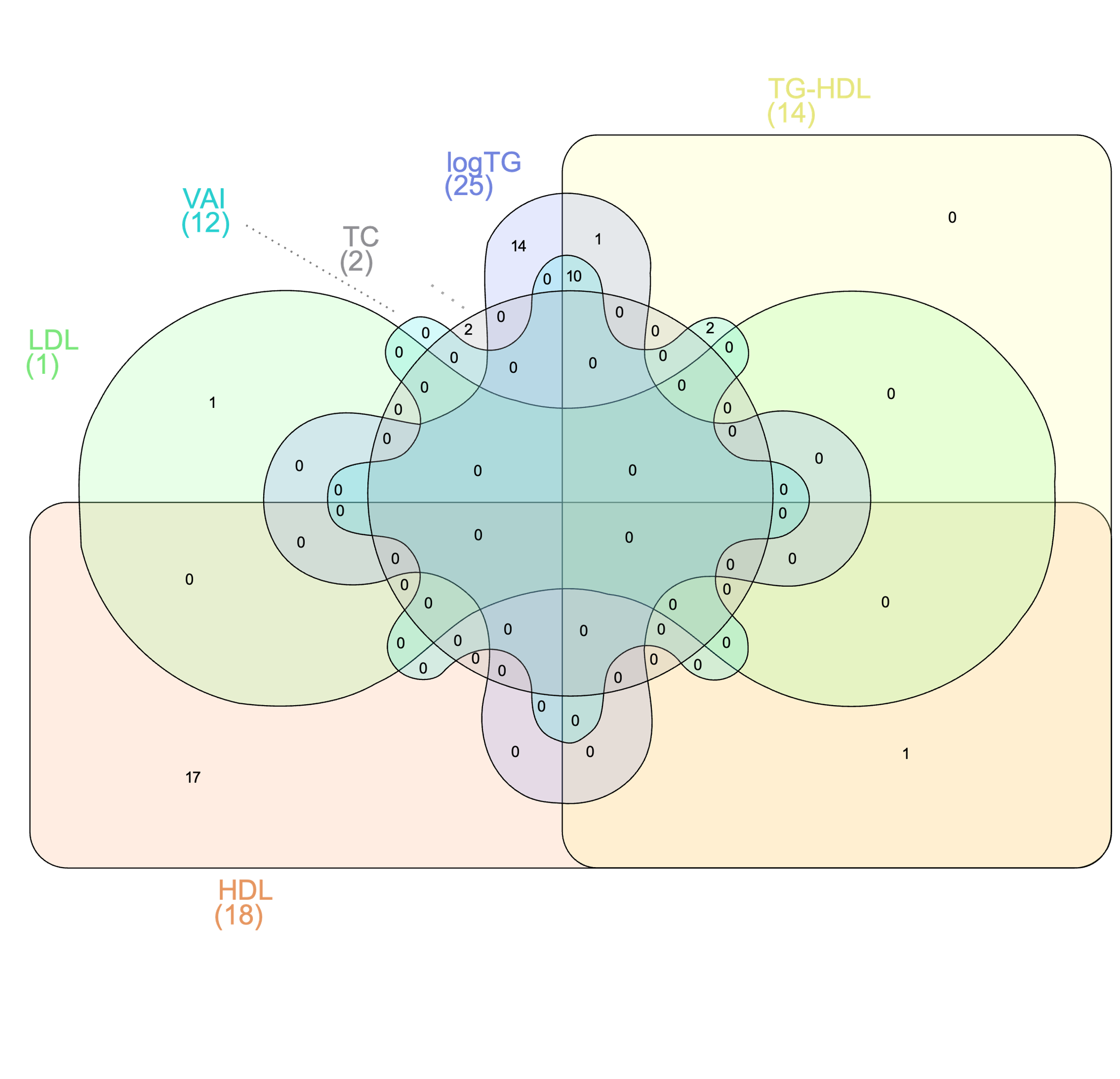
**

Figure S3: QQ plots for single variant associations

Figure S4: Manhattan plots for rare, low frequency and common variants for traits represented in Figure 2A

p<5*10^-8^ highlighted in red. The sub-plots in the third panel for each trait, representing common variants are also provided in main text, figure 2A

Figure S5: Manhattan plots for Name Recognition (memory) Naming Associations( executive), Geometric figures(Visuospatial) and Delayed Recall( Memory)

Top hit (independent sub-genome wide) high-confidence loci are annotated in red. The notable signals of particular interest, highlighted in black circles and annotated (even if they are not “high confidence” as per the definition in this study) are regions of particular interest which are known to have potential links to cognition (as discussed in section 3.2 of main text).

Figure S6: Gene expression heatmap for cognition associated genes (P<5*10^-6^)


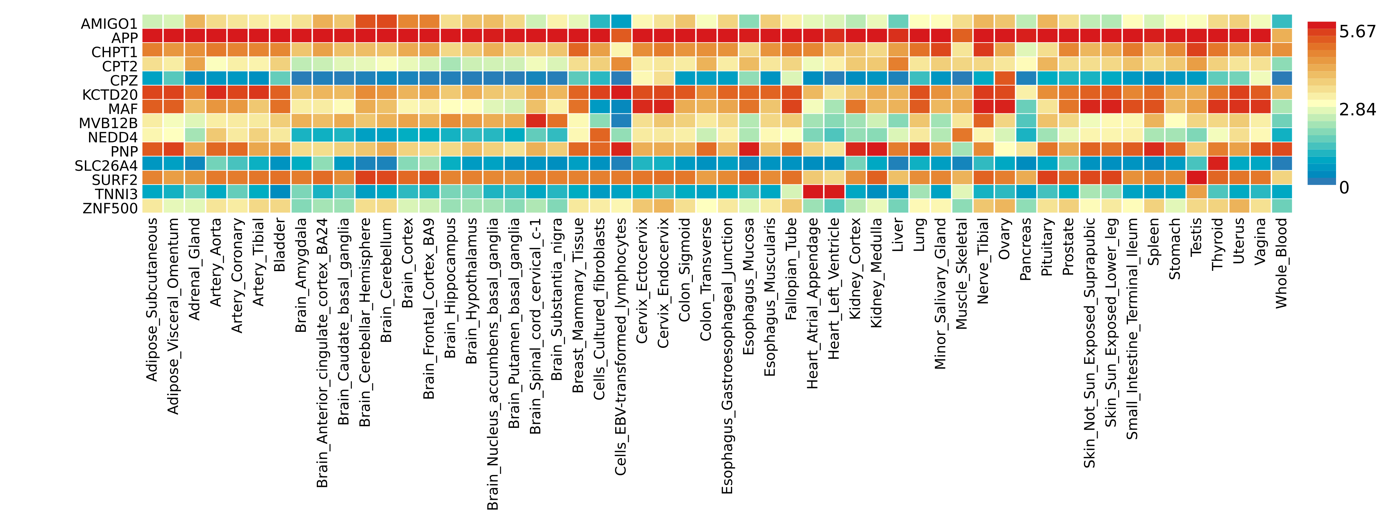


Figure S7: Gene expression heatmap for lipid (HDL, LDL, triglycerides, total cholesterol) associated genes


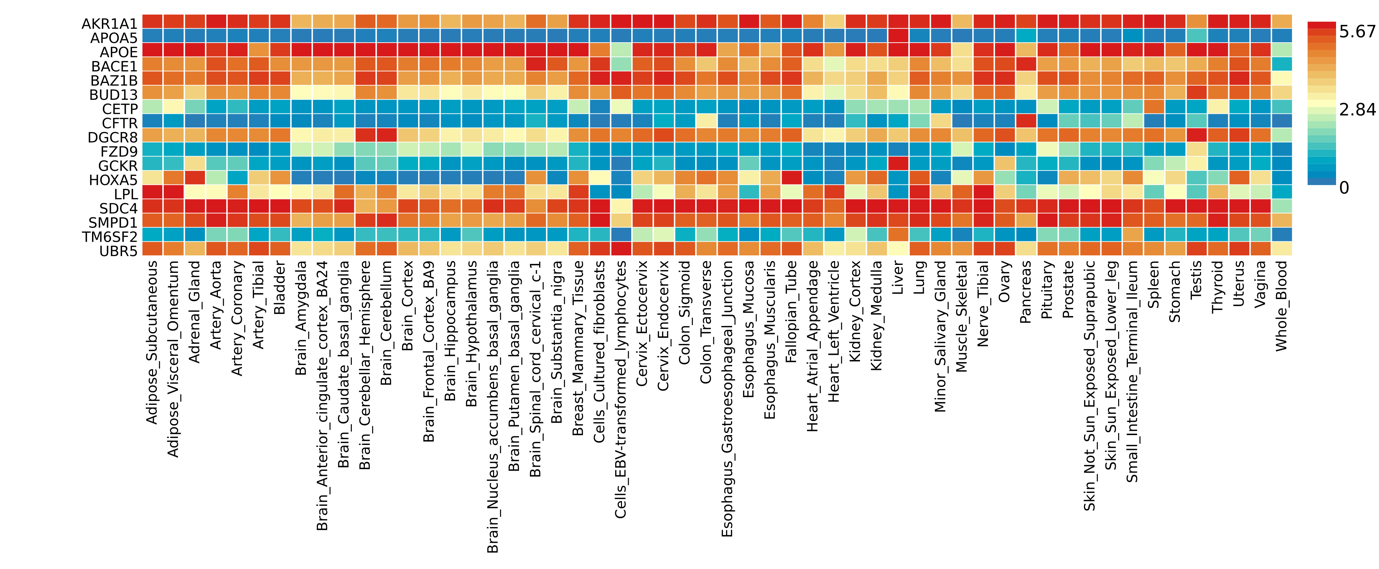


Figure S8: Gene expression heatmap for visceral adiposity- associated genes


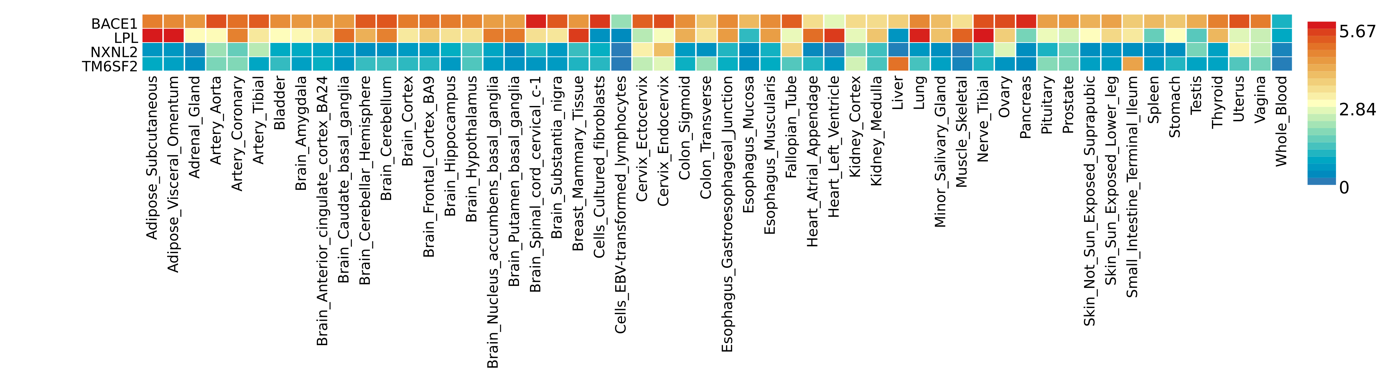


Figure S9: Gene expression heatmap for insulin resistance- associated genes


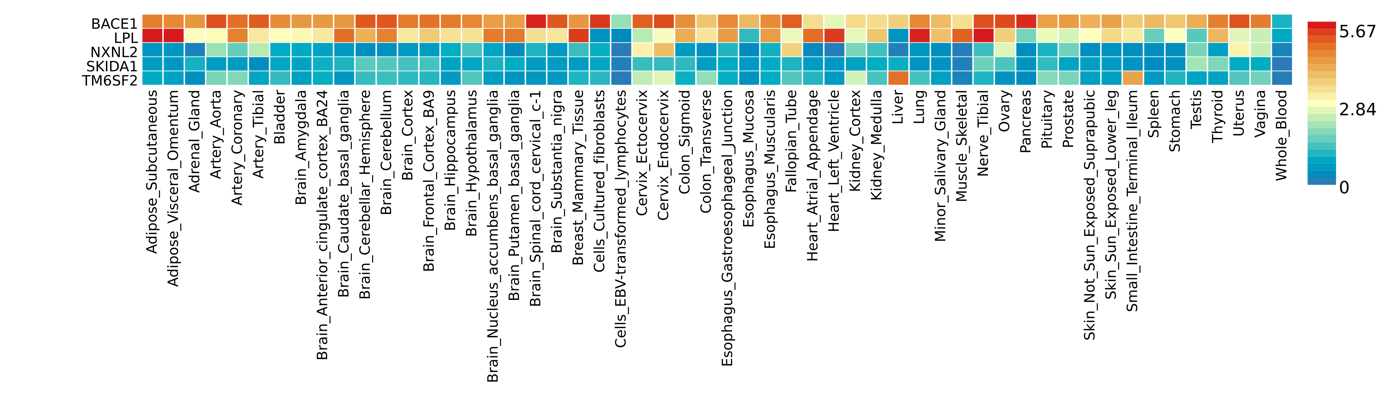


Figure S10: Mendelian randomization assessing adverse causal effect of HDL on Name-Face associations(memory)


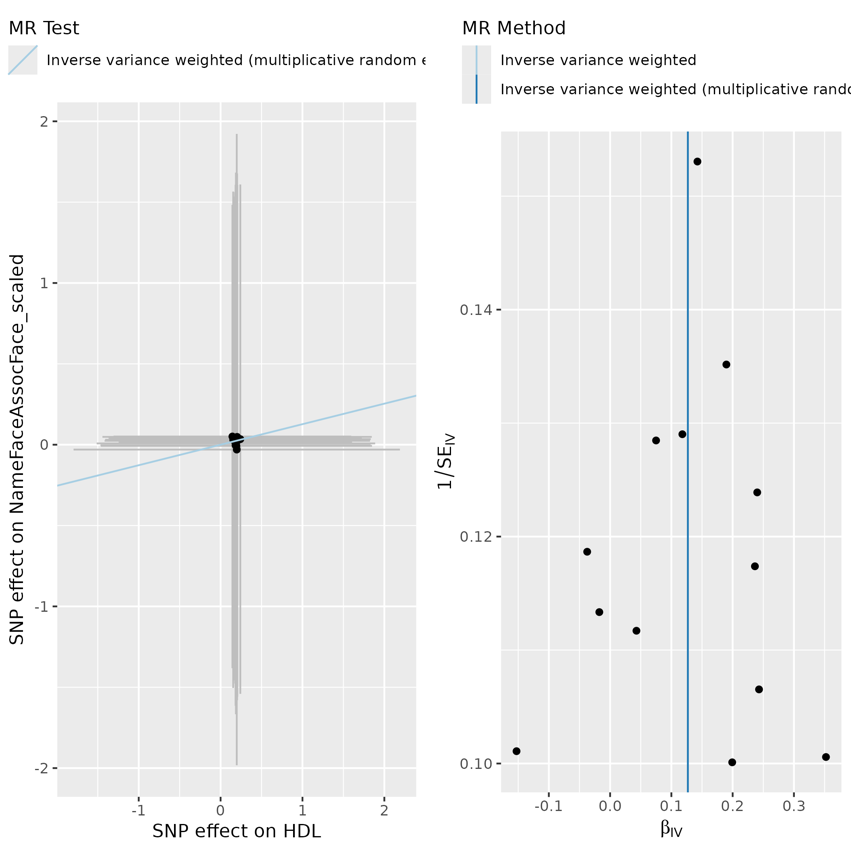


*Figure S11: Mendelian randomization assessing adverse causal effect of HDL on geometric figures (visuospatial)*


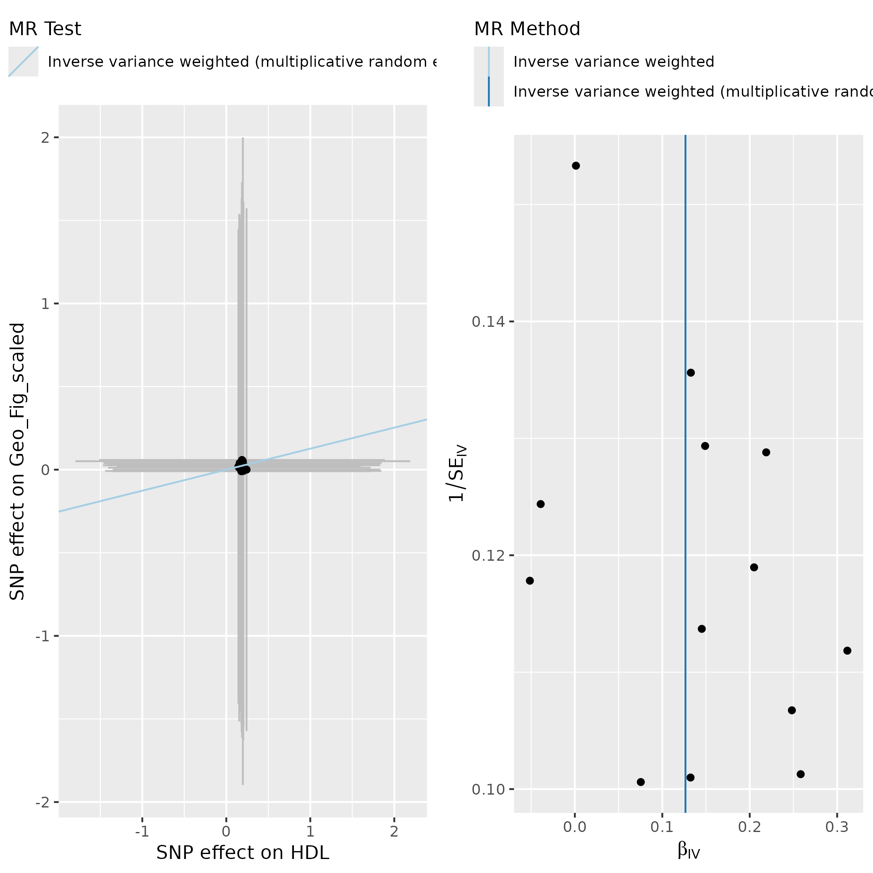


Figure S12: Mendelian randomization assessing adverse causal effect of triglycerides on Name-Face associations (memory)


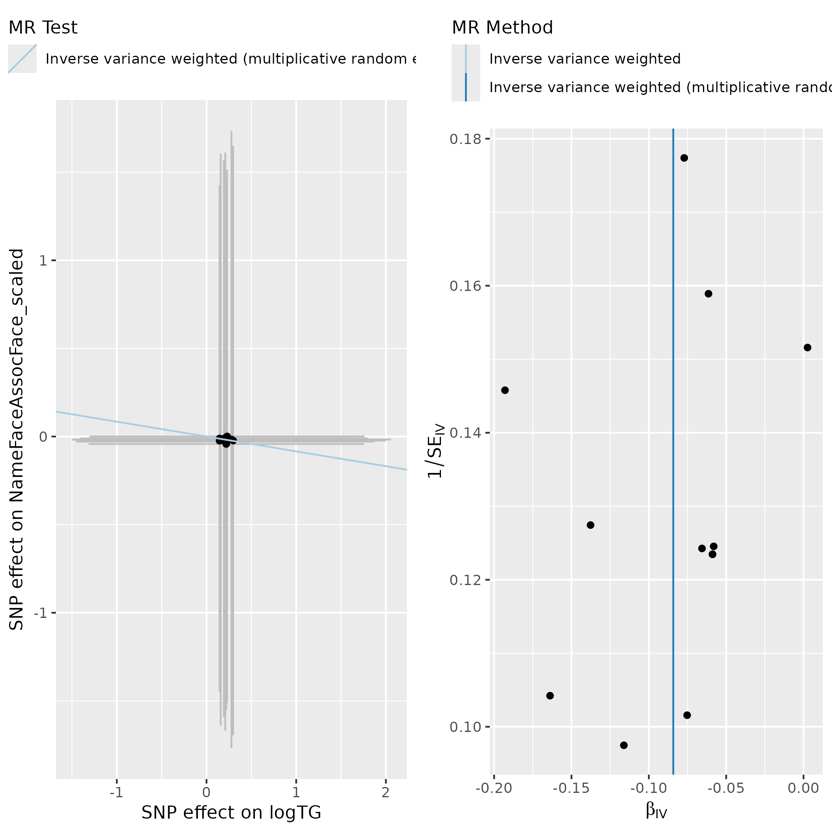


Figure S13: Mendelian randomization assessing adverse causal effect of TG-HDL ratio on Name-Face associations (memory)

*
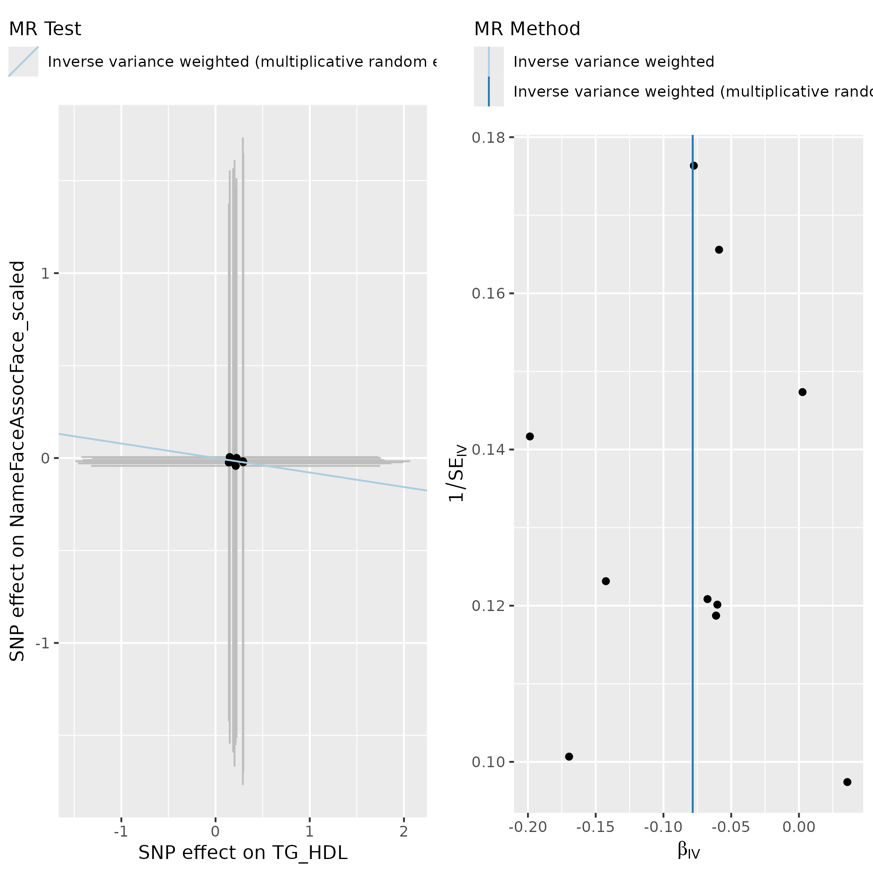
*

Figure S14: Mendelian randomization assessing adverse causal effect of VAI on Name-Face associations


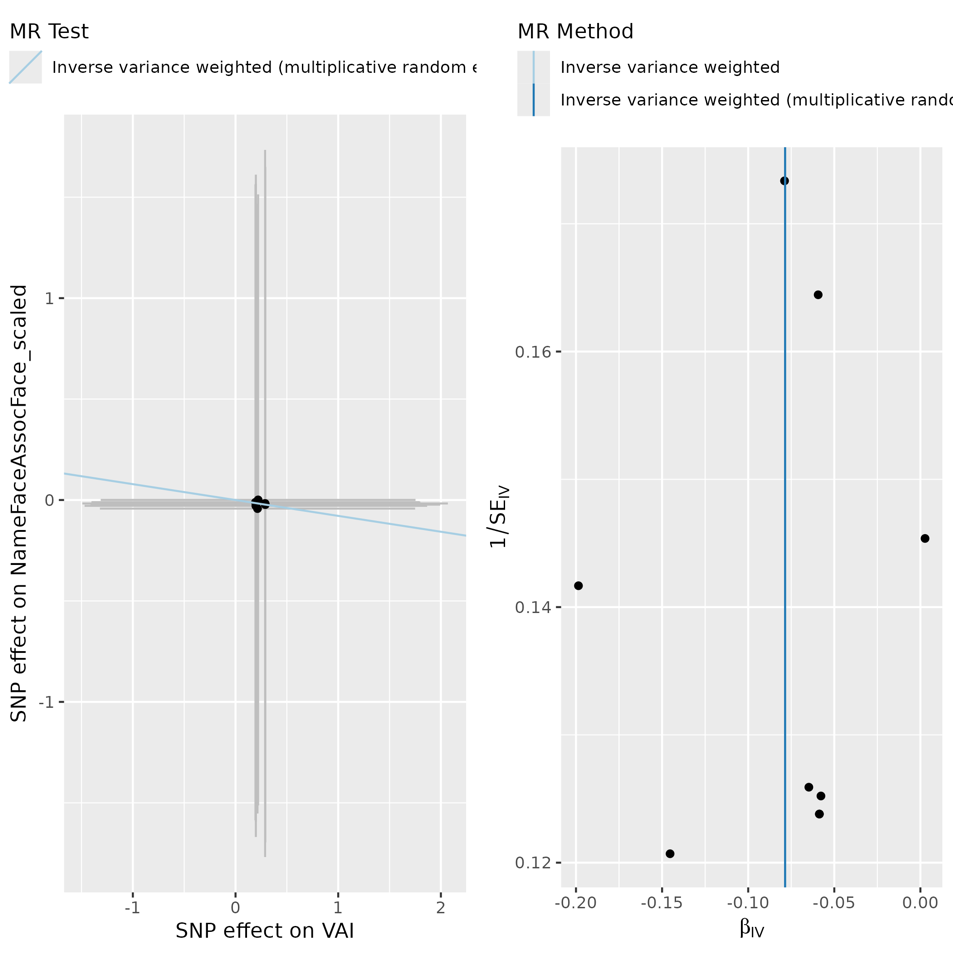


Figure S15: Mendelian randomization assessing adverse causal effect of triglycerides on mean reaction time (attention)


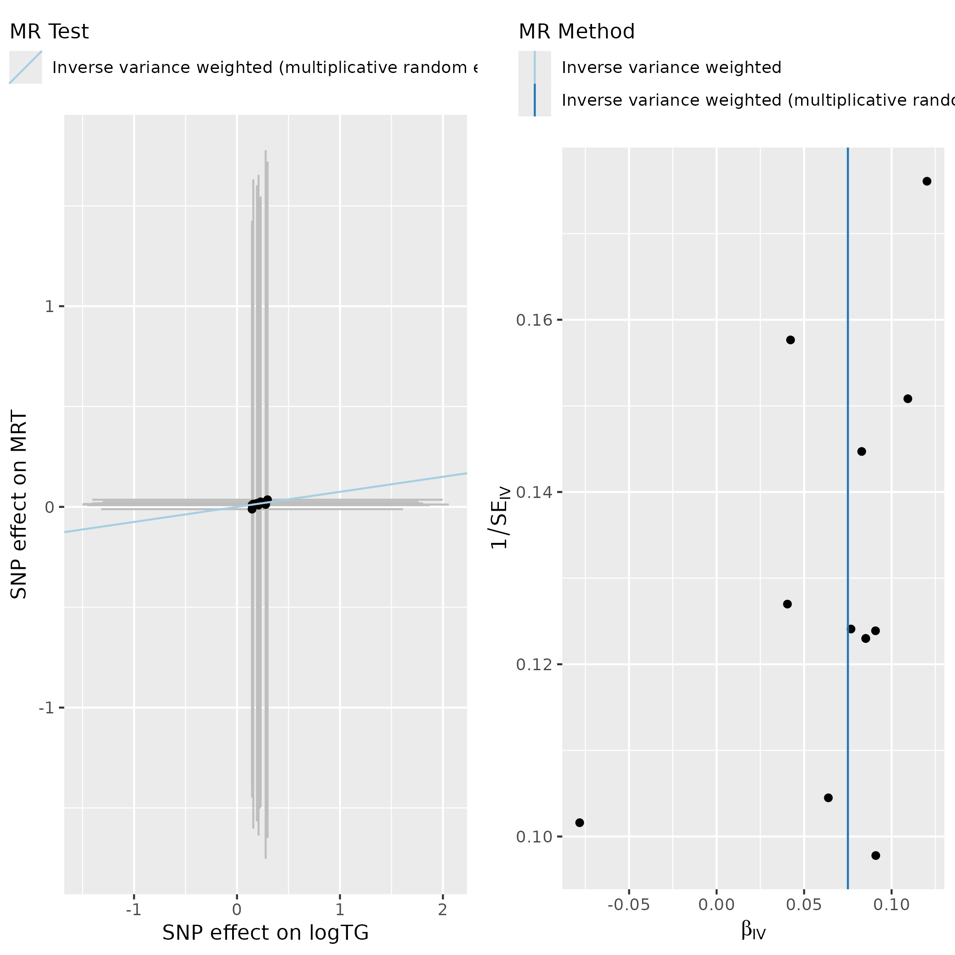


Figure S16: Mendelian randomization assessing adverse causal effect of VAI on reaction time (attention)


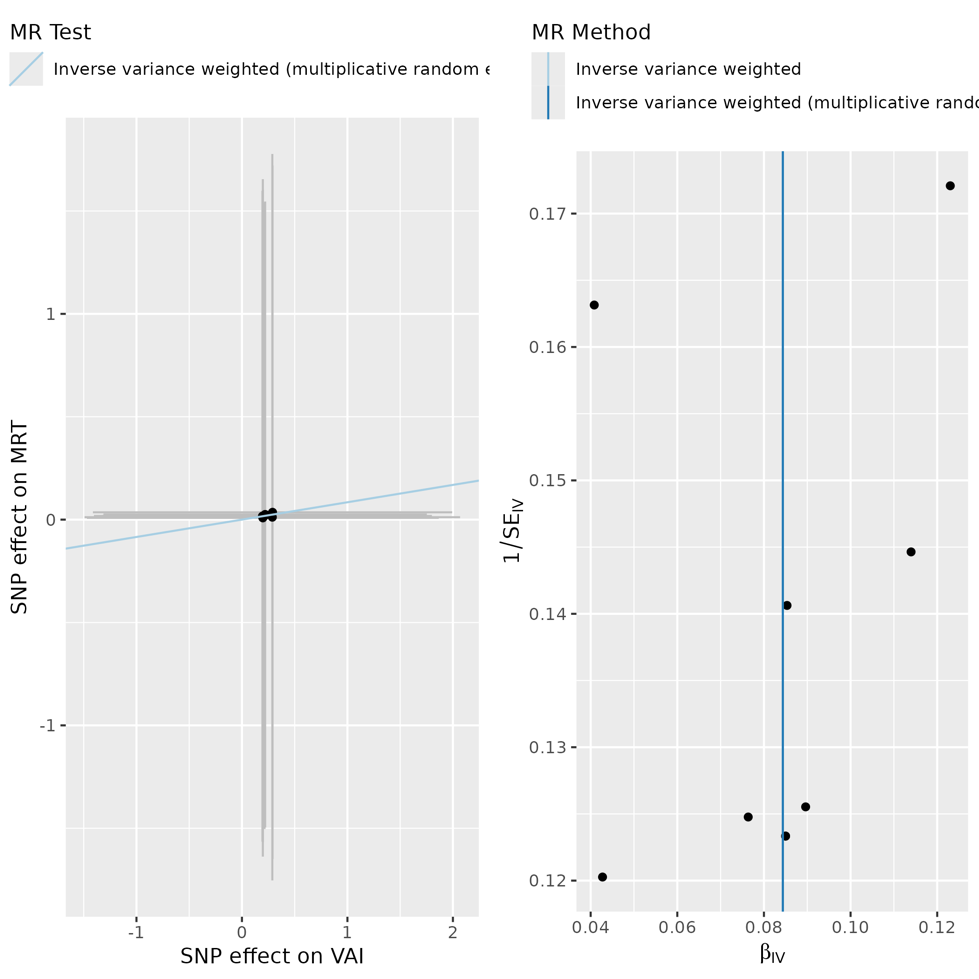


Table S1: Quality control steps for genotyped and imputed data

| **QC Steps on array data** | **Total samples** |  | **Total variants** |  |
| --- | --- | --- | --- | --- |
|  | 5111 |  | 892,508 |  |
| **Sample QC** | **Total samples retained** | **#Excluded samples** | **Total variants retained** | **#Excluded variants** |
| 1.Sample call rate (90%) | 5,111 | 0 | 892,508 | - |
| 2.Sex discrepancy(2462 M/2513 F ; 136 ambiguous) | 5,111 | 0 | 892,508 | - |
| 3.Heterozygosity (+-4sd) | 5,111 | - | 892,508 | - |
| **Variant QC** | **Total samples retained** | **#Excluded samples** | **Total variants retained** | **#Excluded variants** |
| 4. Biallelic only | 5,111 | - | 892,400 | 108 |
| 5. Autosomes only | 5,111 | - | 856,605 | 35,795 |
| 5. Duplicate SNPS | 5,111 | - | 851,441 | 5,164 |
| 6. Genotype Missingness (98%) | 5,111 | - | 750,983 | 100,458 |
| 7. MAC (>=2) | 5,111 | - | 466,108 | 284,875 |
| 8. HWE (10^-7) | 5,111 | - | 462,765 | 3,343 |
| 9. Removing INDELS above length 30 and Structural Variants | 5,111 |  | 462,718 | 47 |
| 10. Removing Duplicate sites  (norm -d all) | 5,111 |  | 462,712 | 6 |
| 11. Removing monomorphic sites (filter -e 'AC==0 \|\| AC==AN') | 5,111 |  | 462,712 | 0 |
| **Pre-Imputation** | **5,111** |  | **462,712** |  |
| **Post-Imputation (raw)** | **5,111** |  | **28,471,101** |  |
| **Post-Imputation (filtered-R^2^, AF, MAF)** | **5,111** |  | **16,942,745** |  |

Table S2: Narrow sense heritability estimates

| **Phenotype** | **N** | **h2** | **SE** |
| --- | --- | --- | --- |
| Auditory attention | 3385 | 25.32% | 0.0604 |
| Comprehension | 4128 | 17.13% | 0.0478 |
| Delayed Recall | 4015 | 11.75% | 0.0478 |
| Dual attention | 1672 | 21.14% | 0.1071 |
| Fasting Blood Sugar | 3987 | 40.67% | 0.0465 |
| Geometric Figures | 4414 | 12.70% | 0.0471 |
| G factor | 2109 | 44.25% | 0.0971 |
| HbA1c | 3987 | 17.16% | 0.0448 |
| HDL | 3982 | 42.83% | 0.0472 |
| HMSE | 4122 | 15.39% | 0.0465 |
| Immediate Recall | 4122 | 25.07% | 0.0486 |
| LDL | 3943 | 16.11% | 0.0493 |
| logTG | 3982 | 36.79% | 0.0475 |
| MRT | 3600 | 11.08% | 0.0514 |
| Name Face Association Face | 4117 | 8.65% | 0.0447 |
| Name Recognition | 4104 | 17.64% | 0.0491 |
| Naming Association | 3889 | 11.04% | 0.0504 |
| Phonetic | 3943 | 13.04% | 0.0497 |
| Reading | 4129 | 12.72% | 0.0464 |
| Semantic | 4149 | 15.39% | 0.0462 |
| Span | 3415 | 13.49% | 0.0574 |
| Stroop test | 1955 | 25.59% | 0.0909 |
| TC | 3982 | 42.99% | 0.0460 |
| TG:HDL | 3982 | 34.87% | 0.0474 |
| TMTB-TMTA | 1617 | 30.72% | 0.0985 |
| VAI | 3886 | 40.22% | 0.0507 |
| Visceral Fat | 4403 | 28.52% | 0.0440 |
| Visual attention | 3284 | 20.97% | 0.0597 |
| Vocabulary | 1272 | 46.88% | 0.1249 |
| *Metabolic syndrome | 3792 | 31.95% | 0.0500 |

*Raw heritability-> without covariate adjustment

Table S3: High confidence sub-genome wide (P<5*10^-6^) single variant associations with cognitive phenotypes

| **HMSE; N=4122** | | | | | | | | | | |
| --- | --- | --- | --- | --- | --- | --- | --- | --- | --- | --- |
| ***ID*** | ***AF*** | ***Beta*** | ***SE*** | ***P*** | ***rsid*** | ***R^2^*** | **Consequence** | **Gene** | **CADD-v1.7** | **Novel** |
| 13:105636852:T:C | 0.198 | 0.142 | 1.738 | 1.44E-07 | rs2111799 | 0.996 | Intergenic | *DAOA-AS1-LINC00343* | 0.634 | Yes |
| 19:32858582:T:C | 0.743 | -0.118 | 1.634 | 3.76E-06 | rs2287879 | 0.929 | intron | *SLC7A9* | 0.016 | Yes |
| 12:55639577:C:CT | 0.543 | -0.101 | 1.403 | 3.95E-06 | rs34355759 | 0.918 | downstream | *OR10P1* | - | Yes |
| **G-factor ; N=2109** | | | | | | | | | | |
| ***ID*** | ***AF*** | ***Beta*** | ***SE*** | ***P*** | ***rsid*** | ***R^2^*** | **Consequence** | **Gene** | **CADD-v1.7** | **Novel** |
| 18:65364664:T:C | 0.220 | 0.179 | 1.689 | 1.17E-06 | rs616329 | 0.718 | intergenic | *LINC01924-CDH7* | 4.178 | Yes |
| 1:220406830:A:G | 0.850 | -0.199 | 1.948 | 2.73E-06 | rs972980 | 0.957 | intergenic | *RAB3GAP2-MARK1* | 0.149 | Yes |
| **COGNITO Attention: Auditory attention; N=3385** | | | | | | | | | | |
| ***ID*** | ***AF*** | ***Beta*** | ***SE*** | ***P*** | ***rsid*** | ***R^2^*** | **Consequence** | **Gene** | **CADD-v1.7** | **Novel** |
| 14:54782627:C:T | 0.358 | -0.125 | 1.458 | 5.55E-07 | rs709939 | 0.977 | intron | *SAMD4A* | 0.044 | Yes |
| 18:59151831:C:T | 0.169 | -0.155 | 1.903 | 2.21E-06 | rs7241847 | 0.944 | Intron | *SEC11C* | 1.315 | Yes |
| 15:96179698:CT:C | 0.527 | -0.113 | 1.402 | 3.03E-06 | rs67098761 | 0.926 | Intron | *NR2F2-AS1* | - | Yes |
| 7:68232630:A:AAAAAAAAAAG | 0.159 | 0.150 | 1.897 | 4.25E-06 | rs1554329499 | 0.934 | intergenic | *LOC102723427-CT66* | 4.085 | Yes |
| 7:68257922:C:CA | 0.156 | 0.151 | 1.917 | 4.89E-06 | rs34015912 | 0.905 | intergenic | *LOC102723427-CT66* | 0.945 | Yes |
| **COGNITO Attention: Visual attention; N=3284** | | | | | | | | | | |
| ***ID*** | ***AF*** | ***Beta*** | ***SE*** | ***P*** | ***rsid*** | ***R^2^*** | **Consequence** | **Gene** | **CADD-v1.7** | **Novel** |
| 4:80965183:A:G | 0.789 | -0.152 | 1.712 | 3.41E-07 | rs13144676 | 0.982 | downstream | *CFAP299* | 3.778 | Yes |
| 2:160178689:T:TC | 0.394 | -0.119 | 1.424 | 1.70E-06 | rs373599476 | 0.922 | intron | *ITGB6* | 1.857 | Yes |
| **COGNITO Attention: Dual attention; N=1672** | | | | | | | | | | |
| ***ID*** | ***AF*** | ***Beta*** | ***SE*** | ***P*** | ***rsid*** | ***R^2^*** | **Consequence** | **Gene** | **CADD-v1.7** | **Novel** |
| 10:8209344:G:A | 0.431 | -0.180 | 1.441 | 3.22E-07 | rs10905315 | 1 | intergenic | *GATA3-LINC00708* | 5.194 | Yes |
| 10:18398684:CACACACAT:C | 0.195 | -0.202 | 1.735 | 2.03E-06 | rs138019458 | 0.927 | Upstream | *CACNB2* | - | Yes |
| **COGNITO Attention: Mean Reaction Time; N=3600** | | | | | | | | | | |
| ***ID*** | ***AF*** | ***Beta*** | ***SE*** | ***P*** | ***rsid*** | ***R^2^*** | **Consequence** | **Gene** | **CADD-v1.7** | **Novel** |
| 10:12320874:T:C | 0.823 | 0.155 | 1.846 | 4.62E-07 | rs4363492 | 0.804 | intergenic | *CDC123-CAMK1D* | 0.965 | Yes |
| 1:64724068:C:T | 0.288 | -0.127 | 1.552 | 8.63E-07 | rs3920616 | 0.968 | intergenic | *CACHD1-RAVER2* | 0.063 | Yes |
| 12:92526181:A:G | 0.336 | -0.120 | 1.481 | 1.23E-06 | rs10492342 | 0.996 | intergenic | *LINC02397-C12orf74* | 4.085 | Yes |
| 20:49599899:G:A | 0.725 | 0.126 | 1.576 | 1.74E-06 | rs522411 | 0.909 | intergenic | *PTGIS-B4GALT5* | 3.842 | Yes |
| 2:75791505:T:TTTTG | 0.526 | -0.111 | 1.404 | 2.01E-06 | rs10659269 | 0.977 | intergenic | *-* | - | Yes |
| 15:44763054:C:T | 0.422 | 0.114 | 1.444 | 2.02E-06 | rs2468071 | 0.935 | intron | *TRIM69* | 5.522 | Yes |
| 15:44777457:T:G | 0.274 | 0.122 | 1.589 | 4.43E-06 | rs12372957 | 0.946 | intergenic | *TRIM69-SORD2P* | 4.898 | Yes |
| **COGNITO Memory: Delayed Recall; N=4015** | | | | | | | | | | |
| ***ID*** | ***AF*** | ***Beta*** | ***SE*** | ***P*** | ***rsid*** | ***R^2^*** | **Consequence** | **Gene** | **CADD-v1.7** | **Novel** |
| 2:15621929:T:C | 0.817 | -0.140 | 1.821 | 1.16E-06 | rs6755613 | 0.996 | intron | *DDX1* | 1.287 | Yes |
| 10:52934190:A:G | 0.195 | 0.131 | 1.753 | 2.17E-06 | rs12356267 | 0.875 | intergenic | *MBL2-PCDH15* | 0.317 | Yes |
| 17:54491945:T:C | 0.265 | -0.116 | 1.578 | 2.94E-06 | rs1896880 | 0.973 | intergenic | *KIF2B-TOM1L1* | 2.019 | Yes |
| 1:109503258:A:C | 0.230 | 0.121 | 1.651 | 3.15E-06 | rs4370783 | 0.962 | downstream | *CYB561D1* | 11.42 | Yes |
| 16:85646766:G:A | 0.195 | 0.128 | 1.750 | 3.64E-06 | rs12600148 | 0.91 | intron | *GSE1* | 4.331 | Yes |
| 17:54495540:T:C | 0.180 | -0.131 | 1.808 | 4.46E-06 | rs1991548 | 1 | intergenic | *KIF2B-TOM1L1* | 1.035 | Yes |
| **COGNITO Memory: Immediate Recall; N=4122** | | | | | | | | | | |
| ***ID*** | ***AF*** | ***Beta*** | ***SE*** | ***P*** | ***rsid*** | ***R^2^*** | **Consequence** | **Gene** | **CADD-v1.7** | **Novel** |
| 11:69255561:G:A | 0.146 | 0.150 | 1.983 | 1.21E-06 | rs11228593 | 0.949 | intergenic | *LOC338694-MYEOV* | 5.428 | Yes |
| 3:31922416:C:T | 0.363 | 0.107 | 1.449 | 1.91E-06 | rs11914604 | 0.978 | intron | *OSBPL10* | 0.749 | Yes |
| 15:74336550:A:G | 0.419 | 0.104 | 1.412 | 2.06E-06 | rs2959003 | 0.973 | downstream | *CCDC33* | 6.95 | Yes |
| 9:100475715:CTGTG:C | 0.247 | 0.115 | 1.614 | 4.90E-06 | rs3055671 | 0.927 | intronic | *MSANTD3-TMEFF1* | - | Yes |
| **COGNITO Memory: Name Face Assoc Face; N=4117** | | | | | | | | | | |
| ***ID*** | ***AF*** | ***Beta*** | ***SE*** | ***P*** | ***rsid*** | ***R^2^*** | **Consequence** | **Gene** | **CADD-v1.7** | **Novel** |
| 12:16600418:C:T | 0.823 | 0.135 | 1.839 | 2.37E-06 | rs10846382 | 1 | intron | *LMO3* | 5.428 | Yes |
| **COGNITO Memory: Name Recognition; N=4104** | | | | | | | | | | |
| ***ID*** | ***AF*** | ***Beta*** | ***SE*** | ***P*** | ***rsid*** | ***R^2^*** | **Consequence** | **Gene** | **CADD-v1.7** | **Novel** |
| 14:85766662:C:T | 0.338 | 0.130 | 1.490 | 2.57E-08 | rs12588896 | 0.967 | intergenic | *FLRT2-LINC02328* | 1.002 | Yes |
| 7:79494149:T:A | 0.641 | -0.122 | 1.461 | 8.38E-08 | rs12534627 | 0.703 | intergenic | *MAGI2-AS3-GNAI1* | 0.141 | Yes |
| 8:10582851:T:C | 0.177 | 0.150 | 1.854 | 2.06E-07 | rs35626194 | 1 | intergenic | *PRSS55-RP1L1* | 1.022 | Yes |
| 11:127507002:T:C | 0.153 | 0.146 | 1.941 | 1.36E-06 | rs10790897 | 0.967 | intergenic | *LINC02712-LINC02098* | 0.571 | Yes |
| 14:85771931:A:G | 0.462 | 0.106 | 1.413 | 1.60E-06 | rs11159731 | 1 | intergenic | *FLRT2-LINC02328* | 1.472 | Yes |
| 14:85761193:C:T | 0.447 | 0.105 | 1.422 | 2.47E-06 | rs10140466 | 0.997 | intergenic | *FLRT2-LINC02328* | 1.554 | Yes |
| 4:76281999:A:G | 0.419 | 0.105 | 1.424 | 2.51E-06 | rs4859659 | 0.961 | intron | *FAM47E-STBD1* | 3.443 | Yes |
| **COGNITO Visuospatial: Span; N=3415** | | | | | | | | | | |
| ***ID*** | ***AF*** | ***Beta*** | ***SE*** | ***P*** | ***rsid*** | ***R^2^*** | **Consequence** | **Gene** | **CADD-v1.7** | **Novel** |
| 13:98780095:ATG:A | 0.167 | -0.167 | 1.869 | 1.64E-07 | rs68009296 | 0.946 | intergenic | *SLC15A1-DOCK9* | 0.919 | Yes |
| 20:36137680:G:A | 0.289 | 0.122 | 1.545 | 3.86E-06 | rs68009296 | 0.988 | intron | *EPB41L1* | 0.885 | Yes |
| 20:36171133:C:CT | 0.219 | 0.133 | 1.689 | 4.41E-06 | rs2378439 | 0.971 | intron | *EPB41L1* | - | Yes |
| 20:36072858:CAT:C | 0.174 | 0.144 | 1.833 | 4.43E-06 | rs11484332 | 0.966 | intergenic | *NORAD-EPB41L1* | - | Yes |
| 20:36130787:T:TTC | 0.260 | 0.124 | 1.587 | 4.71E-06 | rs10604564 | 0.882 | intron | *EPB41L1* | - | Yes |
| **COGNITO Visuospatial: Stroop Test; N=1955** | | | | | | | | | | |
| ***ID*** | ***AF*** | ***Beta*** | ***SE*** | ***P*** | ***rsid*** | ***R^2^*** | **Consequence** | **Gene** | **CADD-v1.7** | **Novel** |
| 7:213090:G:A | 0.487 | 0.147 | 1.396 | 3.09E-06 | rs35745428 | 0.912 | intron | FAM20C | 2.736 | Yes |
| **COGNITO Visuospatial: Geometric figures; N=4414** | | | | | | | | | | |
| ***ID*** | ***AF*** | ***Beta*** | ***SE*** | ***P*** | ***rsid*** | ***R^2^*** | **Consequence** | **Gene** | **CADD-v1.7** | **Novel** |
| 6:55338468:T:C | 0.265 | 0.121 | 1.581 | 8.53E-07 | rs9349776 | 0.988 | intron | *GFRAL* | 2.857 | Yes |
| 9:25741430:G:A | 0.606 | 0.104 | 1.435 | 3.45E-06 | rs1231341 | 0.989 | intergenic | *TUSC1-LINC01241* | 1.039 | Yes |
| 10:53171210:G:A | 0.302 | -0.109 | 1.524 | 3.98E-06 | rs12257790 | 0.994 | intergenic | *MBL2-PCDH15* | 0.236 | Yes |
| **Executive functioning: TMT B-A; N=1617** | | | | | | | | | | |
| ***ID*** | ***AF*** | ***Beta*** | ***SE*** | ***P*** | ***rsid*** | ***R^2^*** | **Consequence** | **Gene** | **CADD-v1.7** | **Novel** |
| 5:170129930:G:T | 0.335 | -0.180 | 1.494 | 1.27E-06 | rs11134615 | 0.999 | intergenic | *FOXI1-LINC01187* | 0.381 | Yes |
| 3:133095359:G:T | 0.278 | -0.187 | 1.566 | 1.68E-06 | rs10935046 | 0.986 | intron | *TMEM108* | 10.42 | Yes |
| 16:75186111:C:A | 0.809 | 0.208 | 1.782 | 2.77E-06 | rs7205074 | 1 | intergenic | *ZFP1-CTRB2* | 0.259 | Yes |
| 14:21353662:A:C | 0.602 | -0.162 | 1.406 | 3.64E-06 | rs3736824 | 0.947 | downstream | *RPGRIP1* | 8.541 | Yes |
| 19:4457794:TTAA:T | 0.519 | 0.161 | 1.415 | 4.84E-06 | rs58895127 | 0.733 | upstream | *UBXN6* | - | Yes |
| **Executive functioning: Naming association (Semantic association) ; N=3889** | | | | | | | | | | |
| ***ID*** | ***AF*** | ***Beta*** | ***SE*** | ***P*** | ***rsid*** | ***R^2^*** | **Consequence** | **Gene** | **CADD-v1.7** | **Novel** |
| 4:156203576:A:G | 0.309 | -0.129 | 1.528 | 1.36E-07 | rs13103675 | 0.986 | intergenic | *CTSO-PDGFC* | 0.127 | Yes |
| 4:95377791:T:C | 0.304 | 0.115 | 1.486 | 1.34E-06 | rs6822806 | 0.938 | intron | *UNC5C* | 18.33 | Yes |
| 11:99104616:G:GTA | 0.368 | 0.111 | 1.459 | 1.97E-06 | rs34078461 | 0.974 | intron | *CNTN5* | 0.194 | Yes |
| 4:156225559:G:A | 0.411 | -0.108 | 1.429 | 2.57E-06 | rs10027875 | 0.985 | intergenic | *CTSO-PDGFC* | 0.886 | Yes |
| 10:120263197:C:T | 0.158 | 0.145 | 1.934 | 2.78E-06 | rs111622984 | 0.922 | intergenic | *MIR4682-RPL21* | 1.589 | Yes |
| 12:60132856:T:C | 0.448 | -0.107 | 1.418 | 2.80E-06 | rs11173335 | 0.991 | intergenic | *SLC16A7-TAFA2* | 0.466 | Yes |
| 11:99121980:C:CAT | 0.177 | -0.138 | 1.847 | 3.00E-06 | rs10673411 | 0.918 | intron | *CNTN5* | 0.136 | Yes |
| **COGNITO Language: Comprehension; N=4128** | | | | | | | | | | |
| ***ID*** | ***AF*** | ***Beta*** | ***SE*** | ***P*** | ***rsid*** | ***R^2^*** | **Consequence** | **Gene** | **CADD-v1.7** | **Novel** |
| 3:119325647:A:G | 0.616 | 0.116 | 1.440 | 2.34E-07 | rs935619 | 0.967 | upstream_ | *ARHGAP31-AS1* | 0.919 | Yes |
| 11:31515974:A:G | 0.378 | 0.108 | 1.429 | 1.32E-06 | rs523763 | 0.98 | intron | *ELP4* | 0.831 | Yes |
| 8:113848736:T:C | 0.407 | 0.106 | 1.423 | 1.63E-06 | rs28795772 | 0.996 | intergenic | *CSMD3-TRPS1* | 1.957 | Yes |
| 15:23841077:AAC:A | 0.685 | -0.111 | 1.522 | 2.57E-06 | rs34440064 | 0.817 | intergenic | *NDN-PWRN4* | - | Yes |
| 3:119310723:G:A | 0.496 | 0.104 | 1.416 | 2.61E-06 | rs62265237 | 0.992 | downstream | *ARHGAP31-AS1* | 0.595 | Yes |
| 3:119312294:C:G | 0.722 | 0.115 | 1.581 | 3.18E-06 | rs2044620 | 0.976 | downstream | *ARHGAP31-AS1* | 7.349 | Yes |
| 8:113777753:G:A | 0.477 | -0.099 | 1.382 | 4.59E-06 | rs9297509 | 0.952 | intergenic | *CSMD3-TRPS1* | 7.518 | Yes |
| **COGNITO Language: Reading; N=4129** | | | | | | | | | | |
| ***ID*** | ***AF*** | ***Beta*** | ***SE*** | ***P*** | ***rsid*** | ***R^2^*** | **Consequence** | **Gene** | **CADD-v1.7** | **Novel** |
| 13:68058771:G:C | 0.162 | 0.147 | 1.885 | 5.23E-07 | rs73210729 | 0.907 | intergenic | *LINC00364-LINC00550* | 0.198 | Yes |
| 15:55302749:A:G | 0.446 | 0.100 | 1.384 | 3.46E-06 | rs199804371 | 0.912 | intergenic | *RAB27A-PIGBOS1* | 6.2 | Yes |
| 3:20333437:A:G | 0.262 | 0.115 | 1.606 | 4.31E-06 | rs6784144 | 0.814 | intergenic | *SGO1-LOC101927829* | 0.159 | Yes |
| **COGNITO Language: Phonetic; N=3943** | | | | | | | | | | |
| ***ID*** | ***AF*** | ***Beta*** | ***SE*** | ***P*** | ***rsid*** | ***R^2^*** | **Consequence** | **Gene** | **CADD-v1.7** | **Novel** |
| 11:47358789:G:T | 0.697 | 0.113 | 1.524 | 3.35E-06 | rs3740688 | 0.95 | intron | *SPI1* | 0.034 | Yes# |
| 2:209327312:C:T | 0.602 | -0.104 | 1.419 | 4.57E-06 | rs62215366 | 0.985 | intergenic | *-* | 1.154. | Yes |
| 11:46926127:C:CT | 0.326 | -0.109 | 1.491 | 4.88E-06 | rs11439494 | 0.913 | intergenic | *LRP4-C11orf49* | - | Yes |
| **COGNITO Language: Semantic; N=4149** | | | | | | | | | | |
| ***ID*** | ***AF*** | ***Beta*** | ***SE*** | ***P*** | ***rsid*** | ***R^2^*** | **Consequence** | **Gene** | **CADD-v1.7** | **Novel** |
| 13:87708423:TG:T | 0.442 | 0.104 | 1.422 | 2.49E-06 | rs5805460 | 0.969 | intergenic | *SLITRK5-LINC00397* | - | Yes |
| 4:110208933:C:T | 0.163 | 0.138 | 1.895 | 2.60E-06 | rs117276628 | 0.817 | intergenic | *ELOVL6-ENPEP* | 2.242 | Yes |
| **COGNITO Language: Vocabulary; N=1272** | | | | | | | | | | |
| ***ID*** | ***AF*** | ***Beta*** | ***SE*** | ***P*** | ***rsid*** | ***R^2^*** | **Consequence** | **Gene** | **CADD-v1.7** | **Novel** |
| 19:55901846:C:T | 0.744 | 0.240 | 1.623 | 1.38E-07 | rs302834 | 0.977 | intron | *NLRP13* | 4.725 | Yes |
| 2:77459612:A:AT | 0.288 | -0.211 | 1.583 | 2.11E-06 | rs34022356 | 0.825 | intron | *LRRTM4* | 3.055 | Yes |
| 9:104017159:C:T | 0.483 | 0.186 | 1.400 | 2.21E-06 | rs663859 | 0.994 | intergenic | *LOC101928523-SMC2-AS1* | 0.028 | Yes |
| 7:42192823:T:C | 0.388 | -0.193 | 1.465 | 2.51E-06 | rs10261063 | 0.955 | intron | *GLI3* | 0.032 | Yes |
| 11:131321601:AG:A | 0.284 | -0.206 | 1.578 | 3.37E-06 | rs11311461 | 1 | intergenic | *SNX19-NTM* | - | Yes |

#: Novel for cognition, but implicated previously in Alzheimer’s disease (familial, late onset)

Genome-wide significant (P<5*10^-8^) are also stated in Table 1

Table S4: High confidence sub-genome wide (P<5*10^-6^) single variant associations for cardiometabolic risks

| **Glycemic: FBS; N=3987** | | | | | | | | | | |
| --- | --- | --- | --- | --- | --- | --- | --- | --- | --- | --- |
| ***ID*** | ***AF*** | ***Beta*** | ***SE*** | ***P*** | ***rsid*** | ***R^2^*** | **Consequence** | **Gene** | **CADD-v1.7** | **Novel** |
| 13:91611870:A:G | 0.272 | -0.117 | 1.555 | 1.84E-06 | rs72632626 | 0.993 | intron | *GPC5* | 4.151 | Yes |
| 12:62686895:TG:T | 0.253 | 0.117 | 1.614 | 4.42E-06 | rs11310268 | 0.962 | intron | *PPM1H* | - | Yes |
| **Glycemic: HbA1c; N=3987** | | | | | | | | | | |
| ***ID*** | ***AF*** | ***Beta*** | ***SE*** | ***P*** | ***rsid*** | ***R^2^*** | **Consequence** | **Gene** | **CADD-v1.7** | **Novel** |
| 10:112996282:A:T | 0.307 | 0.119 | 1.542 | 1.21E-06 | rs4506565 | 1 | intron | *TCF7L2* | 0.985 | Yes |
| 10:113004902:G:A | 0.386 | 0.109 | 1.440 | 1.58E-06 | rs10787471 | 0.98 | intron | *TCF7L2* | 4.272 | Yes |
| 7:128513186:CTT:C | 0.504 | 0.103 | 1.429 | 4.91E-06 | rs71160662 | 0.971 | intergenic | *METTL2B-LINC01000* | - | Yes |
| **Lipid: HDL; N=3982** | | | | | | | | | | |
| ***ID*** | ***AF*** | ***Beta*** | ***SE*** | ***P*** | ***rsid*** | ***R^2^*** | **Consequence** | **Gene** | **CADD-v1.7** | **Novel** |
| 16:56953103:C:T | 0.272 | 0.241 | 1.593 | 1.29E-21 | rs12446515 | 0.95 | intergenic | *HERPUD1-CETP* | 2.108 | No |
| 16:56971389:C:T | 0.469 | 0.189 | 1.421 | 4.74E-17 | rs1532625 | 0.933 | splice&intron | *CETP* | 0.757 | No |
| 16:56961324:C:A | 0.600 | 0.180 | 1.425 | 1.39E-15 | rs1800775 | 1 | upstream | *CETP* | 0.409 | No |
| 16:56951643:A:G | 0.732 | 0.204 | 1.621 | 1.88E-15 | rs12448528 | 0.903 | intergenic | *HERPUD1-CETP* | 1.462 | No |
| 16:56967362:AC:A | 0.243 | 0.202 | 1.645 | 9.76E-15 | rs200751500 | 0.908 | intron | *CETP* | - | No |
| 16:56973534:T:G | 0.240 | -0.193 | 1.656 | 1.84E-13 | rs11076176 | 0.904 | intron | *CETP* | 2.296 | No |
| 16:56951602:G:A | 0.297 | 0.178 | 1.547 | 3.40E-13 | rs72786786 | 0.852 | intergenic | *HERPUD1-CETP* | 3.971 | No |
| 16:56957712:G:A | 0.248 | -0.183 | 1.650 | 2.89E-12 | rs28888131 | 1 | upstream | *CETP* | 1.122 | Yes |
| 16:56968820:T:G | 0.779 | 0.187 | 1.701 | 3.91E-12 | rs9939224 | 1 | intron | *CETP* | 1.041 | No |
| 16:56968751:C:G | 0.681 | 0.159 | 1.520 | 3.68E-11 | rs9926440 | 0.963 | intron | *CETP* | 1.643 | Yes |
| 16:56951227:A:G | 0.600 | 0.144 | 1.451 | 3.70E-10 | rs9989419 | 1 | intergenic | *HERPUD1-CETP* | 0.826 | No |
| 16:56967026:G:A | 0.149 | -0.197 | 1.993 | 4.16E-10 | rs118146573 | 1 | intron | *CETP* | 2.317 | No |
| 15:58431280:T:C | 0.284 | 0.154 | 1.563 | 5.63E-10 | rs1077834 | 1 | upstream | *LIPC* | 0.225 | No |
| 16:56964660:G:C | 0.332 | -0.142 | 1.511 | 2.94E-09 | rs9929488 | 0.947 | intron | *CETP* | 0.111 | Yes |
| 8:19966981:T:C | 0.282 | 0.148 | 1.581 | 3.52E-09 | rs13702 | 1 | 3_prime_UTR | *LPL* | 0.557 | No |
| 16:56955918:G:A | 0.496 | -0.132 | 1.422 | 4.57E-09 | rs12923459 | 0.99 | intergenic | *HERPUD1-CETP* | 3.673 | Yes |
| 16:56951244:A:G | 0.683 | 0.134 | 1.521 | 2.94E-08 | rs193695 | 0.912 | intergenic | *HERPUD1-CETP* | 0.11 | No |
| 8:20054859:G:A | 0.188 | 0.157 | 1.805 | 3.82E-08 | rs115849089 | 0.995 | intergenic | *LPL-SLC18A1* | 0.705 | No |
| 11:116792991:G:A | 0.808 | 0.151 | 1.778 | 8.26E-08 | rs662799 | 1 | upstream | *ZPR1* | 0.931 | No |
| 11:116778201:G:C | 0.781 | 0.143 | 1.701 | 1.22E-07 | rs964184 | 1 | 3_prime_UTR | *ZPR1* | 0.481 | No |
| 8:19955669:G:A | 0.252 | 0.127 | 1.613 | 6.87E-07 | rs264 | 1 | intron | *LPL* | 1.66 | Yes |
| 1:111674339:C:T | 0.213 | 0.142 | 1.812 | 7.74E-07 | rs7525578 | 1 | intron | *RAP1A* | 6.396 | Yes |
| 8:19978017:C:CA | 0.290 | 0.117 | 1.556 | 1.96E-06 | rs34940792 | 0.974 | intergenic | *LPL-SLC18A1* | 0.394 | Yes |
| 15:58432643:G:A | 0.470 | 0.107 | 1.422 | 2.00E-06 | rs8033940 | 0.998 | downstream | *LIPC-AS1* | 0.275 | Yes |
| 8:20050401:T:C | 0.158 | 0.144 | 1.938 | 2.68E-06 | rs11991231 | 1 | intergenic | *LPL-SLC18A1* | 3.405 | Yes |
| 16:56982180:G:A | 0.517 | -0.104 | 1.404 | 2.86E-06 | rs5882 | 0.972 | missense | *CETP* | 0.001 | No |
| 15:58435126:A:G | 0.799 | -0.130 | 1.756 | 2.95E-06 | rs261332 | 0.97 | intron | *LIPC* | 0.627 | No |
| 21:34349030:A:G | 0.454 | -0.106 | 1.434 | 3.15E-06 | rs12482560 | 0.998 | intergenic | *LINC00310-KCNE2* | 0.102 | Yes |
| 8:19953276:C:A | 0.162 | 0.141 | 1.930 | 4.23E-06 | rs343 | 0.93 | intron | *LPL* | 0.489 | No |
| **Lipid: LDL; N=3943** | | | | | | | | | | |
| ***ID*** | ***AF*** | ***Beta*** | ***SE*** | ***P*** | ***rsid*** | ***R^2^*** | **Consequence** | **Gene** | **CADD-v1.7** | **Novel** |
| 1:109275684:G:T | 0.708 | 0.204 | 1.555 | 1.64E-16 | rs629301 | 1 | 3_prime_UTR | *CELSR2* | 0.227 | No |
| 1:109272258:C:T | 0.168 | -0.155 | 1.887 | 2.67E-07 | rs4970834 | 0.981 | intron | *CELSR2* | 10.92 | No |
| 16:80812168:A:G | 0.201 | -0.145 | 1.781 | 3.15E-07 | rs55888375 | 0.978 | intergenic | *CDYL2-ARLNC1* | 3.004 | Yes |
| 1:109270614:TTCCCAGTCTTGGGG:T | 0.372 | 0.108 | 1.447 | 2.78E-06 | rs79668084 | 0.901 | intron | *CELSR2* | - | Yes |
| 6:24229986:C:A | 0.721 | 0.117 | 1.581 | 3.08E-06 | rs793837 | 0.983 | intron | *DCDC2* | 0.214 | Yes |
| 6:24220870:A:AGAGAGAC | 0.732 | 0.117 | 1.578 | 3.24E-06 | rs1554146303 | 0.861 | intron | *DCDC2* | 0.721 | Yes |
| **Lipid: log TG; N=3982** | | | | | | | | | | |
| ***ID*** | ***AF*** | ***Beta*** | ***SE*** | ***P*** | ***rsid*** | ***R^2^*** | **Consequence** | **Gene** | **CADD-v1.7** | **Novel** |
| 11:116778201:G:C | 0.781 | -0.297 | 1.702 | 3.73E-28 | rs964184 | 1 | 3_prime_UTR | *ZPR1* | 0.481 | No |
| 11:116792991:G:A | 0.808 | -0.278 | 1.779 | 5.48E-23 | rs662799 | 1 | upstream | *ZPR1* | 0.931 | No |
| 11:116753987:G:T | 0.696 | -0.229 | 1.534 | 3.88E-21 | rs180326 | 0.999 | intron | *BUD13* | 0.016 | No |
| 11:116752497:TA:T | 0.700 | -0.220 | 1.536 | 1.47E-19 | rs66505542 | 0.994 | intron | *BUD13* | - | Yes |
| 11:116715567:T:C | 0.767 | -0.209 | 1.665 | 2.30E-15 | rs7350481 | 1 | intergenic | *LINC02702-BUD13* | 0.933 | No |
| 11:116800289:A:G | 0.743 | -0.196 | 1.596 | 9.54E-15 | rs4938313 | 0.996 | intergenic | *APOA5-APOA4* | 0.928 | No |
| 11:116801297:T:C | 0.747 | -0.196 | 1.604 | 1.11E-14 | rs11216140 | 0.985 | intergenic | *APOA5-APOA4* | 1.148 | No |
| 11:116799960:A:C | 0.744 | -0.194 | 1.598 | 1.62E-14 | rs6589567 | 1 | intergenic | *APOA5-APOA4* | 1.11 | No |
| 11:116800760:T:C | 0.744 | -0.194 | 1.598 | 1.62E-14 | rs6589569 | 0.994 | intergenic | *APOA5-APOA4* | 1.372 | No |
| 11:116793324:G:A | 0.474 | -0.146 | 1.430 | 1.19E-10 | rs10750097 | 0.966 | upstream | *APOA5* | 4.866 | Yes |
| 11:116836622:A:G | 0.618 | -0.146 | 1.466 | 3.22E-10 | [rs7116797](https://www.ncbi.nlm.nih.gov/snp/rs7116797) | 0.963 | downstream | *APOC3* | 8.426 | No |
| 11:116738598:G:GCA | 0.243 | 0.158 | 1.655 | 1.62E-09 | rs57228226 | 0.79 | intergenic | *LINC02702-BUD13* | 0.608 | Yes |
| 11:116774559:C:T | 0.399 | -0.137 | 1.445 | 2.03E-09 | rs7118999 | 0.982 | 3_prime_UTR | *ZPR1* | 0.767 | Yes |
| 11:116815326:C:CT | 0.578 | -0.135 | 1.437 | 3.34E-09 | rs71037424 | 0.992 | intergenic | *APOA5-APOA4* | 0.413 | Yes |
| 11:116813312:T:C | 0.619 | -0.134 | 1.455 | 6.89E-09 | rs7396835 | 1 | intergenic | *APOA5-APOA4* | 1.36 | No |
| 11:116821618:C:T | 0.619 | -0.134 | 1.466 | 7.77E-09 | rs5104 | 0.99 | missense | *APOA4* | 0.023 | No |
| 11:116825064:A:G | 0.620 | -0.133 | 1.465 | 9.12E-09 | rs7111242 | 0.988 | upstream | *APOA4* | 2.332 | No |
| 11:116725458:C:T | 0.260 | -0.147 | 1.622 | 1.07E-08 | [rs180365](https://www.ncbi.nlm.nih.gov/snp/rs180365) | 0.987 | intergenic | *LINC02702-BUD13* | 3.522 | No |
| 11:116809702:T:C | 0.623 | -0.133 | 1.475 | 1.27E-08 | rs6589571 | 0.985 | intergenic | *APOA5-APOA4* | 0.298 | No |
| 11:116771421:C:T | 0.362 | -0.132 | 1.463 | 1.30E-08 | rs664059 | 0.995 | downstream | *ZPR1* | 0.406 | Yes |
| 11:116815050:C:G | 0.623 | -0.133 | 1.473 | 1.33E-08 | rs6589573 | 0.997 | intergenic | *APOA5-APOA4* | 0.147 | No |
| 11:116810847:G:A | 0.620 | -0.131 | 1.461 | 1.47E-08 | rs6589572 | 0.992 | intergenic | *APOA5-APOA4* | 0.926 | No |
| 11:116811440:G:A | 0.620 | -0.131 | 1.460 | 1.49E-08 | rs7927820 | 0.994 | intergenic | *APOA5-APOA4* | 1.671 | No |
| 11:116813448:T:C | 0.620 | -0.131 | 1.460 | 1.49E-08 | rs7396851 | 0.999 | intergenic | *APOA5-APOA4* | 0.195 | No |
| 11:116830819:T:C | 0.538 | -0.127 | 1.425 | 1.93E-08 | rs4520 | 0.924 | synonymous | *APOC3* | 2.275 | Yes |
| 11:116826628:C:T | 0.499 | -0.120 | 1.407 | 6.93E-08 | rs2071523 | 0.995 | upstream | *APOA4* | 0.096 | Yes |
| 11:116827022:C:A | 0.499 | -0.120 | 1.407 | 6.93E-08 | rs2542051 | 0.993 | upstream | *APOA4* | 0.022 | Yes |
| 11:116819407:T:C | 0.503 | -0.120 | 1.410 | 7.30E-08 | rs1263176 | 0.991 | downstream | *APOA4* | 0.035 | Yes |
| 11:116825964:C:T | 0.502 | -0.120 | 1.408 | 7.79E-08 | rs2098453 | 0.999 | upstream | *APOA4* | 0.225 | Yes |
| 11:116826171:G:T | 0.502 | -0.120 | 1.408 | 7.79E-08 | rs2727789 | 0.999 | upstream | *APOA4* | 0.926 | Yes |
| 11:116826401:C:A | 0.502 | -0.120 | 1.408 | 7.79E-08 | rs2542050 | 0.999 | upstream | *APOA4* | 1.163 | Yes |
| 11:116826822:T:C | 0.502 | -0.120 | 1.408 | 7.80E-08 | rs2071522 | 0.995 | upstream | *APOA4* | 5.435 | Yes |
| 11:116826205:T:C | 0.502 | -0.120 | 1.409 | 8.48E-08 | rs2849176 | 1 | upstream | *APOA4* | 1.896 | Yes |
| 11:116827132:G:A | 0.498 | -0.119 | 1.407 | 9.60E-08 | rs2071521 | 0.992 | upstream | *APOA4* | 5.493 | Yes |
| 11:116819996:C:T | 0.502 | -0.119 | 1.408 | 1.08E-07 | rs1263177 | 0.991 | downstream | *APOA4* | 0.264 | Yes |
| 11:116821978:G:A | 0.502 | -0.119 | 1.408 | 1.08E-07 | rs5100 | 0.993 | intron | *APOA4* | 3.276 | Yes |
| 11:116822379:G:A | 0.502 | -0.119 | 1.408 | 1.08E-07 | rs5096 | 0.991 | intron | *APOA4* | 0.012 | Yes |
| 11:116824150:G:A | 0.502 | -0.119 | 1.408 | 1.08E-07 | rs1268833 | 0.996 | upstream | *APOA4* | 1.046 | Yes |
| 11:116825327:G:A | 0.502 | -0.119 | 1.408 | 1.08E-07 | rs2216311 | 0.997 | upstream | *APOA4* | 7.539 | Yes |
| 11:116828729:C:A | 0.496 | -0.119 | 1.408 | 1.08E-07 | rs595049 | 0.981 | upstream | *APOC3* | 0.184 | Yes |
| 11:116819862:C:T | 0.501 | -0.118 | 1.408 | 1.22E-07 | rs1268354 | 0.991 | downstream | *APOA4* | 7.526 | Yes |
| 11:116829426:T:C | 0.544 | -0.117 | 1.418 | 1.77E-07 | rs2854117 | 0.945 | upstream | *APOC3* | 4.728 | Yes |
| 2:27508073:T:C | 0.815 | -0.147 | 1.809 | 2.71E-07 | [rs1260326](https://www.ncbi.nlm.nih.gov/snp/rs1260326) | 1 | missense&splice | *GCKR* | 10.99 | No |
| 11:116714271:C:T | 0.363 | -0.119 | 1.480 | 4.17E-07 | rs4938303 | 1 | intergenic | *LINC02702-BUD13* | 15.1 | No |
| 11:116718401:TA:T | 0.516 | -0.113 | 1.428 | 6.34E-07 | rs10711344 | 0.962 | intergenic | *LINC02702-BUD13* | - | Yes |
| 5:110236840:G:C | 0.383 | -0.115 | 1.453 | 6.53E-07 | rs150389557 | 0.982 | intergenic | *LINC01848-TMEM232* | 1.368 | Yes |
| 11:116701373:C:T | 0.214 | 0.135 | 1.717 | 7.13E-07 | rs1892948 | 0.956 | intergenic | *LINC02702-BUD13* | 2.702 | Yes |
| 18:77349166:G:A | 0.158 | -0.150 | 1.924 | 8.23E-07 | rs8090949 | 0.993 | intergenic | *GALR1-LINC01029* | 0.033 | Yes |
| 11:116859922:A:G | 0.732 | -0.122 | 1.591 | 1.24E-06 | rs6589574 | 0.994 | downstream | *APOA1-AS* | 0.026 | No |
| 11:116840425:C:T | 0.389 | -0.112 | 1.466 | 1.44E-06 | rs2727784 | 0.975 | upstream | *APOA1* | 2.002 | No |
| 11:117174619:T:C | 0.766 | -0.129 | 1.698 | 1.64E-06 | rs7925256 | 0.936 | upstream | *SIDT2* | 1.47 | Yes |
| 10:96232187:C:T | 0.302 | -0.113 | 1.505 | 2.09E-06 | rs75025927 | 0.987 | intron | *BLNK* | 8.087 | Yes |
| 8:20085797:A:G | 0.500 | -0.106 | 1.419 | 2.34E-06 | rs13276972 | 0.975 | intergenic | *LPL-SLC18A1* | 1.06 | Yes |
| 1:119658220:T:C | 0.621 | -0.109 | 1.462 | 2.36E-06 | rs481357 | 0.962 | intergenic | *ZNF697-PHGDH* | 0.085 | Yes |
| 2:25356402:G:C | 0.674 | 0.113 | 1.532 | 3.56E-06 | rs12713527 | 0.972 | intergenic | *DNMT3A-DTNB* | 0.016 | Yes |
| 2:27757911:C:CA | 0.167 | 0.137 | 1.880 | 4.00E-06 | rs1186060731 | 0.923 | intergenic | *LOC105374378-MRPL33* | 2.241 | Yes |
| 8:19958045:A:G | 0.299 | -0.113 | 1.547 | 4.22E-06 | rs287 | 0.985 | intron | *LPL* | 3.996 | No |
| 5:110202548:A:ATTTATT | 0.480 | -0.102 | 1.400 | 4.40E-06 | rs10681776 | 0.985 | intergenic | *LINC01848-TMEM232* | 1.111 | Yes |
| 8:23342898:A:G | 0.232 | -0.122 | 1.679 | 4.75E-06 | [rs6557654](https://www.ncbi.nlm.nih.gov/snp/rs6557654) | 0.92 | intron | *LOXL2* | 1.094 | Yes |
| 11:117307509:A:G | 0.188 | 0.131 | 1.803 | 4.81E-06 | rs558229 | 0.975 | intron | *BACE1* | 1.165 | Yes |
| **Lipid: TC; N=3982** | | | | | | | | | | |
| ***ID*** | ***AF*** | ***Beta*** | ***SE*** | ***P*** | ***rsid*** | ***R^2^*** | **Consequence** | **Gene** | **CADD-v1.7** | **Novel** |
| 1:109278685:G:T | 0.707 | 0.157 | 1.549 | 1.78E-10 | rs583104 | 0.998 | downstream | *CELSR2* | 4.52 | No |
| 19:19551411:C:T | 0.159 | -0.178 | 1.927 | 5.96E-09 | rs17216525 | 1 | downstream | *CILP2* | 1.356 | No |
| 19:19584419:C:T | 0.212 | -0.147 | 1.736 | 9.49E-08 | rs73004951 | 0.972 | intron | *PBX4* | 1.082 | Yes |
| 19:19610913:G:A | 0.201 | -0.147 | 1.767 | 1.51E-07 | rs12610185 | 1 | intron | *PBX4* | 1.891 | No |
| 19:19611167:C:T | 0.201 | -0.147 | 1.767 | 1.51E-07 | rs12610191 | 1 | intron | *PBX4* | 0.130 | No |
| 19:19612406:C:T | 0.201 | -0.147 | 1.767 | 1.51E-07 | rs10500212 | 0.999 | intron | *PBX4* | 12.22 | No |
| 19:19615213:G:A | 0.201 | -0.147 | 1.767 | 1.51E-07 | rs58847337 | 0.997 | intron | *PBX4* | 1.996 | No |
| 19:19605749:C:T | 0.202 | -0.145 | 1.762 | 2.10E-07 | rs73004966 | 0.995 | intron | *PBX4* | 0.759 | No |
| 19:19602260:A:T | 0.202 | -0.144 | 1.762 | 2.31E-07 | rs73004962 | 0.995 | intron | *PBX4* | 2.275 | No |
| 19:19609590:C:T | 0.202 | -0.144 | 1.762 | 2.31E-07 | rs57504626 | 0.999 | intron | *PBX4* | 1.843 | No |
| 19:19609979:T:G | 0.202 | -0.144 | 1.762 | 2.31E-07 | rs16996185 | 0.999 | intron | *PBX4* | 4.911 | No |
| 19:19616343:A:G | 0.205 | -0.139 | 1.753 | 5.19E-07 | rs73004975 | 0.992 | intron | *PBX4* | 1.365 | No |
| 19:19296060:T:C | 0.217 | -0.130 | 1.708 | 1.67E-06 | rs12979148 | 0.988 | intron | *SUGP1* | 4.04 | Yes |
| 10:60805285:C:T | 0.290 | 0.115 | 1.550 | 2.96E-06 | rs7093708 | 0.997 | intergenic | *CDK1-RHOBTB1* | 0.037 | Yes |
| 19:19406360:A:G | 0.200 | -0.130 | 1.774 | 3.70E-06 | rs188552254 | 0.995 | intron | *GATAD2A* | 13.96 | Yes |
| 12:13967555:A:G | 0.301 | -0.112 | 1.533 | 3.87E-06 | rs11055697 | 1 | intron | *GRIN2B* | 2.881 | Yes |
| **Lipid: TG:HDL; N=3982** | | | | | | | | | | |
| ***ID*** | ***AF*** | ***Beta*** | ***SE*** | ***P*** | ***rsid*** | ***R^2^*** | **Consequence** | **Gene** | **CADD-v1.7** | **Novel** |
| 11:116778201:G:C | 0.781 | -0.292 | 1.700 | 2.06E-27 | rs964184 | 1 | 3_prime_UTR | *ZPR1* | 0.481 | No |
| 11:116792991:G:A | 0.808 | -0.288 | 1.777 | 1.65E-24 | rs662799 | 1 | upstream | *ZPR1* | 0.931 | No |
| 11:116753987:G:T | 0.696 | -0.221 | 1.533 | 1.05E-19 | rs180326 | 0.999 | intron | *BUD13* | 0.016 | No |
| 11:116752497:TA:T | 0.700 | -0.211 | 1.534 | 3.86E-18 | rs66505542 | 0.994 | intron | *BUD13* | - | Yes |
| 11:116715567:T:C | 0.767 | -0.199 | 1.663 | 4.29E-14 | rs7350481 | 1 | intergenic | *LINC02702-BUD13* | 0.933 | No |
| 11:116801297:T:C | 0.747 | -0.188 | 1.602 | 1.20E-13 | rs11216140 | 0.985 | intergenic | *APOA5-APOA4* | 1.148 | No |
| 11:116800289:A:G | 0.743 | -0.187 | 1.595 | 1.36E-13 | rs4938313 | 0.996 | intergenic | *APOA5-APOA4* | 0.928 | No |
| 11:116799960:A:C | 0.744 | -0.185 | 1.597 | 2.53E-13 | rs6589567 | 1 | intergenic | *APOA5-APOA4* | 1.11 | No |
| 11:116800760:T:C | 0.744 | -0.185 | 1.597 | 2.53E-13 | rs6589569 | 0.994 | intergenic | *APOA5-APOA4* | 1.372 | No |
| 8:20002028:G:A | 0.284 | -0.153 | 1.576 | 9.25E-10 | rs2119690 | 0.999 | intergenic | *LPL-SLC18A1* | 2.963 | No |
| 11:116793324:G:A | 0.474 | -0.138 | 1.429 | 1.17E-09 | rs10750097 | 0.966 | upstream | *APOA5* | 4.866 | Yes |
| 8:20054859:G:A | 0.188 | -0.168 | 1.804 | 4.46E-09 | rs115849089 | 0.995 | intergenic | *LPL-SLC18A1* | 0.705 | No |
| 11:116738598:G:GCA | 0.243 | 0.153 | 1.653 | 5.63E-09 | rs57228226 | 0.79 | intergenic | *LINC02702-BUD13* | 0.608 | Yes |
| 11:116836968:A:G | 0.656 | -0.130 | 1.500 | 4.44E-08 | rs2070665 | 0.942 | downstream | *APOC3* | 0.285 | Yes |
| 8:20041650:T:C | 0.151 | -0.169 | 1.966 | 5.99E-08 | rs76259755 | 0.986 | intergenic | *LPL-SLC18A1* | 0.457 | No |
| 11:116774559:C:T | 0.399 | -0.123 | 1.444 | 8.45E-08 | rs7118999 | 0.982 | 3_prime_UTR | *ZPR1* | 0.767 | Yes |
| 8:20085797:A:G | 0.500 | -0.118 | 1.418 | 1.38E-07 | rs13276972 | 0.975 | intergenic | *LPL-SLC18A1* | 1.06 | Yes |
| 5:110236840:G:C | 0.383 | -0.116 | 1.452 | 4.77E-07 | rs150389557 | 0.982 | intergenic | *LINC01848-TMEM232* | 1.368 | Yes |
| 11:116804578:A:T | 0.622 | -0.116 | 1.467 | 6.24E-07 | rs6589570 | 0.955 | intergenic | *APOA5-APOA4* | 0.276 | No |
| 11:116934488:T:C | 0.635 | -0.115 | 1.471 | 7.88E-07 | rs562179 | 0.987 | intron | *SIK3* | 1.297 | Yes |
| 8:20083937:C:T | 0.324 | -0.118 | 1.514 | 8.44E-07 | rs6989064 | 0.968 | intergenic | *LPL-SLC18A1* | 0.878 | No |
| 11:116701373:C:T | 0.214 | 0.134 | 1.716 | 8.75E-07 | rs1892948 | 0.956 | intergenic | *LINC02702-BUD13* | 2.702 | Yes |
| 11:116821618:C:T | 0.619 | -0.114 | 1.464 | 8.76E-07 | rs5104 | 0.99 | missense | *APOA4* | 0.023 | No |
| 11:116825064:A:G | 0.620 | -0.114 | 1.464 | 9.78E-07 | rs7111242 | 0.988 | upstream | *APOA4* | 2.332 | No |
| 11:116859922:A:G | 0.732 | -0.123 | 1.589 | 1.00E-06 | rs6589574 | 0.994 | downstream | *APOA1-AS* | 0.026 | No |
| 11:116771421:C:T | 0.362 | -0.113 | 1.461 | 1.09E-06 | rs664059 | 0.995 | downstream | *ZPR1* | 0.406 | Yes |
| 11:116830819:T:C | 0.538 | -0.109 | 1.423 | 1.35E-06 | rs4520 | 0.924 | synonymous | *APOC3* | 2.275 | Yes |
| 11:116809702:T:C | 0.623 | -0.111 | 1.473 | 1.82E-06 | rs6589571 | 0.985 | intergenic | *APOA5-APOA4* | 0.298 | No |
| 11:116718401:TA:T | 0.516 | -0.107 | 1.426 | 2.02E-06 | rs10711344 | 0.962 | intergenic | *LINC02702-BUD13* | - | Yes |
| 11:116813312:T:C | 0.619 | -0.109 | 1.454 | 2.06E-06 | rs7396835 | 1 | intergenic | *APOA5-APOA4* | 1.36 | No |
| 11:116815050:C:G | 0.623 | -0.110 | 1.471 | 2.28E-06 | rs6589573 | 0.997 | intergenic | *APOA5-APOA4* | 0.147 | No |
| 11:116810847:G:A | 0.620 | -0.108 | 1.459 | 3.07E-06 | rs6589572 | 0.992 | intergenic | *APOA5-APOA4* | 0.926 | No |
| 2:45749281:A:G | 0.837 | -0.144 | 1.958 | 3.27E-06 | rs11125030 | 1 | intron | *PRKCE* | 12.84 | Yes |
| 11:116811440:G:A | 0.620 | -0.107 | 1.459 | 3.38E-06 | rs7927820 | 0.994 | intergenic | *APOA5-APOA4* | 1.671 | No |
| 11:116813448:T:C | 0.620 | -0.107 | 1.459 | 3.38E-06 | rs7396851 | 0.999 | intergenic | *APOA5-APOA4* | 0.195 | No |
| 5:110202548:A:ATTTATT | 0.480 | -0.103 | 1.399 | 3.64E-06 | rs10681776 | 0.985 | intergenic | *LINC01848-TMEM232* | 1.111 | Yes |
| ***Lipid: VAI; N=3886*** | | | | | | | | | | |
| ***ID*** | ***AF*** | ***Beta*** | ***SE*** | ***P*** | ***rsid*** | ***R^2^*** | **Consequence** | ***Gene*** | **CADD-v1.7** | **Novel** |
| 11:116778201:G:C | 0.780 | -0.287 | 1.704 | 7.67E-26 | rs964184 | 1 | 3_prime_UTR | *ZPR1* | 0.481 | No |
| 11:116792991:G:A | 0.807 | -0.286 | 1.780 | 1.24E-23 | rs662799 | 1 | upstream | *ZPR1* | 0.931 | No |
| 11:116753987:G:T | 0.695 | -0.217 | 1.533 | 9.67E-19 | rs180326 | 0.999 | intron | *BUD13* | 0.016 | No |
| 11:116752497:TA:T | 0.700 | -0.212 | 1.535 | 8.82E-18 | rs66505542 | 0.994 | intron | *BUD13* | - | Yes |
| 11:116801297:T:C | 0.746 | -0.197 | 1.603 | 1.74E-14 | rs11216140 | 0.985 | intergenic | *APOA5-APOA4* | 1.148 | No |
| 11:116800289:A:G | 0.743 | -0.195 | 1.595 | 2.93E-14 | rs4938313 | 0.996 | intergenic | *APOA5-APOA4* | 0.928 | No |
| 11:116799960:A:C | 0.743 | -0.193 | 1.597 | 4.83E-14 | rs6589567 | 1 | intergenic | *APOA5-APOA4* | 1.11 | No |
| 11:116800760:T:C | 0.743 | -0.193 | 1.597 | 4.83E-14 | rs6589569 | 0.994 | intergenic | *APOA5-APOA4* | 1.372 | No |
| 11:116715567:T:C | 0.767 | -0.194 | 1.668 | 3.69E-13 | rs7350481 | 1 | intergenic | *LINC02702-BUD13* | 0.933 | No |
| 8:20002028:G:A | 0.284 | -0.145 | 1.580 | 1.09E-08 | rs2119690 | 0.999 | intergenic | *LPL-SLC18A1* | 2.963 | No |
| 11:116738598:G:GCA | 0.243 | 0.151 | 1.659 | 1.27E-08 | rs57228226 | 0.79 | intergenic | *LINC02702-BUD13* | 0.608 | Yes |
| 11:116836968:A:G | 0.655 | -0.136 | 1.501 | 1.57E-08 | rs2070665 | 0.942 | downstream | *APOC3* | 0.285 | No |
| 11:116804578:A:T | 0.620 | -0.126 | 1.467 | 9.12E-08 | rs6589570 | 0.955 | intergenic | *APOA5-APOA4* | 0.276 | No |
| 8:20054859:G:A | 0.188 | -0.154 | 1.809 | 1.07E-07 | rs115849089 | 0.995 | intergenic | *LPL-SLC18A1* | 0.705 | Yes |
| 11:116821618:C:T | 0.618 | -0.123 | 1.465 | 1.55E-07 | rs5104 | 0.99 | missense | *APOA4* | 0.023 | No |
| 11:116793324:G:A | 0.473 | -0.120 | 1.432 | 1.80E-07 | rs10750097 | 0.966 | upstream | *APOA5* | 4.866 | Yes |
| 11:116825064:A:G | 0.618 | -0.123 | 1.465 | 1.80E-07 | rs7111242 | 0.988 | upstream | *APOA4* | 2.332 | No |
| 11:116809702:T:C | 0.622 | -0.123 | 1.474 | 2.14E-07 | rs6589571 | 0.985 | intergenic | *APOA5-APOA4* | 0.298 | No |
| 11:116813312:T:C | 0.617 | -0.120 | 1.455 | 3.07E-07 | rs7396835 | 1 | intergenic | *APOA5-APOA4* | 1.36 | No |
| 11:116815050:C:G | 0.621 | -0.120 | 1.472 | 3.69E-07 | rs6589573 | 0.997 | intergenic | *APOA5-APOA4* | 0.147 | No |
| 11:116810847:G:A | 0.618 | -0.118 | 1.461 | 4.79E-07 | rs6589572 | 0.992 | intergenic | *APOA5-APOA4* | 0.926 | No |
| 11:116811440:G:A | 0.618 | -0.118 | 1.461 | 5.32E-07 | rs7927820 | 0.994 | intergenic | *APOA5-APOA4* | 1.671 | No |
| 11:116813448:T:C | 0.618 | -0.118 | 1.461 | 5.32E-07 | rs7396851 | 0.999 | intergenic | *APOA5-APOA4* | 0.195 | No |
| 8:20083937:C:T | 0.323 | -0.122 | 1.519 | 5.46E-07 | rs6989064 | 0.968 | intergenic | *LPL-SLC18A1* | 0.878 | No |
| 11:116701373:C:T | 0.214 | 0.138 | 1.718 | 5.70E-07 | rs1892948 | 0.956 | intergenic | *LINC02702-BUD13* | 2.702 | Yes |
| 8:20085797:A:G | 0.500 | -0.114 | 1.422 | 5.98E-07 | rs13276972 | 0.975 | intergenic | *LPL-SLC18A1* | 1.06 | Yes |
| 11:116830819:T:C | 0.537 | -0.112 | 1.426 | 9.04E-07 | rs4520 | 0.924 | synonymous | *APOC3* | 2.275 | Yes |
| 11:116826822:T:C | 0.501 | -0.109 | 1.407 | 1.34E-06 | rs2071522 | 0.995 | upstream | *APOA4* | 5.435 | Yes |
| 11:116825964:C:T | 0.501 | -0.109 | 1.407 | 1.41E-06 | rs2098453 | 0.999 | upstream | *APOA4* | 0.225 | Yes |
| 11:116826171:G:T | 0.501 | -0.109 | 1.407 | 1.41E-06 | rs2727789 | 0.999 | upstream | *APOA4* | 0.926 | Yes |
| 11:116826401:C:A | 0.501 | -0.109 | 1.407 | 1.41E-06 | rs2542050 | 0.999 | upstream | *APOA4* | 1.163 | Yes |
| 11:116826205:T:C | 0.501 | -0.109 | 1.408 | 1.54E-06 | rs2849176 | 1 | upstream | *APOA4* | 1.896 | Yes |
| 11:116819407:T:C | 0.502 | -0.109 | 1.409 | 1.55E-06 | rs1263176 | 0.991 | downstream | *APOA4* | 0.035 | Yes |
| 5:110236840:G:C | 0.383 | -0.112 | 1.454 | 1.56E-06 | rs150389557 | 0.982 | intergenic | *LINC01848-TMEM232* | 1.368 | Yes |
| 11:116826628:C:T | 0.498 | -0.108 | 1.406 | 1.59E-06 | rs2071523 | 0.995 | upstream | *APOA4* | 0.096 | Yes |
| 11:116827022:C:A | 0.498 | -0.108 | 1.406 | 1.59E-06 | rs2542051 | 0.993 | upstream | *APOA4* | 0.022 | Yes |
| 11:116774559:C:T | 0.398 | -0.111 | 1.448 | 1.73E-06 | rs7118999 | 0.982 | 3_prime_UTR | *ZPR1* | 0.767 | Yes |
| 8:23340817:A:G | 0.232 | -0.129 | 1.684 | 1.75E-06 | rs4301460 | 0.908 | intron | *LOXL2* | 0.181 | Yes |
| 11:116934488:T:C | 0.634 | -0.113 | 1.473 | 1.77E-06 | rs562179 | 0.987 | intron | *SIK3* | 1.297 | Yes |
| 11:116969045:T:C | 0.732 | -0.121 | 1.584 | 1.81E-06 | rs1241657 | 0.997 | intron | *SIK3* | 2.239 | No |
| 11:116828729:C:A | 0.495 | -0.108 | 1.406 | 1.85E-06 | rs595049 | 0.981 | upstream | *APOC3* | 0.184 | Yes |
| 1:226559961:A:G | 0.643 | 0.112 | 1.472 | 2.03E-06 | rs10916008 | 0.995 | intron | *STUM* | 1.502 | Yes |
| 11:116819996:C:T | 0.501 | -0.107 | 1.406 | 2.24E-06 | rs1263177 | 0.991 | downstream | *APOA4* | 0.264 | Yes |
| 11:116821978:G:A | 0.501 | -0.107 | 1.406 | 2.24E-06 | rs5100 | 0.993 | intron | *APOA4* | 3.276 | Yes |
| 11:116822379:G:A | 0.501 | -0.107 | 1.406 | 2.24E-06 | rs5096 | 0.991 | intron | *APOA4* | 0.012 | Yes |
| 11:116824150:G:A | 0.501 | -0.107 | 1.406 | 2.24E-06 | rs1268833 | 0.996 | upstream | *APOA4* | 1.046 | Yes |
| 11:116825327:G:A | 0.501 | -0.107 | 1.406 | 2.24E-06 | rs2216311 | 0.997 | upstream | *APOA4* | 7.539 | Yes |
| 11:116819862:C:T | 0.500 | -0.106 | 1.407 | 2.46E-06 | rs1268354 | 0.991 | downstream | *APOA4* | 7.526 | Yes |
| 11:116827132:G:A | 0.498 | -0.106 | 1.406 | 2.51E-06 | rs2071521 | 0.992 | upstream | *APOA4* | 5.493 | Yes |
| 3:71449831:C:T | 0.370 | 0.111 | 1.479 | 2.86E-06 | rs12492180 | 0.992 | intron | *FOXP1* | 0.556 | Yes |
| 8:20050401:T:C | 0.159 | -0.145 | 1.941 | 3.18E-06 | rs11991231 | 1 | intergenic | *LPL-SLC18A1* | 3.405 | No |
| 11:116829426:T:C | 0.542 | -0.105 | 1.416 | 3.49E-06 | rs2854117 | 0.945 | upstream | *APOC3* | 4.728 | Yes |
| 8:19987865:C:T | 0.152 | -0.146 | 1.970 | 3.89E-06 | rs7841189 | 1 | intergenic | *LPL-SLC18A1* | 1.899 | No |
| 4:132277270:T:C | 0.558 | -0.105 | 1.420 | 4.19E-06 | rs13113408 | 0.925 | intergenic | *SNHG27-LINC01256* | 1.395 | Yes |
| 16:56973534:T:G | 0.240 | 0.122 | 1.659 | 4.91E-06 | rs11076176 | 0.904 | intron | *CETP* | 2.296 | No |
| 11:116768976:C:T | 0.419 | -0.106 | 1.442 | 4.91E-06 | rs1268353 | 0.996 | downstream | *ZPR1* | 0.791 | Yes |
| **Lipid: Visceral Fat; N=4403** | | | | | | | | | | |
| **ID** | **AF** | **Beta** | **SE** | **P** | **rsid** | **R2** | **Consequence** | **Gene** | **CADD-v1.7** | **Novel** |
| 7:22011848:T:C | 0.329 | -0.120 | 1.511 | 1.46E-07 | rs6970233 | 1 | intergenic | *CDCA7L-RAPGEF5* | 0.408 | Yes |
| 7:22019322:C:T | 0.290 | -0.120 | 1.581 | 4.83E-07 | rs6960041 | 0.809 | intergenic | *CDCA7L-RAPGEF5* | 0.151 | Yes |
| 3:7404746:T:C | 0.767 | -0.125 | 1.676 | 6.80E-07 | rs712771 | 0.98 | intron | *GRM7* | 0.985 | Yes |
| 3:59344012:A:G | 0.151 | -0.146 | 1.974 | 9.58E-07 | rs11130700 | 0.986 | intergenic | *C3orf67-LOC339902* | 1.445 | Yes |
| 12:41670435:G:A | 0.539 | -0.106 | 1.433 | 9.96E-07 | rs11181087 | 0.974 | intergenic | *PDZRN4-LINC02400* | 2.839 | Yes |
| 19:28518034:T:C | 0.354 | -0.106 | 1.469 | 1.77E-06 | rs12150955 | 0.965 | intron | *LOC100420587* | 4.254 | Yes |
| **Metabolic Syndrome; N=3792** | | | | | | | | | | |
| ***ID*** | ***AF*** | ***Beta*** | ***SE*** | ***P*** | ***rsid*** | ***R2*** | ***Consequence*** | ***Gene*** | ***CADD-v1.7*** | **Novel** |
| 11:116778201:G:C | 0.781 | -0.337 | 0.060 | 2.24E-08 | rs964184 | 1 | 3_prime_UTR | *ZPR1* | 0.481 | No |
| 2:25051639:T:TG | 0.005 | 1.678 | 0.319 | 6.03E-08 | rs1417213158 | 0.857 | intron | *EFR3B* | 0.629 | Yes |
| 11:116792991:G:A | 0.808 | -0.316 | 0.063 | 4.68E-07 | rs662799 | 1 | upstream | *ZPR1* | 0.931 | No |
| 22:30785583:AC:A | 0.147 | 0.346 | 0.069 | 5.38E-07 | rs368121234 | 0.97 | intron | *OSBP2* | - | Yes |
| 4:45325954:G:A | 0.002 | 3.436 | 0.696 | 6.38E-07 | rs544377077 | 0.935 | intergenic | *GNPDA2-GABRG1* | 2.674 | Yes |
| 4:97664735:C:T | 0.014 | 0.964 | 0.203 | 1.54E-06 | rs575386659 | 0.809 | intron | *STPG2* | 2.319 | Yes |
| 7:29895770:C:T | 0.640 | 0.263 | 0.055 | 1.55E-06 | rs174992 | 0.955 | intron | *WIPF3* | 1.643 | Yes |
| 1:118241562:A:T | 0.008 | 1.231 | 0.261 | 1.77E-06 | rs532403359 | 0.71 | intergenic | *SPAG17-TBX15* | 0.026 | Yes |
| 6:116152568:C:G | 0.012 | 1.003 | 0.212 | 1.77E-06 | rs576718437 | 0.742 | intron | *NT5DC1* | 0.647 | Yes |
| 2:15741271:T:G | 0.005 | 1.476 | 0.316 | 2.11E-06 | rs535309843 | 0.788 | intergenic | *LINC01804-MYCNUT* | 0.249 | Yes |
| 3:20333887:T:C | 0.005 | 1.464 | 0.315 | 2.56E-06 | rs571652970 | 0.752 | intergenic | *SGO1-LOC101927829* | 5.844 | Yes |
| 11:116753987:G:T | 0.696 | -0.259 | 0.055 | 2.67E-06 | rs180326 | 0.999 | intron | *BUD13* | 0.016 | No |
| 2:6529048:C:T | 0.022 | 0.751 | 0.161 | 2.82E-06 | rs12619231 | 0.917 | intergenic | *LINC01247-LINC01246* | 1.512 | Yes |
| 20:3722958:A:C | 0.005 | 1.499 | 0.325 | 3.41E-06 | rs4815599 | 0.897 | intergenic | *SIGLEC1-HSPA12B* | 0.745 | Yes |
| 20:60395491:A:G | 0.038 | 0.586 | 0.127 | 3.65E-06 | rs185970002 | 0.821 | intergenic | *MIR646HG-LOC101928048* | 0.135 | Yes |
| 15:91223729:C:T | 0.014 | 0.883 | 0.192 | 3.74E-06 | rs79872252 | 0.739 | intron | *SV2B* | 9.936 | Yes |
| 12:78072406:A:G | 0.004 | 1.705 | 0.377 | 3.78E-06 | rs1333465996 | 0.862 | intron | *NAV3* | 3.707 | Yes |
| 22:30705659:T:C | 0.130 | 0.335 | 0.073 | 3.92E-06 | rs9606766 | 0.99 | intron | *OSBP2* | 6.273 | Yes |
| 14:69265210:G:C | 0.207 | 0.286 | 0.062 | 4.20E-06 | rs72720292 | 0.997 | intron | *GALNT16* | 7.616 | Yes |
| 2:24590452:G:T | 0.006 | 1.332 | 0.297 | 4.31E-06 | rs55681630 | 0.999 | intron | *NCOA1* | 2.662 | Yes |
| 20:40057566:T:C | 0.856 | -0.333 | 0.073 | 4.36E-06 | rs2142595 | 0.849 | intergenic | *LINC01370-MAFB* | 1.344 | Yes |

Genome-wide significant (P<5*10^-8^) are also stated in Table 1

Table S5:eQTL colocalization results

| **Trait** | **ID_INDEX** | **INDEX**  **rsid** | **INDEX Novel?** | **eQTL** | **eQTL**  **rsid** | **Distance of eQTL from INDEX (bp)** | **LD r^2^** | **Gene** | **Tissue** | **PP.H0.abf** | **PP.H1.abf** | **PP.H2.abf** | **PP.H3.abf** | **PP.H4.abf** |
| --- | --- | --- | --- | --- | --- | --- | --- | --- | --- | --- | --- | --- | --- | --- |
| TC | 1:109278685:G:T | rs583104 | No | 1:109279521:G:A | rs1277930 | 836 | 0.98 | PSRC1 | Brain Cortex | 0.00% | 0.00% | 0.00% | 0.00% | 100.00% |
| TC | 1:109278685:G:T | rs583104 | No | 1:109278685:G:T | rs583104 | 0 | 1.00 | PSRC1 | Brain Frontal Cortex_BA9 | 0.00% | 0.35% | 0.00% | 0.00% | 99.65% |
| TC | 1:109278685:G:T | rs583104 | No | 1:109278889:T:G | rs602633 | 204 | 0.99 | PSRC1 | Brain Putamen basal ganglia | 0.00% | 0.00% | 0.00% | 0.00% | 100.00% |
| TC | 1:109278685:G:T | rs583104 | No | 1:109274570:A:G | rs7528419 | 4115 | 0.98 | PSRC1 | Adipose Subcutaneous | 0.00% | 0.00% | 0.00% | 0.00% | 100.00% |

PP.H4.abf: posterior probability of a shared causal variant

Table S9:Collapsing categories for gene based tests

| **Coding Categories** | **PTV Categories** |
| --- | --- |
| missense | missense |
| missense&splice | missense&splice |
| 5_prime_UTR_premature_start_codon_gain | 5_prime_UTR_premature_start_codon_gain |
| start_lost | start_lost |
| start_retained | stop_gained |
| stop_gained | stop_lost |
| stop_lost | initiator_codon |
| stop_retained | initiator_codon&splice |
| initiator_codon | frameshift |
| initiator_codon&splice | frameshift&splice_acceptor&splice&intron |
| synonymous | frameshift&splice_donor&splice&intron |
| frameshift | frameshift&splice |
| frameshift&splice_acceptor&splice&intron | frameshift&start_lost |
| frameshift&splice_donor&splice&intron | frameshift&stop_gained |
| frameshift&splice | frameshift&stop_gained&splice |
| frameshift&start_lost | conservative_inframe_deletion |
| frameshift&stop_gained, | conservative_inframe_deletion&splice |
| frameshift&stop_gained&splice | conservative_inframe_insertion |
| frameshift&stop_lost | disruptive_inframe_deletion |
| frameshift&stop_lost&splice | disruptive_inframe_deletion&splice |
| conservative_inframe_deletion | disruptive_inframe_insertion |
| conservative_inframe_deletion&splice | splice_acceptor&intron_varian |
| conservative_inframe_insertion | splice_acceptor&splice_donor&intron |
| disruptive_inframe_deletion | splice_acceptor&splice&intron |
| disruptive_inframe_deletion&splice | splice_donor&intron |
| disruptive_inframe_insertion | splice_donor&splice&intron |
| 3_prime_UTR | splice&intron |
| 5_prime_UTR | start_lost&splice |
|  | stop_gained&splice |
|  | stop_lost&splice |

Table S10**:** Genome-wide significant Bonferroni-corrected gene-based association results

| **Lipid: TC; N=3982** | | | | | | |
| --- | --- | --- | --- | --- | --- | --- |
| **Gene** | | **CHISQ** | | **LOG10P** | **Collapse** | **Set** |
| *TM6SF2* | | 37.1725 | | 8.96606 | Common | Coding |
| *TM6SF2* | | 41.3518 | | 9.89567 | Common | PTV |
| *CFTR* | | 29.1401 | | 7.1718 | Rare | PTV |
| **Lipid: log TG; N=3982** | | | | | | |
| **Gene** | **CHISQ** | | **LOG10P** | | **Collapse** | **Set** |
| *TM6SF2* | 33.4043 | | 8.12577 | | Common | PTV |
| **Lipid: VAI; N=3886** | | | | | | |
| **Gene** | **CHISQ** | | **LOG10P** | | **Collapse** | **Set** |
| *BACE1* | 30.317 | | 7.43545 | | Low frequency | Coding |
| *TM6SF2* | 26.4666 | | 7.43613 | | Common | Coding |
| *TM6SF2* | 29.0662 | | 7.15524 | | Common | PTV |

Table S11: Sub-genome wide significant (p<10^-5^) gene-based association results for cognitive phenotypes

| **G-factor; N=2109** | | | | |
| --- | --- | --- | --- | --- |
| **Gene** | **CHISQ** | **LOG10P** | **Collapse** | **Set** |
| *MAF* | 20.016 | 5.11466 | Rare | Coding |
| **COGNITO Attention:** **Auditory attention; N=3385** | | | | |
| **Gene** | **CHISQ** | **LOG10P** | **Collapse** | **Set** |
| *H2BC14* | 22.1781 | 5.60469 | Rare | Coding |
| **COGNITO Attention: Visual attention; N=3284** | | | | |
| **Gene** | **CHISQ** | **LOG10P** | **Collapse** | **Set** |
| *TNNI3* | 20.2242 | 5.16194 | Rare | Coding |
| **COGNITO Attention: Mean Reaction Time; N=3600** | | | | |
| **Gene** | **CHISQ** | **LOG10P** | **Collapse** | **Set** |
| *MVB12B* | 23.0902 | 5.81086 | Low | PTV |
| *SURF2* | 20.8425 | 5.30221 | Low | PTV |
| **COGNITO Memory: Delayed Recall; N=4015** | | | | |
| **Gene** | **CHISQ** | **LOG10P** | **Collapse** | **Set** |
| *AMIGO1* | 20.3558 | 5.1918 | Common | Coding |
| *CPZ* | 20.6969 | 5.26919 | Rare | Coding |
| **COGNITO Visuospatial: Stroop Test; N=1955** | | | | |
| **Gene** | **CHISQ** | **LOG10P** | **Collapse** | **Set** |
| *ZNF500* | 22.5203 | 5.68208 | Rare | Coding |
| **Executive functioning: TMT B-A; N=1617** | | | | |
| **Gene** | **CHISQ** | **LOG10P** | **Collapse** | **Set** |
| *CPT2* | 20.3867 | 5.19882 | Rare | Coding |
| *MELTF* | 23.1162 | 5.81674 | Rare | Coding |
| *SLC26A4* | 20.6136 | 5.2503 | Rare | Coding |
| *PNP* | 20.8431 | 5.30235 | Rare | Coding |
| *APP* | 20.2229 | 5.16163 | Rare | Coding |
| **Executive functioning: Naming association (Semantic association) ; N=3889** | | | | |
| **Gene** | **CHISQ** | **LOG10P** | **Collapse** | **Set** |
| *FAM47E-STBD1* | 19.7699 | 5.05874 | Low | Coding |
| **COGNITO Language: Phonetic; N=3943** | | | | |
| **Gene** | **CHISQ** | **LOG10P** | **Collapse** | **Set** |
| *KCTD20* | 19.741 | 5.05219 | Rare | PTV |
| *NEDD4* | 20.8131 | 5.29554 | Rare | PTV |
| **COGNITO Language: Vocabulary; N=1272** | | | | |
| **Gene** | **CHISQ** | **LOG10P** | **Collapse** | **Set** |
| *CHPT1* | 20.5417 | 5.23399 | Rare | PTV |

Table S12: Sub-genome wide significant (p<10^-5^) gene-based association results for cardiometabolic phenotypes

| **Lipid: HDL-C; N=3982** | | | | |
| --- | --- | --- | --- | --- |
| **Gene** | **CHISQ** | **LOG10P** | **Collapse** | **Set** |
| *CETP* | 19.8792 | 5.08359 | Common | Coding |
| *DGCR8* | 26.6003 | 6.60169 | Rare | Coding |
| *CFTR* | 20.2945 | 5.17789 | Rare | PTV |
| *LPL* | 21.0415 | 5.34733 | Common | PTV |
| *CETP* | 23.3084 | 5.86014 | Common | PTV |
| **Lipid: log TG; N=3982** | | | | |
| **Gene** | **CHISQ** | **LOG10P** | **Collapse** | **Set** |
| *GCKR* | 23.5005 | 5.90349 | Common | Coding |
| *BAZ1B* | 20.4232 | 5.2071 | Common | Coding |
| *FZD9* | 20.9332 | 5.32278 | Common | Coding |
| *UBR5* | 19.6778 | 5.03783 | Low | Coding |
| *APOA5* | 27.9627 | 6.90771 | Low | Coding |
| *BACE1* | 22.0509 | 5.57591 | Low | Coding |
| *BUD13* | 23.2336 | 5.84325 | Low | Coding |
| *ZPR1* | 25.3348 | 6.31702 | Low | Coding |
| *TM6SF2* | 27.1959 | 6.73554 | Common | Coding |
| *GCKR* | 24.8707 | 6.2125 | Common | PTV |
| *BAZ1B* | 21.4622 | 5.44264 | Common | PTV |
| *APOA5* | 27.761 | 6.86245 | Low | PTV |
| *BUD13* | 19.9241 | 5.09379 | Low | PTV |
| *ZPR1* | 24.3208 | 6.08857 | Low | PTV |
| *TM6SF2* | 33.4043 | 8.12577 | Common | PTV |
| **Lipid: TC; N=3982** | | | | |
| **Gene** | **CHISQ** | **LOG10P** | **Collapse** | **Set** |
| *AKR1A1* | 20.5776 | 5.24214 | Low | Coding |
| *RHEX* | 20.6103 | 5.24954 | Rare | Coding |
| *UBR5* | 23.1594 | 5.82648 | Low | Coding |
| *SMPD1* | 21.9007 | 5.54192 | Rare | Coding |
| *APOE* | 20.5021 | 5.225 | Low | Coding |
| *TM6SF2* | 37.1725 | 8.96606 | Common | Coding |
| *CFTR* | 29.1401 | 7.1718 | Rare | PTV |
| *HOXA5* | 19.6953 | 5.0418 | Rare | PTV |
| *MRE11* | 25.6638 | 6.39107 | Rare | PTV |
| *TM6SF2* | 41.3518 | 9.89567 | Common | PTV |
| *SDC4* | 22.5446 | 5.68758 | Low | PTV |
| **Insulin Resistance marker: TG:HDL; N=3982** | | | | |
| **Gene** | **CHISQ** | **LOG10P** | **Collapse** | **Set** |
| *LPL* | 19.9886 | 5.10844 | Common | Coding |
| *SKIDA1* | 20.9147 | 5.31857 | Rare | Coding |
| *BACE1* | 24.6675 | 6.16672 | Low | Coding |
| *TM6SF2* | 25.2558 | 6.29923 | Common | Coding |
| *LPL* | 21.5981 | 5.47343 | Common | PTV |
| *NXNL2* | 24.398 | 6.10596 | Common | PTV |
| *TM6SF2* | 27.3806 | 6.77703 | Common | PTV |
| **Lipid: VAI; N=3886** | | | | |
| **Gene** | **CHISQ** | **LOG10P** | **Collapse** | **Set** |
| *LPL* | 21.1361 | 5.36876 | Common | Coding |
| *BACE1* | 30.317 | 7.43545 | Low | Coding |
| *TM6SF2* | 26.4666 | 7.43613 | Common | Coding |
| *NXNL2* | 26.59 | 6.59938 | Common | PTV |
| *TM6SF2* | 29.0662 | 7.15524 | Common | PTV |
| **Lipid: Visceral Fat; N=4403** | | | | |
| **Gene** | **CHISQ** | **LOG10P** | **Collapse** | **Set** |
| *GLP1R* | 24.393 | 6.10484 | Rare | PTV |
| **Metabolic Syndrome; N=3792** | | | | |
| **Gene** | **CHISQ** | **LOG10P** | **Collapse** | **Set** |
| *SCRN1* | 19.7421 | 5.05244 | Common | PTV |

Genome-wide significant results are also stated in Table S10

Table S14:Mendelian randomization heterogeneity results: Inverse variance weighted (multiplicative random effects) causal effects

| **outcome** | **exposure** | **Q** | **Q_df** | **Q_pval** |
| --- | --- | --- | --- | --- |
| Auditory_scaled | HDL | 0.0024 | 12 | 1 |
| Auditory_scaled | logTG | 0.0013 | 11 | 1 |
| Auditory_scaled | TG_HDL | 0.0011 | 10 | 1 |
| Auditory_scaled | VAI | 0.0002 | 8 | 1 |
| Comprehension_scaled | HDL | 0.0026 | 12 | 1 |
| Comprehension_scaled | logTG | 0.0022 | 11 | 1 |
| Comprehension_scaled | TG_HDL | 0.0030 | 10 | 1 |
| Comprehension_scaled | VAI | 0.0014 | 8 | 1 |
| DelayedRecall_scaled | HDL | 0.0023 | 12 | 1 |
| DelayedRecall_scaled | logTG | 0.0009 | 11 | 1 |
| DelayedRecall_scaled | TG_HDL | 0.0007 | 10 | 1 |
| DelayedRecall_scaled | VAI | 0.0004 | 8 | 1 |
| Geo_Fig_scaled | HDL | 0.0023 | 12 | 1 |
| Geo_Fig_scaled | logTG | 0.0014 | 11 | 1 |
| Geo_Fig_scaled | TG_HDL | 0.0005 | 10 | 1 |
| Geo_Fig_scaled | VAI | 0.0003 | 8 | 1 |
| ImmediateRecall_scaled | HDL | 0.0014 | 12 | 1 |
| ImmediateRecall_scaled | logTG | 0.0009 | 11 | 1 |
| ImmediateRecall_scaled | TG_HDL | 0.0008 | 10 | 1 |
| ImmediateRecall_scaled | VAI | 0.0003 | 8 | 1 |
| MRT | HDL | 0.0035 | 12 | 1 |
| MRT | logTG | 0.0004 | 11 | 1 |
| MRT | TG_HDL | 0.0004 | 10 | 1 |
| MRT | VAI | 0.0001 | 8 | 1 |
| NameFaceAssocFace_scaled | HDL | 0.0027 | 12 | 1 |
| NameFaceAssocFace_scaled | logTG | 0.0006 | 11 | 1 |
| NameFaceAssocFace_scaled | TG_HDL | 0.0007 | 10 | 1 |
| NameFaceAssocFace_scaled | VAI | 0.0005 | 8 | 1 |
| NameRecog_scaled | HDL | 0.0023 | 12 | 1 |
| NameRecog_scaled | logTG | 0.0006 | 11 | 1 |
| NameRecog_scaled | TG_HDL | 0.0005 | 10 | 1 |
| NameRecog_scaled | VAI | 0.0004 | 8 | 1 |
| Naming_Assoc_scaled | HDL | 0.0025 | 12 | 1 |
| Naming_Assoc_scaled | logTG | 0.0004 | 11 | 1 |
| Naming_Assoc_scaled | TG_HDL | 0.0004 | 10 | 1 |
| Naming_Assoc_scaled | VAI | 0.0002 | 8 | 1 |
| Phonetic | HDL | 0.0019 | 12 | 1 |
| Phonetic | logTG | 0.0006 | 11 | 1 |
| Phonetic | TG_HDL | 0.0007 | 10 | 1 |
| Phonetic | VAI | 0.0001 | 8 | 1 |
| Reading_scaled | HDL | 0.0021 | 12 | 1 |
| Reading_scaled | logTG | 0.0008 | 11 | 1 |
| Reading_scaled | TG_HDL | 0.0002 | 10 | 1 |
| Reading_scaled | VAI | 0.0002 | 8 | 1 |
| Semantic | HDL | 0.0009 | 12 | 1 |
| Semantic | logTG | 0.0003 | 11 | 1 |
| Semantic | TG_HDL | 0.0003 | 10 | 1 |
| Semantic | VAI | 0.0003 | 8 | 1 |
| Span | HDL | 0.0007 | 12 | 1 |
| Span | logTG | 0.0019 | 11 | 1 |
| Span | TG_HDL | 0.0019 | 10 | 1 |
| Span | VAI | 0.0012 | 8 | 1 |
| Visual_scaled | HDL | 0.0017 | 12 | 1 |
| Visual_scaled | logTG | 0.0008 | 11 | 1 |
| Visual_scaled | TG_HDL | 0.0006 | 10 | 1 |
| Visual_scaled | VAI | 0.0003 | 8 | 1 |

Table S15: Mendelian randomization horizontal pleiotropy results

| **Outcome** | **Exposure** | **Egger_intercept** | **SE** | **pval** |
| --- | --- | --- | --- | --- |
| Auditory_scaled | HDL-C | 0.047 | 3.327 | 0.989 |
| Auditory_scaled | logTG | -0.043 | 2.131 | 0.984 |
| Auditory_scaled | TG:HDL | -0.035 | 2.180 | 0.987 |
| Auditory_scaled | VAI | 0.012 | 3.397 | 0.997 |
| Comprehension_scaled | HDL-C | 0.036 | 3.311 | 0.992 |
| Comprehension_scaled | logTG | -0.012 | 2.119 | 0.996 |
| Comprehension_scaled | TG:HDL | 0.011 | 2.174 | 0.996 |
| Comprehension_scaled | VAI | -0.093 | 3.379 | 0.979 |
| DelayedRecall_scaled | HDL-C | 0.039 | 3.310 | 0.991 |
| DelayedRecall_scaled | logTG | -0.025 | 2.118 | 0.991 |
| DelayedRecall_scaled | TG:HDL | 0.001 | 2.172 | 1.000 |
| DelayedRecall_scaled | VAI | 0.034 | 3.376 | 0.992 |
| Geo_Fig_scaled | HDL-C | 0.046 | 3.299 | 0.989 |
| Geo_Fig_scaled | logTG | -0.051 | 2.110 | 0.981 |
| Geo_Fig_scaled | TG:HDL | -0.013 | 2.163 | 0.995 |
| Geo_Fig_scaled | VAI | 0.016 | 3.364 | 0.996 |
| ImmediateRecall_scaled | HDL-C | 0.053 | 3.344 | 0.988 |
| ImmediateRecall_scaled | logTG | -0.023 | 2.140 | 0.992 |
| ImmediateRecall_scaled | TG:HDL | -0.012 | 2.194 | 0.996 |
| ImmediateRecall_scaled | VAI | 0.033 | 3.411 | 0.993 |
| MRT | HDL-C | 0.083 | 3.305 | 0.980 |
| MRT | logTG | -0.020 | 2.121 | 0.993 |
| MRT | TG:HDL | 0.012 | 2.174 | 0.996 |
| MRT | VAI | -0.004 | 3.393 | 0.999 |
| NameFaceAssocFace_scaled | HDL-C | 0.063 | 3.309 | 0.985 |
| NameFaceAssocFace_scaled | logTG | -0.013 | 2.116 | 0.995 |
| NameFaceAssocFace_scaled | TG:HDL | -0.004 | 2.170 | 0.999 |
| NameFaceAssocFace_scaled | VAI | -0.008 | 3.372 | 0.998 |
| NameRecog_scaled | HDL-C | 0.006 | 3.335 | 0.999 |
| NameRecog_scaled | logTG | 0.034 | 2.133 | 0.987 |
| NameRecog_scaled | TG:HDL | 0.031 | 2.187 | 0.989 |
| NameRecog_scaled | VAI | 0.060 | 3.398 | 0.986 |
| Naming_Assoc_scaled | HDL-C | -0.042 | 3.310 | 0.990 |
| Naming_Assoc_scaled | logTG | -0.017 | 2.121 | 0.994 |
| Naming_Assoc_scaled | TG:HDL | -0.002 | 2.171 | 0.999 |
| Naming_Assoc_scaled | VAI | 0.027 | 3.380 | 0.994 |
| Phonetic | HDL-C | 0.041 | 3.287 | 0.990 |
| Phonetic | logTG | 0.011 | 2.104 | 0.996 |
| Phonetic | TG:HDL | 0.010 | 2.155 | 0.996 |
| Phonetic | VAI | 0.002 | 3.351 | 1.000 |
| Reading_scaled | HDL-C | -0.036 | 3.310 | 0.991 |
| Reading_scaled | logTG | -0.014 | 2.118 | 0.995 |
| Reading_scaled | TG:HDL | -0.001 | 2.172 | 1.000 |
| Reading_scaled | VAI | -0.009 | 3.380 | 0.998 |
| Semantic | HDL-C | 0.006 | 3.323 | 0.999 |
| Semantic | logTG | 0.013 | 2.125 | 0.995 |
| Semantic | TG:HDL | 0.022 | 2.178 | 0.992 |
| Semantic | VAI | 0.051 | 3.387 | 0.989 |
| Span | HDL-C | 0.008 | 3.281 | 0.998 |
| Span | logTG | 0.030 | 2.104 | 0.989 |
| Span | TG:HDL | 0.035 | 2.156 | 0.987 |
| Span | VAI | 0.011 | 3.353 | 0.998 |
| Visual_scaled | HDL-C | 0.053 | 3.327 | 0.988 |
| Visual_scaled | logTG | 0.014 | 2.136 | 0.995 |
| Visual_scaled | TG:HDL | 0.033 | 2.185 | 0.988 |
| Visual_scaled | VAI | 0.057 | 3.398 | 0.987 |

Table S16: Steiger’s directionality test reporting causal direction

| **outcome** | **exposure** | **snp_r2.exposure** | **snp_r2.outcome** | **correct_causal_direction** | **steiger_pval** |
| --- | --- | --- | --- | --- | --- |
| Auditory attention | HDL-C | 0.17789262 | 0.00247354 | TRUE | 1.3441E-65 |
| Auditory attention | logTG | 0.20090295 | 0.00330042 | TRUE | 8.9271E-74 |
| Auditory attention | TG:HDL | 0.18502185 | 0.00313508 | TRUE | 7.3064E-67 |
| Auditory attention | VAI | 0.16813789 | 0.00293613 | TRUE | 3.8377E-59 |
| Comprehension_scaled | HDL-C | 0.17789262 | 0.00299083 | TRUE | 9.8885E-71 |
| Comprehension_scaled | logTG | 0.20090295 | 0.00254985 | TRUE | 3.6343E-84 |
| Comprehension_scaled | TG:HDL | 0.18502185 | 0.00311589 | TRUE | 6.1728E-74 |
| Comprehension_scaled | VAI | 0.16813789 | 0.00197885 | TRUE | 1.6016E-68 |
| DelayedRecall_scaled | HDL-C | 0.17789262 | 0.00230388 | TRUE | 4.0601E-72 |
| DelayedRecall_scaled | logTG | 0.20090295 | 0.00366944 | TRUE | 2.739E-79 |
| DelayedRecall_scaled | TG:HDL | 0.18502185 | 0.00394453 | TRUE | 1.6519E-70 |
| DelayedRecall_scaled | VAI | 0.16813789 | 0.00366251 | TRUE | 2.5113E-62 |
| Geo_Fig_scaled | HDL-C | 0.17789262 | 0.00525614 | TRUE | 9.3366E-65 |
| Geo_Fig_scaled | logTG | 0.20090295 | 0.00750227 | TRUE | 4.7522E-71 |
| Geo_Fig_scaled | TG:HDL | 0.18502185 | 0.00767353 | TRUE | 4.1532E-63 |
| Geo_Fig_scaled | VAI | 0.16813789 | 0.00740086 | TRUE | 3.7608E-55 |
| ImmediateRecall_scaled | HDL-C | 0.17789262 | 0.00144622 | TRUE | 1.2761E-76 |
| ImmediateRecall_scaled | logTG | 0.20090295 | 0.00746603 | TRUE | 6.0795E-71 |
| ImmediateRecall_scaled | TG:HDL | 0.18502185 | 0.00737686 | TRUE | 1.6267E-63 |
| ImmediateRecall_scaled | VAI | 0.16813789 | 0.00732058 | TRUE | 3.7082E-55 |
| MRT | HDL-C | 0.17789262 | 0.00378729 | TRUE | 6.9616E-64 |
| MRT | logTG | 0.20090295 | 0.00155955 | TRUE | 1.3269E-82 |
| MRT | TG:HDL | 0.18502185 | 0.00205018 | TRUE | 1.186E-72 |
| MRT | VAI | 0.16813789 | 0.00137256 | TRUE | 1.7445E-66 |
| NameFaceAssocFace_scaled | HDL-C | 0.17789262 | 0.0057229 | TRUE | 1.6629E-63 |
| NameFaceAssocFace_scaled | logTG | 0.20090295 | 0.00208509 | TRUE | 6.5319E-86 |
| NameFaceAssocFace_scaled | TG:HDL | 0.18502185 | 0.00191057 | TRUE | 3.1399E-78 |
| NameFaceAssocFace_scaled | VAI | 0.16813789 | 0.00160669 | TRUE | 6.0477E-70 |
| NameRecog_scaled | HDL-C | 0.17789262 | 0.00229939 | TRUE | 6.6756E-73 |
| NameRecog_scaled | logTG | 0.20090295 | 0.00638113 | TRUE | 4.2227E-73 |
| NameRecog_scaled | TG:HDL | 0.18502185 | 0.00560794 | TRUE | 4.4192E-67 |
| NameRecog_scaled | VAI | 0.16813789 | 0.0050618 | TRUE | 1.5074E-59 |
| Naming_Assoc_scaled | HDL-C | 0.17789262 | 0.00524421 | TRUE | 8.0096E-63 |
| Naming_Assoc_scaled | logTG | 0.20090295 | 0.0064621 | TRUE | 4.8993E-71 |
| Naming_Assoc_scaled | TG:HDL | 0.18502185 | 0.00651751 | TRUE | 1.9986E-63 |
| Naming_Assoc_scaled | VAI | 0.16813789 | 0.00617296 | TRUE | 9.3428E-56 |
| Phonetic | HDL-C | 0.17789262 | 0.00285561 | TRUE | 1.3154E-69 |
| Phonetic | logTG | 0.20090295 | 0.00120148 | TRUE | 2.5103E-88 |
| Phonetic | TG:HDL | 0.18502185 | 0.00125824 | TRUE | 1.3431E-79 |
| Phonetic | VAI | 0.16813789 | 0.00060156 | TRUE | 7.2332E-74 |
| Reading_scaled | HDL-C | 0.17789262 | 0.00277655 | TRUE | 1.9382E-71 |
| Reading_scaled | logTG | 0.20090295 | 0.00638648 | TRUE | 2.6568E-73 |
| Reading_scaled | TG:HDL | 0.18502185 | 0.0060106 | TRUE | 2.2393E-66 |
| Reading_scaled | VAI | 0.16813789 | 0.00530477 | TRUE | 3.5108E-59 |
| Semantic | HDL-C | 0.17789262 | 0.00091218 | TRUE | 9.6684E-80 |
| Semantic | logTG | 0.20090295 | 0.0038241 | TRUE | 4.404E-80 |
| Semantic | TG:HDL | 0.18502185 | 0.00375051 | TRUE | 3.487E-72 |
| Semantic | VAI | 0.16813789 | 0.00345119 | TRUE | 6.8957E-64 |
| Span | HDL-C | 0.17789262 | 0.00071926 | TRUE | 1.8413E-73 |
| Span | logTG | 0.20090295 | 0.00293594 | TRUE | 3.0863E-75 |
| Span | TG:HDL | 0.18502185 | 0.00290109 | TRUE | 7.253E-68 |
| Span | VAI | 0.16813789 | 0.00183878 | TRUE | 6.9336E-63 |
| Visual_scaled | HDL-C | 0.17789262 | 0.00174329 | TRUE | 4.3584E-67 |
| Visual_scaled | logTG | 0.20090295 | 0.00130237 | TRUE | 6.6453E-80 |
| Visual_scaled | TG:HDL | 0.18502185 | 0.001224 | TRUE | 1.2332E-72 |
| Visual_scaled | VAI | 0.16813789 | 0.00086136 | TRUE | 8.2359E-66 |
